# Genetic Evidence of an East Asian Origin and Paleolithic Northward Migration of Y-chromosome Haplogroup N

**DOI:** 10.1371/journal.pone.0066102

**Published:** 2013-06-20

**Authors:** Hong Shi, Xuebin Qi, Hua Zhong, Yi Peng, Xiaoming Zhang, Runlin Z. Ma, Bing Su

**Affiliations:** 1 State Key Laboratory of Genetic Resources and Evolution, Kunming Institute of Zoology, Chinese Academy of Sciences, Kunming, China; 2 Center for Developmental Biology, Institute of Genetics and Developmental Biology, Chinese Academy of Sciences, Beijing, China; 3 University of Chinese Academy of Sciences, Beijing, China; University of Cambridge, United Kingdom

## Abstract

The Y-chromosome haplogroup N-M231 (Hg N) is distributed widely in eastern and central Asia, Siberia, as well as in eastern and northern Europe. Previous studies suggested a counterclockwise prehistoric migration of Hg N from eastern Asia to eastern and northern Europe. However, the root of this Y chromosome lineage and its detailed dispersal pattern across eastern Asia are still unclear. We analyzed haplogroup profiles and phylogeographic patterns of 1,570 Hg N individuals from 20,826 males in 359 populations across Eurasia. We first genotyped 6,371 males from 169 populations in China and Cambodia, and generated data of 360 Hg N individuals, and then combined published data on 1,210 Hg N individuals from Japanese, Southeast Asian, Siberian, European and Central Asian populations. The results showed that the sub-haplogroups of Hg N have a distinct geographical distribution. The highest Y-STR diversity of the ancestral Hg N sub-haplogroups was observed in the southern part of mainland East Asia, and further phylogeographic analyses supports an origin of Hg N in southern China. Combined with previous data, we propose that the early northward dispersal of Hg N started from southern China about 21 thousand years ago (kya), expanding into northern China 12–18 kya, and reaching further north to Siberia about 12–14 kya before a population expansion and westward migration into Central Asia and eastern/northern Europe around 8.0–10.0 kya. This northward migration of Hg N likewise coincides with retreating ice sheets after the Last Glacial Maximum (22–18 kya) in mainland East Asia.

## Introduction

In recent years, extensive studies of the Y-chromosome lineages in East Asian populations have been conducted and found that the dominant haplogroups O-M175, D-M174, C-M130, and N-M231 in East Asian populations all have a southern origin [1]–[8]. Among these East Asian Y-chromosome lineages, D-M174 represents the earliest northward migration, beginning from the southern part of East Asia of what is now mainland Southeast Asia and southern China about 50–60 kya [5]. The northward migration of C-M130 occurred about 40 kya, following coastal route up mainland China, then reaching further north to Siberia around 15 kya and finally making its way to northern America [8]–[11]. The northward expansion of O-M175 within the Asian continent (about 25–30 kya) made the greatest impact on current East Asian Y chromosomal profiles, reflected by the dominance of O-M175 lineages (ranging anywhere from 18–75%) in East Asia, and both mainland and island Southeast Asia [4].

By contrast, N-M231, as a sister-clade of O-M175, is relatively less prevalent in East Asian populations (averaging around 6%) (Table 1), but has a much wider geographic distribution across Eurasia as compared with the other Y-chromosome haplogroups [3], [7], [12]–[29]. Rootsi et al. (2007) proposed that the Hg N lineage dispersed from East Asia to northwestern Europe following a counter-clock-wise migratory route and speculated that the original homeland of Hg N likely traced to Southeast Asia, and had split with O-M175 about 34 kya. However, due to the limited populations studied for N-M231 from East Asia and Southeast Asia, Hg N’s putative center of origin and the chronology of dispersals remain inconclusive.

**Table 1:** Distribution of Hg N in Eurasian populations.

Region	Populations	Size	N-M231	N%	References
***Europe***	South Europeans	1579	0	0	Rootsi,*et al*, 2007; Capelli,*et al*.2007; King,*et al.*2008; Martinez,*et al.*2007
	West Europeans	361	0	0	Rootsi,*et al*, 2007; Gusmao,*et al*.2008; López-Parra,*et al*.2009
	North Europeans	3595	1267	35.24	Rootsi,*et al*, 2007; Mirabal,*et al*.2009; Balanovsk,*et al.*2008; Lappalainen, *et al*, 2006; 2008
	East Europeans	2508	510	20.33	Derenko,*et al*, 2007; Rootsi,*et al*, 2007
	Caucasus (pooled)	1404	3	0.21	Rootsi,*et al*, 2007
***West Asia***	Turks	523	20	3.82	Rootsi,*et al*, 2007
	Iranians	185	0	0	Derenko,*et al*, 2007; Rootsi, *et al*, 2007
	West Asians	668	23	3.44	Cinnioğlu,*et al*.2004; Regueiro,*et al*.2006
***North Asia***	Siberians	3381	1294	38.27	Derenko,*et al*, 2007; Rootsi,*et al,* 2007; Sengupta,*et al*.2006; Hammer,*et al.*2006
***Central Asia***	Central Asians	824	53	6.43	Derenko,*et al*, 2007; Rootsi,*et al*, 2007; Zhong, *et al.* 2011
***East Asia***	Koreans	297	10	3.37	Hammer,et al.2006; Derenko,et al, 2007; Rootsi,et al, 2007; Zhong, et al. 2011; present study
	Japanese	877	16	1.82	Rootsi,*et al*, 2007; Hammer,*et al*.2006; Nonaka,*et al*.2007
	Altai (Northeastern China)	874	78	8.92	Hammer,*et a*l.2006; Derenko,*et al*, 2007; Rootsi,*et al*, 2007; Zhong, *et al*. 2011; present study
	Altai (Northwestern China)	377	13	3.45	present study
	Tibetans	2459	147	5.98	Rootsi, *et al*, 2007; present study
	Northern Han	947	69	7.29	Rootsi,*et al*, 2007; Zhong, *et al*. 2011; present study
	Southern Han	1114	82	7.36	Hammer,*et al.*2006; Rootsi,*et al*, 2007; Zhong, *et al.* 2011; present study
	Taiwan Aborigines	139	1	0.72	Hammer,*et al*.2006; Rootsi,*et al*, 2007
	Taiwan Chinese	110	6	5.45	Rootsi,*et al*, 2007
	Tibeto-Burmans (Southwestern China)	409	57	13.94	Rootsi,*et al*, 2007; Zhong, *et al.* 2011; present study
	Hmong-Miens (Southwestern China)	477	6	1.26	Rootsi,*et al*, 2007; Zhong, *et al.* 2011; present study
	Daic people (Southwestern China)	528	17	3.22	Rootsi,*et al*, 2007; Zhong, *et al.* 2011; present study
	Austro-Asiatic people (Southwestern China)	155	16	10.32	Zhong, *et al*. 2011; present study
***Southeast Asia***	Cambodians	371	1	0.27	Rootsi,*et al*, 2007; present study
	Laotians	803	4	0.50	Cai,*et al*, 2011; He,*et al,* 2012
	Vietnamese	285	4	1.40	Rootsi,*et al*, 2007; He,*et al*, 2012
	Thai	17	0	0	He,*et al*, 2012
	Indonesian	2291	2	0.09	Rootsi,*et al*, 2007; Karafet,*et al*, 2010
	Malaysians	72	0	0	Rootsi,*et al*, 2007; Karafet,*et al*, 2010
	Filipinos	135	0	0	Rootsi,*et al*, 2007; Karafet,*et al*, 2010
	Southeast Asians	230	3	1.30	Hammer,*et al.*2006
***South Asia***	South Asians	2505	2	0.08	Rootsi,*et al*, 2007; Sengupta,*et al*.2006; Gayden,*et al*.2007
***Oceania***	Oceanians	646	0	0	Rootsi,*et al*, 2007

In the present study, we systematically analyzed Hg N profiles in East Asia and Southeast Asia populations (a total of 6,371 males from 169 geographic populations) to trace the origin and prehistoric migration patterns of the Hg N lineage.

## Materials and Methods

### Samples

**Figure 1:**
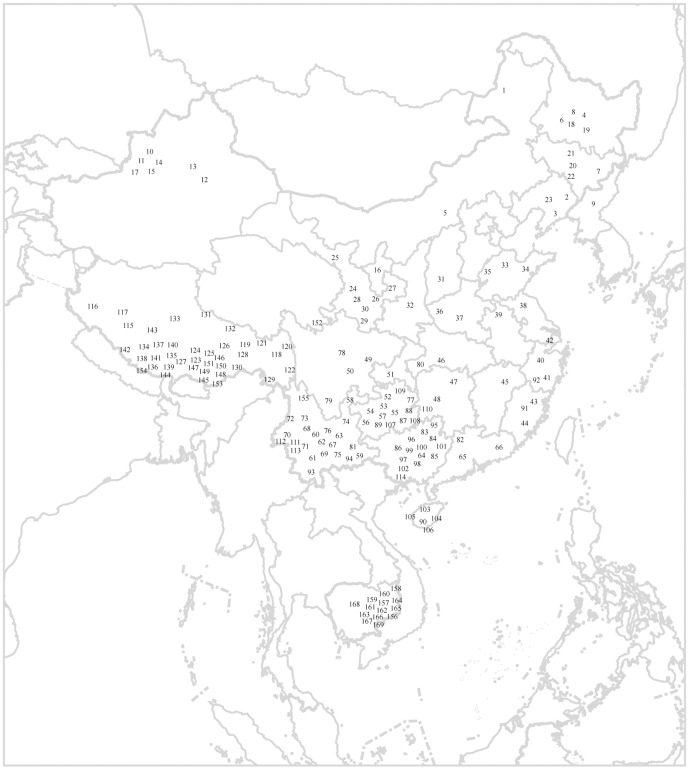
Geographic locations of 169 studied populations. Population details are given in Table S1.

A total of 6,371 unrelated males from 169 populations in East Asia (Figure 1 and Table S1) were recruited and asked to sign written informed consent for the usage of samples in this study. The protocol of this study was approved by the Institutional Review Board of Kunming Institute of Zoology, Chinese Academy of Sciences (Approval ID number, SWYX-2012008). In addition, to compare the population structure of Y chromosome Hg N among geographic populations, we also retrieved previously published data on 1,210 Hg N individuals from different geographic areas (Y-SNP and Y-STR) [3], [12], [13], [16], [19], [28], [29].

### Y-Chromosome Marker Genotyping

According to the hierarchical genotyping strategy, M231 was typed first and samples from the M231-positive individuals were then subjected to further subtyping, according to the high-resolution Y chromosomal haplogroup tree so that they could be assigned to a specific haplotype [30]. The Y chromosome bi-allelic markers (LLY22g, M128, P43 and M46 (Tat)) were genotyped by the Snapshot method (Applied Biosystems, USA). Additionally, the 7 commonly used Y-STR markers: DYS19, DYS389I, DYS389II, DYS390, DYS391, DYS392, and DYS393were also typed using fluorescence-labeled primers on an ABI 3130XL Genetic Analyzer (Applied Biosystems, USA). The Y-STR nomenclature follows a system proposed previously [31].

### Data Analysis

To visualize the geographic distributions of Hg N and its sub-lineages, Golden Software Surfer 10.0 (Golden Software Inc., USA) with the Kriging algorithm was used to construct a contour map, and the data used was listed in Table S3.

Median-joining networks for STR variations of the Y-chromosomal haplogroups were constructed using NETWORK 4.6 (Fluxus Engineering) [32] with equal weights across loci.

For each Y-chromosomal haplogroup/sub-haplogroup (defined by Y-SNPs), we estimated its age by Y-STR variations using the published method [27], [33], [34]. An effective mutation rate of 0.0069 was used [34].

The genetic diversity of the different geographic populations under Hg N and its sub-haplogroups were calculated using STR data by GenAlEx 6.5 [35].

For the analysis of Y-chromosomal STR alleles, DYS389II was named DYS389b after subtracting DYS389I because the PCR product of DYS389II contains both DYS389II and DYS389I loci.

## Results

We systematically screened a total of 6,371 unrelated males from 169 populations in China and Cambodia (Figure 1 and Table S1). By genotyping the Y-chromosome bi-allelic marker M231, we identified 390 males (6.12%) belonging to this Hg N lineage. Further typing of 4 additional bi-allelic markers and 7 Y-chromosome STRs, generated complete data for 360 Hg N males, which were used in the following analyses (Table 2). We also retrieved 1,210 Hg N data from other published studies, including 1,197 Hg N males identified from 68 populations in Siberia, Central Asia and Europe [3], [12], [16], [28], [29], and 13 Hg N males from 4 populations in Japan, Laos and southern China [3], [13], [19]. Collectively, we analyzed a total of 1,570 Hg N, covering all major geographic regions possessing the Hg N lineage (from 20,826 males in 359 populations across Eurasia, Table S2).

**Table 2:** Distribution of Hg N sub-haplogroups in eastern Asia.

Population	Sample size	N%	N*-M231	N1*-LLY22g	N1a-M128	N1b-P43	N1c-M46
Altai (Northeastern China)	198	10.10		4.55	1.01	2.02	2.53
Altai (Northwestern China)	377	7.43	0.53	2.12		0.27	0.53
Koreans	64	6.25		3.13	1.56	1.56	
Northern Han	853	6.80	0.23	4.22	0.47		1.64
Southern Han	876	6.74	1.26	3.54	0.57	0.11	0.80
Tibetans	2442	5.90	0.04	5.32	0.08	0.04	0.41
Tibeto-Burmans	325	12.92	0.62	7.38	3.08		0.92
Hmong-Miens	308	1.95	0.32	0.65	0.32		0.65
Daic people	463	3.67	1.51	1.94			0.22
Austro-Asiatic people (Southwestern China)	100	11.00		5.00			
Austro-Asiatic people (Cambodian)	293	0.34		0.34			
Austronesians	72						

Note: samples were merged by language families.

**Figure 2:**
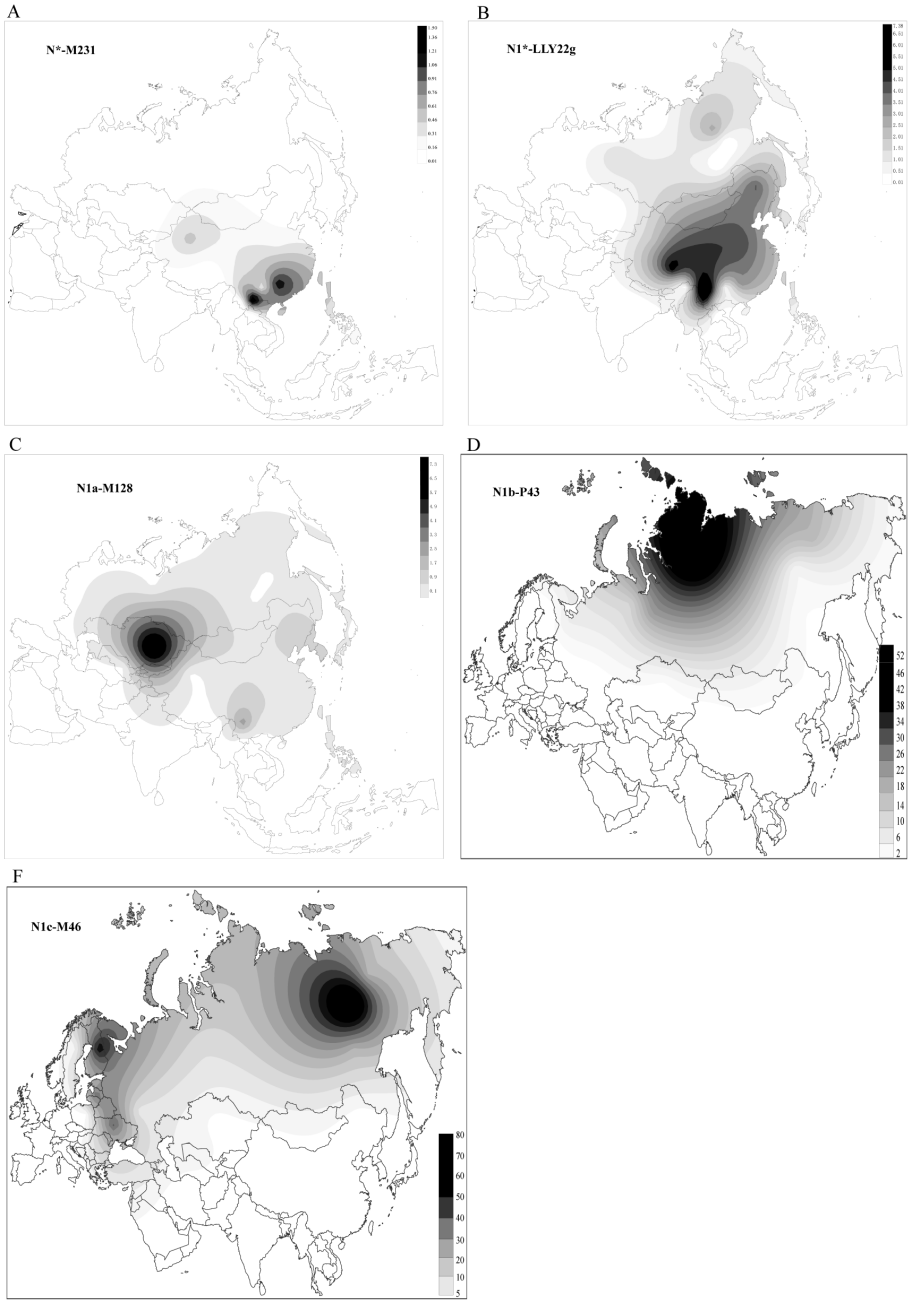
GContour maps of Hg N sub-haplogroups. A, N*-M231, B, N1*-LLY22g, C, N1a-M128, D, N1b-P43, E, N1c-M46 (Tat). (The regional populations used is listed in Table S3).

**Figure 3:**
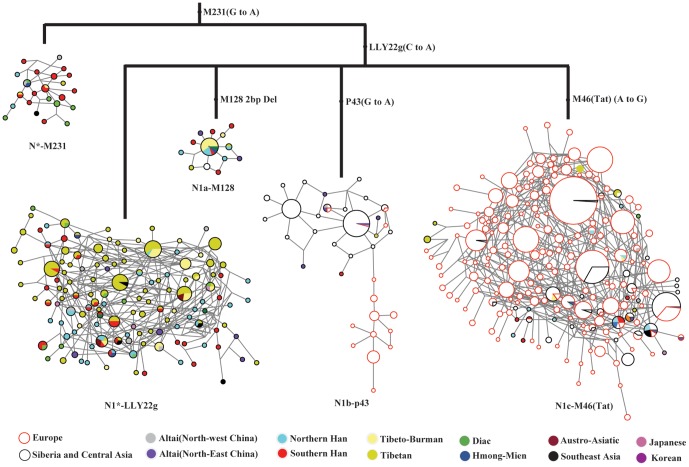
Median-joining networks for sub-haplogroups of Hg N lineage using Y-STR alleles. The diagnostic mutations used to classify the sub-haplogroups are labeled on the tree branches. Each node represents a haplotype and its size is proportional to the haplotype frequency, and the length of a branch is proportional to the mutation steps. The colored areas indicate the geographic origins of the studied populations or language groups.

Hg N is prevalent (>5%) in East Asia (e.g., among Han Chinese, Tibeto-Burman and Austro-Asiatic speaking populations), as well as in northern/central Asia and eastern/northern Europe with on average the highest frequency in Siberia (38.27%). Meanwhile, Hg N is relatively rare in southeastern, southern and western Asia, and completely absent in southern/western Europe. Within the Hg N lineage, there are 5 sub-haplogroups with distinctive geographic distributions. N*-M231 is presumably the ancestral haplogroup in Hg N, mostly present in southern East Asian populations including Daic, southern Han Chinese, Tibeto-Burman and Hmong-Mien in southern China (Figure 2A); however, it is totally absent in Siberia, Central Asia and eastern/northern Europe, consistent with the previously proposed southern origin of Hg N in East Asia [3], [12], [16], [28], [29]. The other 4 sub-haplogroups share a common mutation at the LLY22g locus (Figure 3). Under LLY22g, N1*-LLY22g is both the ancestral and most dominant sub-haplogroup, with distribution extending from southern to northern East Asia and the highest frequency observed in Tibeto-Burman populations. The distribution pattern of N1a-M128 is similar to N1*-LLY22g, but much less prevalent (Figure 2B and 2C). By contrast, the distributions of N1b-P43 and N1c-M46 are restricted to North Asia and East/North Europe, rare in East Asia and Central Asia, and absent in Southeast and South Asia (Figure 2D and 2E). Collectively, this geographic distribution pattern suggests a clear divergence between regional populations with the ancestral lineages occurring in multiple ethnic populations throughout southern China.

We constructed contour maps of the five N-M231 sub-haplogroups based on the geographic distributions of these lineages in Eurasian populations (Table S3). The two presumably ancestral haplogroups (N*-M231 and N1*-LLY22g) likely originated in southern China, as there is a clear south-to-north decline of these frequencies (Figure 2A and 2B). Conversely, N1b-P43 and N1c-M46 are both enriched in Siberia with N1b-p43 having a north-to-south decline and N1c-M46 having an east-to-west decline (Figure 2D and 2E). The contour map of N1a-M128 is different from the others with the highest frequency observed in Central Asia due to the relatively high frequency of N1a-M128 among Kazakhs (8.1%) in Central Asia (Figure 2C).

To examine the detailed diversity of each N-M231 sub-haplogroup, we constructed STR networks for the 5 sub-haplogroups based on data of 7 Y-chromosome STR loci (Figure 3). Among the two ancestral lineages of Hg N, we observed relatively diverged STR haplotypes, and the core STR haplotypes are mostly from southern populations in China, suggesting a likely origin in southern China. Comparatively, the core STR haplotypes of N1b-P43 are mostly from the northern populations of China and Siberia, suggesting its origin may be in northern East Asia. Moreover, the STR networks of N1b-P43 reflect that the STR haplotypes in Europeans were derived from Siberia and Central Asia, consistent with the proposed counter-clock-wise prehistoric migration of the Hg N lineages into East/North Europe [3]. Interestingly, N1a-M128 displayed a star-like STR network, implying a recent expansion of this Hg N lineage. Although N1a-M128 has the highest frequency in Central Asia [3], considering its presence (though low frequency) in multiple ethnic populations throughout southern China, N1a-M128 is unlikely to have a Central Asia origin. Instead, N1a-M128 may similarly have its origin in East Asia, reflected by the STR network showing an East Asia core haplotype (Figure 3). The high frequency of N1a-M128 in Central Asia is likely then due to a recent local expansion of this sub-haplogroup.

Further comparison of the STR variation levels among the different populations also supports an East Asia origin of the Hg N. For the two ancestral lineages, N*-M231 and N1*-LYY22g, the STR diversity of southern populations is higher than northern populations in East Asia (Table 3). We observed similar patterns for the other three sub-haplogroups, which expanded outside of East Asia and into Siberia, Central Asia and East/North Europe (Table 3). Unfortunately, due to the limited sample sizes used to calculate the STR diversity of different Hg N haplotypes, we are cautious of making any definitive conclusions from STR diversity level data.

**Table 3:** Y-STRs diversity of Hg N sub-haplogroups.

Haplogroup	Populations	Sample size	Y-STRs diversity ± SE
N*	Northern Chinese	4	0.268±0.100
	Southern Chinese	27	0.332±0.070
N1*	Altai (Northeastern China)	18	0.437±0.065
	Han Chinese (mainland China)	68	0.506±0.056
	Tibeto-Burmans (Southwestern China)	154	0.437±0.063
	Hmong-Meins, Daic and Austro-Asiatic people (Southwestern China)	18	0.475±0.050
N1a	Altai (Northwestern China)	5	0.206±0.076
	Han Chinese (mainland China)	11	0.201±0.051
	Tibeto-Burmans (Southwestern China)	12	0.087±0.031
N1b	Altai (Northwestern China)	6	0.286±0.056
	Siberians	92	0.193±0.071
	Europeans	38	0.303±0.084
N1c	Altai (Northwestern China)	8	0.286±0.066
	Han Chinese (mainland China)	21	0.277±0.074
	Tibeto-Burmans (Southwestern China)	13	0.519±0.021
	Hmong-Meins, Daic and Austro-Asiatic people (Southwestern China)	6	0.143±0.071
	Siberians	119	0.283±0.054
	Europeans	944	0.352±0.055

In order to date the major prehistoric population events along the northward and westward migration routes of the Hg N lineages, we used the STR data to calculate the STR variation ages of the 5 Hg N sub-haplogroups (Table 4). As expected, the ancestral lineage under LLY22g (N1*-LLY22g), the oldest among all N-M231 sub-haplogroups, was dated to 21.66 kya, falling in the Upper Paleolithic. The age of N1b-P43 was also very old (18.90 kya), indicating a relatively rapid northward migration during the Paleolithic period from southern China northward into Siberia. N1c-M46 was relatively young (11.70 kya). The age of N*-M231 (13.69 kya), presumably the ancestral lineage of Hg N, is younger than expected, likely as a result of yet-to-be-identified individuals having derived N-M231 sub-haplogroup when new Y SNP markers are uncovered in the future. By comparison, the age of N1a-M128 is strikingly young (3.75 kya), consistent with the observed star-like STR network suggesting a recent expansion of this lineage (Figure 3). Because the reported Central Asian population (Kazakhs) possessing relatively high frequency of N1a-M128 did not have enough STR data to calculate diversity, we were unable to infer the time of N1a-M128’s migration from East Asia into Central Asia.

**Table 4:** Estimated ages of Hg N and its sub-haplogroups.

Haplogroup	Sample size	Age of STR variation (Kya ± SE)
**N-M231**	**1566**	**16.42±0.94**
N*-M231	31	13.69±3.37
N1*-LLY22g	258	21.66±4.48
N1a-M128	28	3.75±0.94
N1b-P43	136	18.90±7.73
N1c-M46	1111	11.70±1.87

## Discussion

Hg N is the most widely distributed Y chromosome haplogroup in Eurasia (Table 1). By extending the population coverage into East Asia, we showed that Hg N is present in most East Asian populations, though the frequencies are low (Table 1 and Table S1). Previously, Hg N was speculated to have originated in Southeast Asia, and consequently split with its sister haplogroup O-M122 about 34 kya and then migrated northward to mainland East Asia during late Pleistocene-Holocene [3]. However, we demonstrated that Hg N is in fact extremely rare in Southeast Asia populations. For example, in our analysis of 293 multi-ethnic Cambodian males, we only detected one Hg N individual (0.34%), contrasting the previous report of a much higher frequency of one in six males (16.67%) in Cambodia, which was likely caused by a small sample size. Hg N is also rare in other Southeast Asia populations (<1.5%), including those in Laos, Vietnam, Thailand, Indonesia, Malaysia and the Philippines (Table 1), thereby suggesting that Southeast Asia may not be the homeland of Hg N. Instead, the southern part of mainland East Asia (presumably southern China) is more likely the putative origin for Hg N, as reflected by the distribution of ancestral Hg N lineages (N*-M231 and N1*-LLY22g) and the observed higher STR diversity of multiple southern ethnic populations in China (Table 3). The STR network analysis and contour map further support a southern East Asia origin of Hg N.

As proposed previously, the initial prehistoric migration of Hg N began in the south and moved south to north, starting in southern China. We are now able to draw a relatively more detailed migratory picture for Hg N lineage by estimating the ages of the Hg N haplotypes using STR variations. The initial northward migration probably started around 21 kya, reflected by the age of N1*-LLY22g (21.66 kya), the most prevalent N-M231 sub-haplogroup in East Asia. Along the path of northward migration in mainland China, two other N-M231 sub-haplogroups occurred at about 12–18 kya, later becoming the dominant Y-chromosome lineages in Siberian populations as a result of local population expansion. Previously N1b-P43 and N1c-M46 were proposed to have experienced serial bottleneck events in northern East Asia and then dispersed into Siberia, Central Asia and Europe [3]. As the age difference between N1b-P43/N1c-M46 and N1*-LLY22g is comparatively small (3–5 kya), we can infer that the prehistoric migration of Hg N was relatively quick, coinciding with the end of the Last Glacial Maximum (LGM) in East Asia (22–18 kya). The postglacial migration of modern humans in East Asia can likewise be reflected by the northward migration of the C-M130 haplogroup along the coastline of mainland China, before moving further north to Siberia around 15 kya [8]–[11].

With the application of next generation sequencing on the Y chromosome, more Y-SNPs will be discovered, which can help increase the resolution of the Hg N haplogroup tee and provide more detailed phylogeographic information about the origin and prehistoric migration of this important Eurasian Y chromosome lineage.

## Conclusion

**Figure 4:**
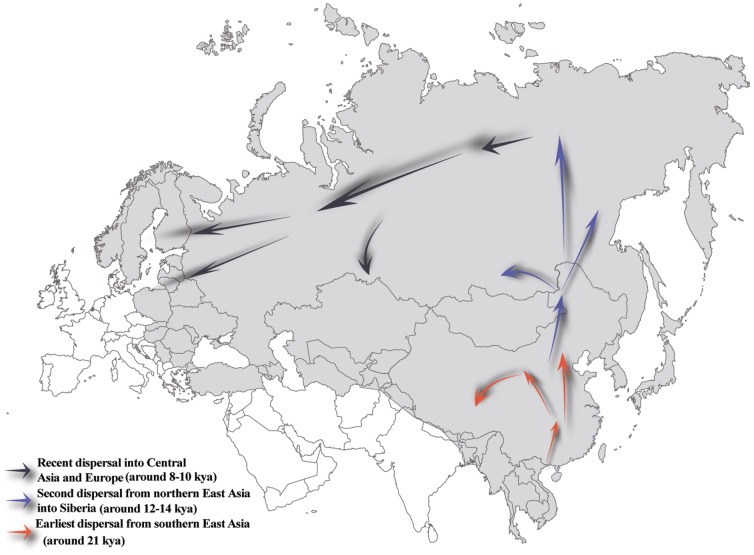
Proposed prehistoric migration routes for Hg N lineage. The shaded areas represent the haplogroup N distributions.

Based on the dating of the Hg N haplotypes and their geographic distributions paired with the suggested counter-clock-wise migratory route across Eurasia [3], we proposed a migratory map (Figure 4) of the Hg N lineages beginning in southern China about 21 kya, and expanding into northern China 12–18 kya, reaching further north to Siberia about 12–14 kya [3], and followed by a population expansion and westward migration into Central Asia and East/North Europe around 8.0–10.0 kya [16].

## Supporting Information

Table S1 **The 169 sampled populations in this study.**
(DOCX)

Table S2 **The STR genotyping data of Hg N samples.**
(DOCX)

Table S3 **The populations information used to constructe contour maps.**
(DOCX)
