# Supplementary material for: Genetic Evidence of an East Asian Origin and Paleolithic Northward Migration of Y-chromosome Haplogroup N
Source: PLoS One. 2013 Jun 20;8(6):e66102. doi: 10.1371/journal.pone.0066102 (PMC3688714; doi:10.1371/journal.pone.0066102)
Supplement: Table S1 — The 169 sampled populations in this study. (DOCX) [file pone.0066102.s001.docx]

Table S1. The 169 sampled populations in this study.

| Location | Sample | Population Group | Size | M231 | Location | Sample | Population Group | Size | M231 |
| --- | --- | --- | --- | --- | --- | --- | --- | --- | --- |
| 1 | Ewenki (Inner Mongolia) | Altaic(North-east China) | 31 | 4 | 86 | Yao (Guangxi) | Hmong-Mien | 55 |  |
| 2 | Man (Liaoning) | Altaic(North-east China) | 43 | 5 | 87 | Miao (Guizhou) | Hmong-Mien | 45 | 1 |
| 3 | Man (Liaoning) | Altaic(North-east China) | 66 | 6 | 88 | Miao (Guizhou) | Hmong-Mien | 19 | 2 |
| 4 | Man (Heilongjiang) | Altaic(North-east China) | 24 | 3 | 89 | Miao (Guizhou) | Hmong-Mien | 17 |  |
| 5 | Mongolian (Inner Mongolia) | Altaic(North-east China) | 22 | 1 | 90 | Miao (Hainan) | Hmong-Mien | 20 |  |
| 6 | Mongolian (Heilongjiang) | Altaic(North-east China) | 12 | 1 | 91 | She (Fujian) | Hmong-Mien | 45 |  |
| 7 | Korean (Jilin) | Korean | 34 | 3 | 92 | She (Zhejiang) | Hmong-Mien | 17 | 1 |
| 8 | Korean (Heilongjiang) | Korean | 11 |  | 93 | Dai (Yunnan) | Daic | 18 |  |
| 9 | Korean (Korea) | Korean | 19 | 1 | 94 | Zhuang (Yunnan) | Daic | 6 |  |
| 10 | Uygur (Xinjiang) | Altaic(North-west China) | 48 | 2 | 95 | Dong (Guangxi) | Daic | 17 |  |
| 11 | Uygur (Xinjiang) | Altaic(North-west China) | 71 | 3 | 96 | Mulao (Guangxi) | Daic | 11 |  |
| 12 | Uygur (Xinjiang) | Altaic(North-west China) | 18 | 1 | 97 | Zhuang (Guangxi) | Daic | 61 | 4 |
| 13 | Uygur (Xinjiang) | Altaic(North-west China) | 50 | 4 | 98 | Buyi (Guizhou) | Daic | 34 |  |
| 14 | Hazak (Xinjiang) | Altaic(North-west China) | 53 |  | 99 | Buyi (Guizhou) | Daic | 54 |  |
| 15 | Kyrgyz (Xinjiang) | Altaic(North-west China) | 4 |  | 100 | Buyi (Guizhou) | Daic | 21 | 1 |
| 16 | Hui (Ningxia) | Altaic(North-west China) | 62 | 5 | 101 | Dong (Guizhou) | Daic | 27 | 2 |
| 17 | Xibe (Xinjiang) | Altaic(North-west China) | 61 | 11 | 102 | Li (Guizhou) | Daic | 14 | 2 |
| 18 | Han (Heilongjiang) | Chinese(Northern Han) | 67 | 2 | 103 | Li (Hainan) | Daic | 21 |  |
| 19 | Han (Heilongjiang) | Chinese(Northern Han) | 57 | 4 | 104 | Li (Hainan) | Daic | 26 |  |
| 20 | Han (Jilin) | Chinese(Northern Han) | 33 | 2 | 105 | Li (Hainan) | Daic | 17 |  |
| 21 | Han (Jilin) | Chinese(Northern Han) | 27 | 2 | 106 | Li (Hainan) | Daic | 26 | 2 |
| 22 | Han (Jilin) | Chinese(Northern Han) | 24 | 2 | 107 | Maonan (Guizhou) | Daic | 15 |  |
| 23 | Han (Liaoning) | Chinese(Northern Han) | 40 | 2 | 108 | Shui (Guizhou) | Daic | 29 |  |
| 24 | Han (Gansu) | Chinese(Northern Han) | 49 | 1 | 109 | Gelao (Guizhou) | Daic | 21 | 3 |
| 25 | Han (Gansu) | Chinese(Northern Han) | 34 | 3 | 110 | Dong (Hunan) | Daic | 45 | 3 |
| 26 | Han (Gansu) | Chinese(Northern Han) | 29 | 2 | 111 | Bulang (Yunnan) | Austro-Asiatic(south-west China) | 11 | 1 |
| 27 | Han (Gansu) | Chinese(Northern Han) | 16 |  | 112 | Deang (Yunnan) | Austro-Asiatic(south-west China) | 28 | 5 |
| 28 | Han (Gansu) | Chinese(Northern Han) | 39 | 4 | 113 | Wa (Yunnan) | Austro-Asiatic(south-west China) | 16 | 5 |
| 29 | Han (Gansu) | Chinese(Northern Han) | 20 |  | 114 | Jing (Guangxi) | Austro-Asiatic(south-west China) | 45 |  |
| 30 | Han (Gansu) | Chinese(Northern Han) | 34 | 5 | 115 | ALCQ | Tibetan | 78 | 4 |
| 31 | Han (Shanxi) | Chinese(Northern Han) | 56 | 12 | 116 | ALGJ | Tibetan | 67 | 6 |
| 32 | Han (Shannxi) | Chinese(Northern Han) | 56 |  | 117 | ALGZ | Tibetan | 66 | 10 |
| 33 | Han (Shandong) | Chinese(Northern Han) | 23 |  | 118 | CDBB | Tibetan | 17 | 2 |
| 34 | Han (Shandong) | Chinese(Northern Han) | 52 | 7 | 119 | CDDQ | Tibetan | 19 | 1 |
| 35 | Han (Shandong) | Chinese(Northern Han) | 40 | 3 | 120 | CDJD | Tibetan | 14 | 1 |
| 36 | Han (Henan) | Chinese(Northern Han) | 45 | 1 | 121 | CDLW | Tibetan | 34 | 2 |
| 37 | Han (Henan) | Chinese(Northern Han) | 21 | 2 | 122 | CDMK | Tibetan | 18 |  |
| 38 | Han (Jiangsu) | Chinese(Northern Han) | 39 |  | 123 | LSDQ | Tibetan | 274 | 14 |
| 39 | Han (Anhui) | Chinese(Northern Han) | 52 | 4 | 124 | LSGK | Tibetan | 108 | 2 |
| 40 | Han (Zhejiang) | Chinese(Southern Han) | 30 | 3 | 125 | LSLZ | Tibetan | 278 | 16 |
| 41 | Han (Zhejiang) | Chinese(Southern Han) | 55 | 5 | 126 | LSQS | Tibetan | 204 | 12 |
| 42 | Han (Shanghai) | Chinese(Southern Han) | 17 |  | 127 | LSDX | Tibetan | 215 | 12 |
| 43 | Han (Fujian) | Chinese(Southern Han) | 24 | 1 | 128 | LZBM | Tibetan | 85 | 3 |
| 44 | Han (Fujian) | Chinese(Southern Han) | 43 | 5 | 129 | LZCY | Tibetan | 21 |  |
| 45 | Han (Jiangxi) | Chinese(Southern Han) | 26 |  | 130 | LZDQ | Tibetan | 9 | 1 |
| 46 | Han (Hubei) | Chinese(Southern Han) | 57 | 4 | 131 | NQAD | Tibetan | 208 | 8 |
| 47 | Han (Hunan) | Chinese(Southern Han) | 38 | 4 | 132 | NQDB | Tibetan | 14 | 1 |
| 48 | Han (Hunan) | Chinese(Southern Han) | 23 | 3 | 133 | NQXB | Tibetan | 5 |  |
| 49 | Han (Sichuan) | Chinese(Southern Han) | 24 | 3 | 134 | RKAR | Tibetan | 34 | 3 |
| 50 | Han (Sichuan) | Chinese(Southern Han) | 38 | 3 | 135 | RKBL | Tibetan | 21 | 1 |
| 51 | Han (congqing) | Chinese(Southern Han) | 43 | 1 | 136 | RKDJ | Tibetan | 38 | 2 |
| 52 | Han (Guizhou) | Chinese(Southern Han) | 11 | 2 | 137 | RKDQ | Tibetan | 154 | 10 |
| 53 | Han (Guizhou) | Chinese(Southern Han) | 107 | 6 | 138 | RKDR | Tibetan | 8 |  |
| 54 | Han (Guizhou) | Chinese(Southern Han) | 10 | 1 | 139 | RKJZ | Tibetan | 19 | 1 |
| 55 | Han (Guizhou) | Chinese(Southern Han) | 18 | 2 | 140 | RKNM | Tibetan | 39 | 2 |
| 56 | Han (Guizhou) | Chinese(Southern Han) | 27 | 2 | 141 | RKSJ | Tibetan | 29 | 3 |
| 57 | Han (Guizhou) | Chinese(Southern Han) | 58 | 3 | 142 | RKXB | Tibetan | 23 |  |
| 58 | Han (Yunnan) | Chinese(Southern Han) | 19 |  | 143 | RKXT | Tibetan | 17 |  |
| 59 | Han (Yunnan) | Chinese(Southern Han) | 14 | 1 | 144 | RKYD | Tibetan | 39 | 2 |
| 60 | Han (Yunnan) | Chinese(Southern Han) | 14 | 2 | 145 | SNCN | Tibetan | 6 |  |
| 61 | Han (Yunnan) | Chinese(Southern Han) | 18 | 1 | 146 | SNDQ | Tibetan | 58 | 2 |
| 62 | Han (Yunnan) | Chinese(Southern Han) | 18 |  | 147 | SNGG | Tibetan | 15 | 2 |
| 63 | Han (Yunnan) | Chinese(Southern Han) | 53 | 3 | 148 | SNLZI | Tibetan | 16 | 3 |
| 64 | Han (Guangxi) | Chinese(Southern Han) | 27 |  | 149 | SNND | Tibetan | 26 | 3 |
| 65 | Han (Guangdong) | Chinese(Southern Han) | 33 | 4 | 150 | SNQS | Tibetan | 13 | 1 |
| 66 | Han (Guangdong) | Chinese(Southern Han) | 31 |  | 151 | SNZL | Tibetan | 8 |  |
| 67 | Hui (Yunnan) | Altaic(North-west China) | 10 | 2 | 152 | QHMQ | Tibetan | 5 |  |
| 68 | Bai (Yunnan) | Tibeto-Burman | 34 | 3 | 153 | Menba | Tibetan | 37 | 1 |
| 69 | Hani (Yunnan) | Tibeto-Burman | 60 | 9 | 154 | Sherpa | Tibetan | 16 | 2 |
| 70 | Jingpo (Yunnan) | Tibeto-Burman | 30 | 3 | 155 | SGRL | Tibetan | 87 | 11 |
| 71 | Lahu (Yunnan) | Tibeto-Burman | 8 |  | 156 | Jarai(Cambodian) | Austronesian | 45 |  |
| 72 | Lisu (Yunnan) | Tibeto-Burman | 10 | 1 | 157 | Tompoun(Cambodian) | Austro-Asiatic | 51 | 1 |
| 73 | Naxi (Yunnan) | Tibeto-Burman | 12 | 5 | 158 | Lao(Cambodian) | Austronesian | 27 |  |
| 74 | Yi (Yunnan) | Tibeto-Burman | 15 | 1 | 159 | Lun(Cambodian) | Austro-Asiatic | 13 |  |
| 75 | Yi (Yunnan) | Tibeto-Burman | 19 | 3 | 160 | Brao(Cambodian) | Austro-Asiatic | 37 |  |
| 76 | Yi (Yunnan) | Tibeto-Burman | 27 | 3 | 161 | Kreung(Cambodian) | Austro-Asiatic | 22 |  |
| 77 | Tujia (Guizhou) | Tibeto-Burman | 33 | 2 | 162 | Kachac(Cambodian) | Austro-Asiatic | 17 |  |
| 78 | Qiang (Sichuan) | Tibeto-Burman | 27 | 9 | 163 | Kravet(Cambodian) | Austro-Asiatic | 24 |  |
| 79 | Yi (Sichuan) | Tibeto-Burman | 24 |  | 164 | Kuy(Cambodian) | Austro-Asiatic | 37 |  |
| 80 | Tujia (Hubei) | Tibeto-Burman | 26 | 3 | 165 | Mel(Cambodian) | Austro-Asiatic | 19 |  |
| 81 | Miao (Yunnan) | Hmong-Mien | 7 |  | 166 | Stieng(Cambodian) | Austro-Asiatic | 12 |  |
| 82 | Yao (Guangdong) | Hmong-Mien | 27 |  | 167 | PHnong(Cambodian) | Austro-Asiatic | 26 |  |
| 83 | Yao (Guangxi) | Hmong-Mien | 10 |  | 168 | KHmer(Cambodian) | Austro-Asiatic | 34 |  |
| 84 | Yao (Guangxi) | Hmong-Mien | 25 | 2 | 169 | Krao(Cambodian) | Austro-Asiatic | 1 |  |
| 85 | Yao (Guangxi) | Hmong-Mien | 21 |  |  |  | **Total** | **6371** | **390** |
