# Supplementary material for: Genetic Evidence of an East Asian Origin and Paleolithic Northward Migration of Y-chromosome Haplogroup N
Source: PLoS One. 2013 Jun 20;8(6):e66102. doi: 10.1371/journal.pone.0066102 (PMC3688714; doi:10.1371/journal.pone.0066102)
Supplement: Table S2 — The STR genotyping data of Hg N samples. (DOCX) [file pone.0066102.s002.docx]

Table S2. The STR genotyping data of Hg N samples.

| Sample | Haplotype | DYS19 | DYS389I | DYS389II | DYS390 | DYS391 | DYS392 | DYS393 |
| --- | --- | --- | --- | --- | --- | --- | --- | --- |
| Han-Henan | N1a | 14 | 13 | 16 | 23 | 10 | 15 | 13 |
| Han-Henan | N1* | 14 | 13 | 15 | 24 | 11 | 15 | 12 |
| Han-Anhui | N1* | 14 | 14 | 15 | 23 | 11 | 15 | 13 |
| Han-Anhui | N1* | 14 | 14 | 17 | 23 | 11 | 14 | 13 |
| Han-Anhui | N1* | 14 | 14 | 17 | 23 | 10 | 14 | 14 |
| Han-Anhui | N1* | 14 | 14 | 17 | 22 | 10 | 14 | 13 |
| Han-Shandong | N1a | 14 | 14 | 16 | 23 | 10 | 15 | 13 |
| Han-Shandong | N1* | 15 | 13 | 16 | 22 | 10 | 14 | 13 |
| Han-Shandong | N1* | 14 | 13 | 16 | 22 | 10 | 14 | 14 |
| Han-Shandong | N1a | 14 | 13 | 16 | 23 | 10 | 14 | 13 |
| Han-Shandong | N1* | 14 | 13 | 15 | 24 | 10 | 16 | 13 |
| Han-Shandong | N1* | 14 | 13 | 16 | 23 | 10 | 14 | 13 |
| Han-Shandong | N1* | 14 | 14 | 16 | 23 | 10 | 14 | 13 |
| Han-Shandong | N1* | 14 | 13 | 15 | 24 | 11 | 15 | 13 |
| Han-Shandong | N1a | 14 | 13 | 16 | 23 | 10 | 15 | 13 |
| Han-shanxi | N1* | 14 | 13 | 15 | 25 | 11 | 15 | 13 |
| Han-shanxi | N1* | 14 | 14 | 16 | 22 | 10 | 14 | 13 |
| Han-shanxi | N1c | 14 | 13 | 12 | 23 | 11 | 14 | 13 |
| Han-shanxi | N1* | 14 | 14 | 15 | 23 | 12 | 15 | 13 |
| Han-shanxi | N1* | 14 | 14 | 16 | 22 | 10 | 14 | 13 |
| Han-shanxi | N1c | 14 | 13 | 15 | 23 | 11 | 14 | 13 |
| Han-shanxi | N1c | 14 | 13 | 16 | 23 | 12 | 14 | 13 |
| Han-shanxi | N1* | 14 | 14 | 17 | 23 | 10 | 15 | 13 |
| Han-shanxi | N1c | 14 | 13 | 16 | 23 | 11 | 14 | 13 |
| Han-shanxi | N1c | 14 | 13 | 16 | 23 | 12 | 14 | 13 |
| Han-shanxi | N1* | 14 | 13 | 15 | 25 | 10 | 15 | 13 |
| Han-Jilin | N1* | 15 | 14 | 16 | 23 | 10 | 14 | 14 |
| Han-Jilin | N1* | 15 | 14 | 17 | 23 | 10 | 14 | 13 |
| Han-Heilongjiang | N1* | 13 | 13 | 15 | 23 | 10 | 15 | 13 |
| Han-Heilongjiang | N1c | 14 | 14 | 16 | 23 | 11 | 16 | 13 |
| Mongolian | N1b | 14 | 13 | 15 | 23 | 10 | 14 | 13 |
| Korean | N1a | 14 | 13 | 16 | 23 | 10 | 15 | 13 |
| Manchuri | N1* | 14 | 13 | 17 | 22 | 10 | 14 | 13 |
| Manchuri | N1c | 14 | 14 | 16 | 23 | 10 | 14 | 14 |
| Manchuri | N1c | 14 | 14 | 17 | 23 | 10 | 14 | 14 |
| Han-Guizhou | N1* | 14 | 14 | 16 | 23 | 10 | 15 | 13 |
| Dong | N* | 14 | 14 | 17 | 23 | 10 | 14 | 14 |
| Bai | N* | 14 | 14 | 17 | 23 | 10 | 14 | 13 |
| Li | N* | 14 | 13 | 16 | 23 | 10 | 14 | 13 |
| She | N1c | 14 | 14 | 16 | 23 | 11 | 14 | 13 |
| Han-Jilin | N1* | 15 | 13 | 15 | 22 | 10 | 15 | 14 |
| Han-Gansu | N1c | 14 | 13 | 16 | 23 | 11 | 16 | 14 |
| Han-Gansu | N1* | 15 | 13 | 16 | 21 | 10 | 15 | 13 |
| Han-Gansu | N1* | 14 | 14 | 18 | 24 | 10 | 14 | 13 |
| Han-Gansu | N1* | 15 | 14 | 16 | 22 | 10 | 14 | 13 |
| Han-Gansu | N1* | 14 | 14 | 16 | 22 | 10 | 14 | 14 |
| Han-Zhejiang | N1* | 14 | 14 | 16 | 23 | 10 | 14 | 14 |
| Han-Zhejiang | N1* | 14 | 14 | 17 | 23 | 10 | 14 | 13 |
| Han-Zhejiang | N1* | 14 | 13 | 17 | 21 | 10 | 15 | 13 |
| Han-Yunnan | N* | 14 | 14 | 16 | 23 | 10 | 14 | 13 |
| Han-Hubei | N1* | 14 | 14 | 18 | 23 | 10 | 14 | 13 |
| Han-Hubei | N* | 14 | 13 | 17 | 23 | 10 | 14 | 13 |
| Han-Hunan | N1* | 14 | 14 | 17 | 22 | 10 | 14 | 13 |
| Hani | N1* | 14 | 14 | 16 | 23 | 11 | 14 | 13 |
| Naxi | N1* | 14 | 14 | 16 | 23 | 11 | 14 | 13 |
| Yi | N1* | 14 | 14 | 17 | 23 | 11 | 14 | 13 |
| Bai | N1c | 15 | 14 | 16 | 23 | 11 | 14 | 13 |
| Naxi | N1* | 14 | 14 | 17 | 23 | 11 | 14 | 13 |
| Lisu | N1* | 14 | 13 | 15 | 25 | 11 | 16 | 13 |
| Hui | N1* | 14 | 14 | 15 | 23 | 10 | 14 | 13 |
| Han-Yunnan | N* | 14 | 14 | 16 | 23 | 10 | 14 | 13 |
| Yi | N1* | 14 | 14 | 17 | 23 | 11 | 14 | 13 |
| Yi | N1c | 15 | 14 | 16 | 23 | 11 | 14 | 13 |
| Yi | N1c | 14 | 14 | 16 | 24 | 11 | 14 | 13 |
| Bai | N1* | 14 | 14 | 17 | 23 | 11 | 14 | 13 |
| Hani | N1* | 14 | 14 | 15 | 23 | 11 | 14 | 13 |
| Han-Yunnan | N1* | 14 | 13 | 16 | 23 | 10 | 14 | 13 |
| Yi | N1* | 14 | 14 | 15 | 23 | 10 | 14 | 13 |
| Hani | N1a | 14 | 13 | 16 | 23 | 10 | 15 | 13 |
| Hui | N1* | 14 | 14 | 17 | 24 | 11 | 14 | 13 |
| Li | N1* | 13 | 13 | 17 | 22 | 10 | 14 | 14 |
| Li | N* | 14 | 14 | 17 | 23 | 11 | 14 | 13 |
| Yao | N1a | 14 | 13 | 16 | 23 | 10 | 15 | 13 |
| Zhuang | N* | 14 | 14 | 17 | 23 | 11 | 14 | 13 |
| Han-Guizhou | N1c | 17 | 15 | 16 | 23 | 10 | 14 | 13 |
| Gelo | N1* | 14 | 14 | 17 | 24 | 10 | 14 | 13 |
| Han-Guizhou | N* | 14 | 14 | 18 | 23 | 10 | 14 | 13 |
| Buyi | N1* | 14 | 14 | 16 | 22 | 10 | 14 | 13 |
| Miao | N1c | 14 | 14 | 16 | 23 | 11 | 14 | 13 |
| Han-Guizhou | N1c | 14 | 14 | 16 | 23 | 11 | 14 | 13 |
| Han-Guizhou | N1* | 14 | 14 | 17 | 23 | 10 | 14 | 13 |
| Han-Hunan | N1* | 14 | 13 | 16 | 24 | 10 | 15 | 13 |
| Naxi | N1* | 14 | 14 | 16 | 23 | 11 | 14 | 13 |
| Dong | N1* | 15 | 14 | 16 | 23 | 11 | 14 | 13 |
| Han-Guizhou | N1c | 14 | 15 | 16 | 23 | 10 | 14 | 13 |
| Han-Guizhou | N1* | 14 | 14 | 14 | 24 | 10 | 14 | 13 |
| Han-Guizhou | N1* | 14 | 13 | 16 | 21 | 11 | 15 | 13 |
| Han-Hunan | N* | 14 | 13 | 17 | 23 | 11 | 14 | 13 |
| Han-Fujian | N1c | 16 | 14 | 16 | 23 | 11 | 14 | 13 |
| Han-Fujian | N1* | 15 | 13 | 17 | 22 | 10 | 15 | 13 |
| Han-Fujian | N1b | 14 | 14 | 17 | 22 | 10 | 14 | 13 |
| Han-Fujian | N1* | 13 | 13 | 17 | 22 | 10 | 14 | 14 |
| Han-Fujian | N1* | 13 | 13 | 17 | 22 | 10 | 14 | 14 |
| Han-Fujian | N1* | 15 | 13 | 16 | 22 | 10 | 15 | 13 |
| Han-Hunan | N* | 13 | 14 | 17 | 23 | 12 | 14 | 13 |
| Tujia | N1* | 14 | 15 | 16 | 23 | 10 | 14 | 13 |
| Tujia | N1* | 15 | 13 | 15 | 24 | 10 | 14 | 13 |
| Han-Hubei | N1c | 16 | 14 | 16 | 23 | 10 | 14 | 13 |
| Dong | N1* | 15 | 13 | 16 | 24 | 10 | 14 | 13 |
| Dong | N1c | 14 | 14 | 16 | 24 | 10 | 16 | 13 |
| Dong | N1* | 17 | 13 | 16 | 24 | 10 | 13 | 13 |
| Han-Hunan | N* | 14 | 13 | 16 | 23 | 11 | 14 | 13 |
| Miao | N* | 14 | 13 | 16 | 23 | 10 | 14 | 13 |
| Han-Hunan | N1* | 14 | 15 | 17 | 24 | 11 | 14 | 13 |
| Han-Guizhou | N* | 15 | 14 | 16 | 23 | 10 | 14 | 13 |
| Gelo | N1* | 14 | 15 | 17 | 22 | 10 | 14 | 14 |
| Tujia | N1* | 15 | 14 | 16 | 23 | 10 | 14 | 13 |
| Tujia | N* | 14 | 13 | 16 | 23 | 11 | 14 | 13 |
| Gelo | N1* | 14 | 14 | 17 | 22 | 10 | 14 | 14 |
| Miao | N1* | 14 | 14 | 17 | 22 | 10 | 14 | 13 |
| Li | N1* | 14 | 14 | 15 | 24 | 11 | 15 | 13 |
| Yao | N1* | 15 | 13 | 16 | 24 | 11 | 14 | 13 |
| Zhuang | N* | 14 | 14 | 16 | 24 | 11 | 14 | 13 |
| Zhuang | N* | 14 | 14 | 18 | 23 | 11 | 14 | 13 |
| Zhuang | N* | 14 | 14 | 16 | 23 | 12 | 14 | 13 |
| Han-Zhejiang | N1c | 14 | 14 | 16 | 23 | 11 | 14 | 13 |
| Tujia | N1* | 14 | 14 | 15 | 23 | 10 | 14 | 13 |
| Han-Hubei | N1* | 14 | 14 | 16 | 23 | 11 | 14 | 14 |
| Han-Guizhou | N1a | 14 | 13 | 16 | 23 | 10 | 15 | 13 |
| Han-Guizhou | N* | 14 | 14 | 18 | 23 | 10 | 14 | 13 |
| Han-Guizhou | N1* | 14 | 13 | 18 | 23 | 10 | 14 | 13 |
| Han-Guizhou | N1* | 14 | 14 | 16 | 24 | 10 | 14 | 13 |
| Han-Guizhou | N1* | 14 | 13 | 15 | 24 | 11 | 14 | 13 |
| Han-Guizhou | N1a | 14 | 13 | 16 | 22 | 10 | 14 | 13 |
| Han-Guizhou | N1a | 14 | 13 | 16 | 23 | 12 | 15 | 13 |
| Han-Yunnan | N* | 14 | 14 | 17 | 23 | 10 | 14 | 13 |
| Naxi | N1* | 14 | 14 | 17 | 23 | 11 | 14 | 13 |
| Han-Hunan | N1a | 14 | 13 | 16 | 23 | 10 | 16 | 13 |
| Han-Sichuan | N1* | 14 | 14 | 16 | 23 | 10 | 14 | 13 |
| Han-Sichuan | N1* | 13 | 13 | 17 | 22 | 10 | 14 | 14 |
| Han-Sichuan | N1c | 14 | 14 | 16 | 23 | 11 | 14 | 13 |
| Han-Chongqing | N1* | 14 | 14 | 17 | 23 | 10 | 14 | 13 |
| Han-Gansu | N1* | 14 | 14 | 16 | 22 | 10 | 14 | 14 |
| Han-Gansu | N1* | 15 | 14 | 17 | 24 | 10 | 14 | 13 |
| Han-Gansu | N1c | 14 | 14 | 16 | 23 | 11 | 14 | 13 |
| Han-Gansu | N1* | 15 | 13 | 16 | 24 | 10 | 14 | 14 |
| Han-Gansu | N1* | 14 | 13 | 17 | 23 | 10 | 14 | 13 |
| Han-Gansu | N* | 14 | 13 | 16 | 24 | 11 | 14 | 13 |
| Han-Gansu | N1a | 14 | 13 | 17 | 23 | 10 | 15 | 14 |
| Han-Sichuan | N1* | 14 | 13 | 15 | 24 | 11 | 15 | 13 |
| Han-Gansu | N1c | 15 | 14 | 16 | 23 | 10 | 14 | 13 |
| Han-Gansu | N1* | 14 | 13 | 17 | 23 | 10 | 14 | 13 |
| Han-Jilin | N1a | 14 | 13 | 16 | 23 | 10 | 15 | 13 |
| Han-Liaoning | N1c | 14 | 14 | 16 | 23 | 11 | 14 | 13 |
| Han-Hunan | N1c | 14 | 14 | 16 | 24 | 11 | 14 | 13 |
| Han-Shandong | N* | 15 | 13 | 16 | 23 | 10 | 15 | 13 |
| Han-Shandong | N1* | 16 | 14 | 14 | 22 | 10 | 14 | 13 |
| Han-Liaoning | N1* | 15 | 14 | 17 | 21 | 11 | 14 | 13 |
| Han-shanxi | N1c | 14 | 14 | 16 | 23 | 10 | 14 | 13 |
| Han-Jilin | N1* | 14 | 14 | 18 | 23 | 10 | 12 | 13 |
| Han-Jilin | N1c | 14 | 14 | 16 | 23 | 10 | 14 | 13 |
| Mongolian | N1* | 14 | 13 | 15 | 23 | 10 | 14 | 14 |
| Uygur | N1c | 14 | 14 | 16 | 24 | 10 | 14 | 13 |
| Uygur | N1c | 14 | 14 | 15 | 23 | 10 | 15 | 13 |
| Han-Zhejiang | N1* | 14 | 13 | 16 | 24 | 10 | 14 | 13 |
| Han-Zhejiang | N1* | 14 | 13 | 15 | 23 | 10 | 14 | 13 |
| Han-Zhejiang | N1* | 14 | 13 | 16 | 24 | 10 | 14 | 13 |
| Han-Zhejiang | N1a | 14 | 13 | 15 | 23 | 10 | 15 | 13 |
| Korean | N1* | 14 | 14 | 16 | 23 | 10 | 14 | 14 |
| Korean | N1b | 14 | 14 | 16 | 23 | 10 | 14 | 14 |
| Korean | N1* | 16 | 14 | 16 | 23 | 10 | 14 | 13 |
| Manchuri | N1* | 14 | 13 | 15 | 23 | 10 | 14 | 13 |
| Manchuri | N1b | 14 | 13 | 16 | 23 | 11 | 15 | 13 |
| Manchuri | N1* | 14 | 13 | 15 | 23 | 10 | 14 | 13 |
| Manchuri | N1c | 14 | 13 | 16 | 23 | 11 | 14 | 13 |
| Manchuri | N1* | 16 | 14 | 17 | 23 | 10 | 15 | 13 |
| Evenk | N1b | 16 | 13 | 16 | 23 | 10 | 14 | 14 |
| Evenk | N1b | 14 | 13 | 16 | 23 | 10 | 14 | 13 |
| Evenk | N1c | 14 | 14 | 16 | 23 | 10 | 14 | 13 |
| Evenk | N1c | 14 | 14 | 16 | 23 | 10 | 14 | 12 |
| Qiang | N1a | 14 | 13 | 16 | 23 | 10 | 15 | 13 |
| Qiang | N1a | 14 | 13 | 16 | 23 | 10 | 15 | 13 |
| Qiang | N1a | 14 | 13 | 16 | 23 | 10 | 15 | 13 |
| Qiang | N1a | 14 | 13 | 16 | 23 | 10 | 15 | 13 |
| Qiang | N1a | 14 | 13 | 16 | 23 | 10 | 15 | 13 |
| Qiang | N1a | 14 | 13 | 16 | 23 | 10 | 15 | 13 |
| Qiang | N1a | 14 | 13 | 16 | 23 | 10 | 15 | 13 |
| Qiang | N1a | 14 | 13 | 16 | 23 | 11 | 15 | 13 |
| Qiang | N1a | 14 | 13 | 16 | 23 | 10 | 15 | 13 |
| Deang | N1* | 14 | 14 | 16 | 23 | 10 | 15 | 13 |
| Deang | N1* | 14 | 14 | 16 | 23 | 10 | 14 | 13 |
| Deang | N1* | 14 | 14 | 16 | 23 | 11 | 14 | 13 |
| Deang | N1* | 14 | 14 | 16 | 23 | 10 | 14 | 13 |
| Deang | N1* | 14 | 14 | 16 | 23 | 11 | 14 | 13 |
| Jingpo | N1* | 14 | 13 | 15 | 22 | 10 | 14 | 13 |
| Jingpo | N1* | 13 | 13 | 15 | 22 | 11 | 14 | 13 |
| Jingpo | N1* | 15 | 14 | 16 | 23 | 11 | 14 | 13 |
| Manchuri | N1* | 14 | 13 | 15 | 23 | 11 | 15 | 13 |
| Manchuri | N1* | 16 | 14 | 17 | 22 | 10 | 16 | 13 |
| Manchuri | N1* | 14 | 13 | 17 | 22 | 10 | 14 | 13 |
| Han-Guangdong | N1* | 16 | 14 | 16 | 20 | 10 | 14 | 14 |
| Han-Guangdong | N1* | 15 | 14 | 16 | 22 | 11 | 14 | 12 |
| Han-Guangdong | N1* | 16 | 14 | 15 | 23 | 11 | 14 | 13 |
| Han-Guangdong | N1* | 14 | 11 | 16 | 23 | 10 | 14 | 13 |
| Han-Sichuan | N1* | 15 | 14 | 18 | 22 | 10 | 14 | 13 |
| Han-Sichuan | N* | 16 | 13 | 16 | 23 | 10 | 13 | 13 |
| Han-Heilongjiang | N1c | 14 | 14 | 16 | 23 | 10 | 14 | 13 |
| Han-Heilongjiang | N1* | 14 | 14 | 16 | 23 | 10 | 14 | 13 |
| Han-Heilongjiang | N1* | 14 | 14 | 15 | 23 | 10 | 14 | 12 |
| Han-Heilongjiang | N1* | 14 | 14 | 15 | 24 | 11 | 14 | 13 |
| Hani | N1* | 15 | 13 | 15 | 23 | 10 | 14 | 13 |
| Hani | N1* | 14 | 13 | 15 | 23 | 10 | 14 | 13 |
| Hani | N1* | 14 | 14 | 16 | 23 | 10 | 14 | 13 |
| Hani | N1* | 14 | 13 | 15 | 23 | 10 | 14 | 13 |
| Hani | N1* | 14 | 14 | 15 | 23 | 10 | 14 | 13 |
| Hani | N1* | 14 | 13 | 15 | 23 | 10 | 14 | 13 |
| uygur | N1* | 14 | 13 | 17 | 23 | 10 | 14 | 13 |
| uygur | N1* | 14 | 15 | 16 | 22 | 10 | 14 | 13 |
| uygur | N* | 14 | 13 | 16 | 24 | 10 | 12 | 13 |
| Hui | N1* | 14 | 13 | 13 | 21 | 9 | 15 | 13 |
| Hui | N* | 14 | 13 | 16 | 23 | 10 | 14 | 13 |
| Hui | N1* | 14 | 15 | 16 | 22 | 10 | 14 | 13 |
| Hui | N1* | 14 | 13 | 17 | 23 | 10 | 14 | 13 |
| Hui | N1b | 14 | 13 | 15 | 23 | 10 | 14 | 13 |
| Manchuri | N1* | 14 | 13 | 15 | 23 | 11 | 15 | 13 |
| Manchuri | N1a | 14 | 14 | 16 | 23 | 10 | 14 | 13 |
| Manchuri | N1a | 14 | 13 | 16 | 23 | 9 | 15 | 13 |
| Cambodia | N1* | 16 | 12 | 17 | 22 | 10 | 15 | 13 |
| Tibetan | N* | 16 | 14 | 17 | 23 | 10 | 14 | 13 |
| Tibetan | N1a | 14 | 14 | 16 | 23 | 10 | 15 | 13 |
| Tibetan | N1a | 14 | 13 | 17 | 22 | 10 | 15 | 13 |
| Tibetan | N1b | 14 | 14 | 16 | 23 | 10 | 14 | 13 |
| Tibetan | N1c | 14 | 13 | 16 | 23 | 11 | 16 | 14 |
| Tibetan | N1c | 14 | 14 | 15 | 24 | 10 | 14 | 13 |
| Tibetan | N1c | 14 | 13 | 16 | 23 | 11 | 16 | 14 |
| Tibetan | N1c | 16 | 12 | 15 | 24 | 10 | 14 | 13 |
| Tibetan | N1c | 16 | 12 | 15 | 24 | 10 | 14 | 13 |
| Tibetan | N1c | 14 | 14 | 16 | 23 | 11 | 16 | 14 |
| Tibetan | N1c | 16 | 12 | 15 | 24 | 10 | 14 | 13 |
| Tibetan | N1c | 14 | 14 | 16 | 23 | 11 | 16 | 14 |
| Tibetan | N1c | 14 | 14 | 16 | 23 | 11 | 16 | 14 |
| Tibetan | N1c | 15 | 12 | 14 | 24 | 10 | 14 | 13 |
| Tibetan | N1* | 15 | 14 | 15 | 24 | 10 | 14 | 13 |
| Tibetan | N1* | 14 | 14 | 17 | 25 | 10 | 15 | 13 |
| Tibetan | N1* | 14 | 14 | 17 | 25 | 10 | 15 | 13 |
| Tibetan | N1* | 14 | 14 | 16 | 24 | 11 | 14 | 13 |
| Tibetan | N1* | 14 | 13 | 15 | 24 | 11 | 14 | 13 |
| Tibetan | N1* | 14 | 13 | 15 | 24 | 11 | 14 | 13 |
| Tibetan | N1* | 15 | 14 | 15 | 22 | 11 | 14 | 13 |
| Tibetan | N1* | 14 | 15 | 16 | 22 | 11 | 14 | 13 |
| Tibetan | N1* | 13 | 14 | 17 | 23 | 11 | 15 | 13 |
| Tibetan | N1* | 15 | 14 | 15 | 24 | 11 | 14 | 12 |
| Tibetan | N1* | 15 | 14 | 15 | 24 | 11 | 14 | 12 |
| Tibetan | N1* | 15 | 14 | 15 | 24 | 11 | 14 | 12 |
| Tibetan | N1* | 15 | 14 | 15 | 24 | 11 | 14 | 12 |
| Tibetan | N1* | 15 | 14 | 15 | 24 | 11 | 14 | 12 |
| Tibetan | N1* | 15 | 14 | 15 | 24 | 11 | 14 | 12 |
| Tibetan | N1* | 14 | 13 | 17 | 25 | 11 | 14 | 13 |
| Tibetan | N1* | 15 | 14 | 15 | 24 | 11 | 14 | 12 |
| Tibetan | N1* | 14 | 14 | 16 | 24 | 10 | 14 | 13 |
| Tibetan | N1* | 15 | 14 | 15 | 24 | 11 | 14 | 12 |
| Tibetan | N1* | 14 | 14 | 17 | 23 | 11 | 14 | 13 |
| Tibetan | N1* | 15 | 13 | 16 | 23 | 10 | 7 | 13 |
| Tibetan | N1* | 14 | 14 | 15 | 22 | 11 | 14 | 13 |
| Tibetan | N1* | 14 | 14 | 17 | 23 | 11 | 14 | 13 |
| Tibetan | N1* | 14 | 14 | 16 | 24 | 11 | 15 | 13 |
| Tibetan | N1* | 14 | 13 | 16 | 23 | 11 | 14 | 13 |
| Tibetan | N1* | 10 | 13 | 16 | 24 | 11 | 14 | 13 |
| Tibetan | N1* | 14 | 14 | 16 | 24 | 11 | 14 | 13 |
| Tibetan | N1* | 14 | 14 | 16 | 24 | 11 | 14 | 13 |
| Tibetan | N1* | 15 | 14 | 17 | 23 | 11 | 14 | 13 |
| Tibetan | N1* | 13 | 14 | 16 | 23 | 10 | 14 | 13 |
| Tibetan | N1* | 14 | 14 | 17 | 23 | 11 | 14 | 13 |
| Tibetan | N1* | 16 | 14 | 17 | 23 | 10 | 15 | 15 |
| Tibetan | N1* | 14 | 14 | 17 | 24 | 10 | 14 | 13 |
| Tibetan | N1* | 14 | 14 | 16 | 24 | 11 | 14 | 13 |
| Tibetan | N1* | 14 | 14 | 16 | 24 | 11 | 14 | 13 |
| Tibetan | N1* | 15 | 13 | 16 | 22 | 10 | 14 | 13 |
| Tibetan | N1* | 14 | 15 | 16 | 24 | 11 | 14 | 13 |
| Tibetan | N1* | 14 | 14 | 17 | 23 | 10 | 14 | 13 |
| Tibetan | N1* | 14 | 14 | 16 | 24 | 10 | 14 | 13 |
| Tibetan | N1* | 14 | 14 | 16 | 23 | 11 | 13 | 13 |
| Tibetan | N1* | 14 | 14 | 17 | 23 | 11 | 14 | 13 |
| Tibetan | N1* | 14 | 14 | 16 | 24 | 12 | 14 | 13 |
| Tibetan | N1* | 15 | 14 | 15 | 24 | 10 | 14 | 13 |
| Tibetan | N1* | 14 | 14 | 15 | 24 | 11 | 14 | 13 |
| Tibetan | N1* | 14 | 14 | 17 | 23 | 11 | 14 | 13 |
| Tibetan | N1* | 14 | 13 | 16 | 25 | 11 | 14 | 13 |
| Tibetan | N1* | 15 | 13 | 18 | 23 | 10 | 14 | 14 |
| Tibetan | N1* | 14 | 15 | 18 | 23 | 11 | 14 | 13 |
| Tibetan | N1* | 14 | 14 | 16 | 24 | 11 | 14 | 13 |
| Tibetan | N1* | 14 | 14 | 16 | 24 | 10 | 14 | 13 |
| Tibetan | N1* | 14 | 14 | 15 | 24 | 10 | 14 | 13 |
| Tibetan | N1* | 14 | 14 | 16 | 24 | 10 | 14 | 13 |
| Tibetan | N1* | 14 | 14 | 16 | 24 | 11 | 14 | 13 |
| Tibetan | N1* | 14 | 14 | 16 | 24 | 10 | 14 | 13 |
| Tibetan | N1* | 14 | 14 | 16 | 24 | 10 | 14 | 13 |
| Tibetan | N1* | 14 | 14 | 18 | 23 | 11 | 14 | 13 |
| Tibetan | N1* | 14 | 14 | 17 | 23 | 11 | 14 | 13 |
| Tibetan | N1* | 14 | 14 | 17 | 23 | 11 | 14 | 13 |
| Tibetan | N1* | 14 | 13 | 15 | 24 | 10 | 14 | 13 |
| Tibetan | N1* | 14 | 14 | 16 | 23 | 11 | 16 | 14 |
| Tibetan | N1* | 14 | 14 | 16 | 24 | 11 | 14 | 13 |
| Tibetan | N1* | 14 | 14 | 17 | 24 | 10 | 14 | 13 |
| Tibetan | N1* | 14 | 14 | 16 | 23 | 10 | 15 | 13 |
| Tibetan | N1* | 14 | 15 | 17 | 24 | 11 | 14 | 13 |
| Tibetan | N1* | 14 | 13 | 16 | 25 | 11 | 14 | 13 |
| Tibetan | N1* | 15 | 13 | 16 | 24 | 10 | 14 | 13 |
| Tibetan | N1* | 14 | 14 | 16 | 25 | 9 | 14 | 13 |
| Tibetan | N1* | 14 | 13 | 18 | 23 | 10 | 15 | 13 |
| Tibetan | N1* | 14 | 14 | 16 | 24 | 10 | 14 | 13 |
| Tibetan | N1* | 14 | 14 | 14 | 24 | 10 | 14 | 13 |
| Tibetan | N1* | 14 | 14 | 16 | 24 | 11 | 14 | 14 |
| Tibetan | N1* | 15 | 14 | 17 | 23 | 11 | 14 | 13 |
| Tibetan | N1* | 14 | 15 | 17 | 23 | 11 | 14 | 13 |
| Tibetan | N1* | 14 | 14 | 18 | 23 | 10 | 14 | 13 |
| Tibetan | N1* | 14 | 14 | 18 | 23 | 10 | 14 | 13 |
| Tibetan | N1* | 14 | 14 | 16 | 23 | 11 | 14 | 14 |
| Tibetan | N1* | 14 | 14 | 17 | 23 | 10 | 14 | 13 |
| Tibetan | N1* | 14 | 14 | 16 | 23 | 10 | 14 | 13 |
| Tibetan | N1* | 13 | 14 | 16 | 23 | 12 | 14 | 13 |
| Tibetan | N1* | 14 | 14 | 17 | 24 | 10 | 15 | 13 |
| Tibetan | N1* | 14 | 14 | 15 | 25 | 10 | 14 | 13 |
| Tibetan | N1* | 14 | 13 | 16 | 23 | 10 | 7 | 13 |
| Tibetan | N1* | 14 | 13 | 16 | 23 | 11 | 7 | 13 |
| Tibetan | N1* | 14 | 14 | 16 | 24 | 11 | 14 | 13 |
| Tibetan | N1* | 15 | 13 | 17 | 23 | 10 | 14 | 13 |
| Tibetan | N1* | 14 | 14 | 15 | 22 | 11 | 14 | 13 |
| Tibetan | N1* | 14 | 15 | 16 | 24 | 10 | 14 | 13 |
| Tibetan | N1* | 16 | 14 | 16 | 23 | 10 | 15 | 13 |
| Tibetan | N1* | 14 | 14 | 15 | 24 | 10 | 15 | 13 |
| Tibetan | N1* | 15 | 13 | 17 | 23 | 10 | 14 | 13 |
| Tibetan | N1* | 14 | 14 | 16 | 24 | 11 | 14 | 14 |
| Tibetan | N1* | 15 | 13 | 17 | 23 | 10 | 14 | 13 |
| Tibetan | N1* | 14 | 14 | 16 | 24 | 10 | 14 | 13 |
| Tibetan | N1* | 14 | 14 | 16 | 24 | 11 | 14 | 13 |
| Tibetan | N1* | 15 | 13 | 16 | 24 | 10 | 14 | 13 |
| Tibetan | N1* | 14 | 14 | 16 | 24 | 10 | 14 | 13 |
| Tibetan | N1* | 14 | 14 | 16 | 22 | 11 | 15 | 13 |
| Tibetan | N1* | 14 | 14 | 17 | 23 | 10 | 14 | 13 |
| Tibetan | N1* | 14 | 13 | 16 | 25 | 11 | 14 | 13 |
| Tibetan | N1* | 14 | 14 | 16 | 24 | 10 | 14 | 13 |
| Tibetan | N1* | 15 | 13 | 16 | 24 | 10 | 14 | 13 |
| Tibetan | N1* | 14 | 13 | 16 | 23 | 11 | 7 | 13 |
| Tibetan | N1* | 14 | 14 | 16 | 24 | 11 | 14 | 13 |
| Tibetan | N1* | 14 | 14 | 16 | 24 | 10 | 14 | 13 |
| Tibetan | N1* | 14 | 13 | 16 | 23 | 10 | 7 | 13 |
| Tibetan | N1* | 14 | 14 | 17 | 23 | 11 | 14 | 13 |
| Tibetan | N1* | 14 | 14 | 17 | 23 | 11 | 14 | 12 |
| Tibetan | N1* | 15 | 14 | 17 | 22 | 9 | 14 | 13 |
| Tibetan | N1* | 14 | 14 | 16 | 23 | 11 | 14 | 13 |
| Tibetan | N1* | 14 | 14 | 16 | 23 | 11 | 14 | 13 |
| Tibetan | N1* | 14 | 14 | 16 | 23 | 11 | 14 | 13 |
| Tibetan | N1* | 15 | 14 | 17 | 23 | 11 | 14 | 13 |
| Tibetan | N1* | 14 | 14 | 16 | 23 | 11 | 14 | 13 |
| Tibetan | N1* | 15 | 13 | 16 | 24 | 11 | 14 | 13 |
| Tibetan | N1* | 14 | 14 | 17 | 23 | 11 | 14 | 13 |
| Tibetan | N1* | 14 | 14 | 16 | 23 | 10 | 16 | 14 |
| Tibetan | N1* | 14 | 14 | 16 | 23 | 11 | 14 | 13 |
| Tibetan | N1* | 15 | 14 | 16 | 24 | 11 | 14 | 13 |
| Tibetan | N1* | 14 | 13 | 16 | 23 | 10 | 14 | 14 |
| Tibetan | N1* | 15 | 14 | 16 | 23 | 11 | 14 | 13 |
| Tibetan | N1* | 14 | 14 | 17 | 23 | 11 | 14 | 14 |
| Tibetan | N1* | 14 | 14 | 15 | 23 | 11 | 14 | 13 |
| Tibetan | N1* | 14 | 13 | 17 | 24 | 11 | 14 | 13 |
| Tibetan | N1* | 14 | 14 | 16 | 23 | 11 | 14 | 13 |
| Tibetan | N1* | 14 | 14 | 15 | 23 | 11 | 14 | 13 |
| Tibetan | N1* | 15 | 13 | 17 | 23 | 11 | 14 | 13 |
| Tibetan | N1* | 14 | 14 | 16 | 24 | 11 | 14 | 13 |
| Tibetan | N1* | 14 | 14 | 15 | 24 | 10 | 15 | 13 |
| Tibetan | N1* | 14 | 14 | 16 | 24 | 11 | 14 | 13 |
| Tibetan | N1* | 15 | 13 | 17 | 23 | 10 | 14 | 13 |
| Japanese01^A^ | N1* | 14 | 13 | 15 | 24 | 10 | 15 | 13 |
| Japanese02 ^A^ | N1* | 15 | 14 | 16 | 23 | 11 | 14 | 13 |
| Japanese03 ^A^ | N1* | 13 | 14 | 16 | 22 | 10 | 14 | 13 |
| Japanese04 ^A^ | N1c | 14 | 14 | 16 | 22 | 10 | 14 | 13 |
| Laos01^B^ | N1* | 15 | 13 | 16 | 22 | 10 | 14 | 13 |
| Laos02 ^B^ | N1c | 14 | 14 | 16 | 23 | 10 | 14 | 13 |
| Laos03 ^B^ | N1c | 14 | 14 | 16 | 23 | 10 | 14 | 13 |
| Laos04 ^B^ | N1c | 14 | 14 | 16 | 23 | 10 | 14 | 13 |
| Chinese-N*^C^ | N* | 14 | 14 | 17 | 23 | 10 | 14 | 13 |
| Chinese-N* ^C^ | N* | 14 | 14 | 16 | 23 | 10 | 13 | 13 |
| Chinese-N* ^C^ | N* | 14 | 14 | 17 | 23 | 10 | 16 | 13 |
| Chinese-N* ^C^ | N* | 14 | 14 | 15 | 23 | 10 | 14 | 13 |
| Fiji-N* ^C^ | N* | 13 | 13 | 17 | 22 | 10 | 14 | 14 |
| Kazah-N1 ^C^ | N1a | 14 | 13 | 16 | 22 | 10 | 15 | 13 |
| Kazah-N1 ^C^ | N1a | 14 | 13 | 16 | 22 | 10 | 15 | 13 |
| Eskomo-N2 ^C^ | N1b | 14 | 13 | 16 | 24 | 10 | 15 | 13 |
| Evenk-N2 ^C^ | N1b | 14 | 13 | 16 | 23 | 10 | 14 | 13 |
| Altaian-N2 ^C^ | N1b | 14 | 13 | 16 | 23 | 10 | 14 | 13 |
| Evenk-N2 ^C^ | N1b | 14 | 13 | 16 | 23 | 10 | 14 | 13 |
| Tuva-N2 ^C^ | N1b | 15 | 13 | 16 | 23 | 11 | 14 | 13 |
| Tuva-N2 ^C^ | N1b | 15 | 13 | 16 | 23 | 11 | 14 | 13 |
| Tuva-N2 ^C^ | N1b | 14 | 13 | 16 | 23 | 10 | 14 | 13 |
| Altaian-N2 ^C^ | N1b | 14 | 13 | 16 | 23 | 10 | 15 | 12 |
| Khakash-N2 ^C^ | N1b | 14 | 13 | 16 | 23 | 10 | 14 | 13 |
| Komi-N2 ^C^ | N1b | 13 | 13 | 18 | 23 | 10 | 12 | 13 |
| Komi-N2 ^C^ | N1b | 13 | 13 | 18 | 23 | 10 | 12 | 12 |
| Tatar-N2 ^C^ | N1b | 13 | 13 | 17 | 23 | 10 | 12 | 13 |
| Vepsa-N2 ^C^ | N1b | 13 | 13 | 18 | 23 | 10 | 11 | 12 |
| Mari-N2 ^C^ | N1b | 13 | 13 | 18 | 23 | 10 | 12 | 13 |
| Russian-N2 ^C^ | N1b | 13 | 13 | 18 | 23 | 10 | 12 | 13 |
| Udmurt-N3 ^C^ | N1c | 14 | 13 | 16 | 23 | 10 | 14 | 13 |
| Komi-N3 ^C^ | N1c | 14 | 14 | 15 | 23 | 11 | 14 | 13 |
| Komi-N3 ^C^ | N1c | 14 | 14 | 15 | 23 | 10 | 14 | 13 |
| Komi-N3 ^C^ | N1c | 14 | 14 | 16 | 23 | 10 | 15 | 13 |
| Komi-N3 ^C^ | N1c | 14 | 14 | 16 | 23 | 11 | 14 | 13 |
| Mari –N3 ^C^ | N1c | 14 | 14 | 16 | 23 | 10 | 14 | 13 |
| Bashkir-N3 ^C^ | N1c | 14 | 13 | 16 | 23 | 11 | 14 | 14 |
| Chuvash-N3 ^C^ | N1c | 14 | 14 | 15 | 23 | 10 | 14 | 13 |
| Ukrainian-N3 ^C^ | N1c | 15 | 14 | 16 | 23 | 11 | 14 | 14 |
| Russian-N3 ^C^ | N1c | 14 | 14 | 16 | 23 | 10 | 14 | 14 |
| Slovak-N3 ^C^ | N1c | 15 | 13 | 16 | 23 | 12 | 14 | 14 |
| Slovak-N3 ^C^ | N1c | 13 | 14 | 15 | 23 | 11 | 14 | 14 |
| Slovak-N3 ^C^ | N1c | 15 | 13 | 16 | 23 | 11 | 14 | 14 |
| Slovak-N3 ^C^ | N1c | 14 | 14 | 16 | 24 | 11 | 14 | 14 |
| Estonian-N3 ^C^ | N1c | 14 | 14 | 16 | 23 | 11 | 14 | 14 |
| Estonian-N3 ^C^ | N1c | 14 | 14 | 16 | 23 | 11 | 14 | 14 |
| Estonian-N3 ^C^ | N1c | 14 | 14 | 17 | 23 | 11 | 14 | 14 |
| Estonian-N3 ^C^ | N1c | 14 | 14 | 17 | 24 | 11 | 14 | 13 |
| Estonian-N3 ^C^ | N1c | 14 | 14 | 17 | 23 | 11 | 14 | 13 |
| Estonian-N3 ^C^ | N1c | 15 | 13 | 16 | 23 | 10 | 15 | 14 |
| Karelian-N3 ^C^ | N1c | 14 | 14 | 16 | 24 | 11 | 14 | 14 |
| Vepsa-N3 ^C^ | N1c | 14 | 14 | 16 | 23 | 10 | 14 | 14 |
| Yakut-N3 ^C^ | N1c | 14 | 14 | 18 | 23 | 11 | 16 | 14 |
| Yakut-N3 ^C^ | N1c | 14 | 14 | 18 | 23 | 11 | 16 | 14 |
| Yakut-N3 ^C^ | N1c | 14 | 14 | 18 | 23 | 11 | 16 | 14 |
| Yakut-N3 ^C^ | N1c | 14 | 15 | 18 | 23 | 11 | 16 | 14 |
| Tuva-N3 ^C^ | N1c | 14 | 14 | 16 | 23 | 10 | 14 | 14 |
| Tuva-N3 ^C^ | N1c | 15 | 14 | 16 | 23 | 11 | 14 | 13 |
| Tuva-N3 ^C^ | N1c | 14 | 14 | 16 | 23 | 11 | 14 | 14 |
| Tuva-N3 ^C^ | N1c | 15 | 13 | 16 | 23 | 11 | 14 | 13 |
| Altaian-N3 ^C^ | N1c | 14 | 14 | 16 | 23 | 10 | 14 | 13 |
| Altaian-N3 ^C^ | N1c | 14 | 14 | 16 | 23 | 11 | 14 | 14 |
| Chukchi-N3 ^C^ | N1c | 14 | 13 | 17 | 23 | 11 | 14 | 13 |
| Chukchi-N3 ^C^ | N1c | 14 | 14 | 16 | 23 | 10 | 14 | 14 |
| Eskimo-N3 ^C^ | N1c | 14 | 14 | 16 | 23 | 11 | 14 | 14 |
| Russian01^D^ | N1b | 13 | 13 | 18 | 23 | 10 | 12 | 13 |
| Russian02 ^D^ | N1b | 13 | 13 | 19 | 23 | 10 | 12 | 12 |
| Russian03 ^D^ | N1b | 13 | 13 | 18 | 24 | 10 | 12 | 13 |
| Russian04 ^D^ | N1b | 13 | 13 | 19 | 23 | 10 | 12 | 12 |
| Russian05 ^D^ | N1b | 13 | 13 | 19 | 23 | 10 | 12 | 12 |
| Russian06 ^D^ | N1b | 13 | 13 | 19 | 23 | 10 | 12 | 12 |
| Russian07 ^D^ | N1b | 13 | 13 | 19 | 23 | 10 | 12 | 12 |
| Russian08 ^D^ | N1b | 13 | 13 | 19 | 23 | 10 | 12 | 12 |
| Russian09 ^D^ | N1b | 13 | 13 | 18 | 24 | 10 | 12 | 13 |
| Russian10 ^D^ | N1b | 13 | 13 | 18 | 23 | 10 | 12 | 12 |
| Russian11 ^D^ | N1b | 13 | 13 | 19 | 23 | 10 | 12 | 12 |
| Russian12 ^D^ | N1b | 13 | 13 | 19 | 23 | 10 | 12 | 12 |
| Russian13 ^D^ | N1b | 13 | 13 | 21 | 23 | 10 | 12 | 12 |
| Russian14 ^D^ | N1b | 13 | 13 | 18 | 24 | 10 | 12 | 13 |
| Russian15 ^D^ | N1b | 13 | 13 | 19 | 23 | 10 | 12 | 12 |
| Russian16 ^D^ | N1b | 14 | 13 | 17 | 23 | 10 | 12 | 13 |
| Russian17 ^D^ | N1b | 13 | 13 | 18 | 23 | 10 | 12 | 13 |
| Russian18 ^D^ | N1b | 13 | 13 | 18 | 23 | 10 | 12 | 13 |
| Russian19 ^D^ | N1b | 13 | 13 | 18 | 23 | 11 | 12 | 13 |
| Russian20 ^D^ | N1b | 13 | 13 | 19 | 23 | 10 | 12 | 12 |
| Russian21 ^D^ | N1b | 13 | 13 | 19 | 23 | 10 | 12 | 12 |
| Russian22 ^D^ | N1b | 13 | 13 | 17 | 23 | 10 | 12 | 13 |
| Russian23 ^D^ | N1b | 14 | 13 | 18 | 23 | 10 | 12 | 12 |
| Russian24 ^D^ | N1b | 13 | 13 | 18 | 23 | 11 | 12 | 12 |
| Russian25 ^D^ | N1b | 14 | 13 | 16 | 23 | 10 | 14 | 13 |
| Russian26 ^D^ | N1b | 14 | 14 | 16 | 24 | 10 | 14 | 13 |
| Russian27 ^D^ | N1b | 14 | 13 | 15 | 23 | 10 | 14 | 13 |
| Russian28 ^D^ | N1b | 14 | 13 | 16 | 24 | 10 | 14 | 13 |
| Evenks03^E^ | N1b | 14 | 13 | 16 | 23 | 10 | 14 | 13 |
| Mongolians02^E^ | N1b | 14 | 13 | 16 | 23 | 10 | 14 | 13 |
| Mongolians03^E^ | N1b | 14 | 13 | 16 | 23 | 10 | 14 | 13 |
| Altainans13^E^ | N1b | 14 | 13 | 16 | 23 | 10 | 14 | 13 |
| Tofalars09 ^E^ | N1b | 14 | 13 | 16 | 23 | 10 | 14 | 13 |
| Tofalars10 ^E^ | N1b | 14 | 13 | 16 | 23 | 10 | 14 | 13 |
| Tofalars11^E^ | N1b | 14 | 13 | 16 | 23 | 10 | 14 | 13 |
| Tofalars12 ^E^ | N1b | 14 | 13 | 16 | 23 | 10 | 14 | 13 |
| Khakassians10^E^ | N1b | 14 | 13 | 16 | 23 | 10 | 14 | 13 |
| Khakassians11^E^ | N1b | 14 | 13 | 16 | 23 | 10 | 14 | 13 |
| Khakassians12^E^ | N1b | 14 | 13 | 16 | 23 | 10 | 14 | 13 |
| Khakassians13^E^ | N1b | 14 | 13 | 16 | 23 | 10 | 14 | 13 |
| Khakassians14^E^ | N1b | 14 | 13 | 16 | 23 | 10 | 14 | 13 |
| Khakassians15^E^ | N1b | 14 | 13 | 16 | 23 | 10 | 14 | 13 |
| Khakassians16^E^ | N1b | 14 | 13 | 16 | 23 | 10 | 14 | 13 |
| Shors02 ^E^ | N1b | 14 | 13 | 16 | 23 | 10 | 14 | 13 |
| Shors03 ^E^ | N1b | 14 | 13 | 16 | 23 | 10 | 14 | 13 |
| Shors04 ^E^ | N1b | 14 | 13 | 16 | 23 | 10 | 14 | 13 |
| Shors05 ^E^ | N1b | 14 | 13 | 16 | 23 | 10 | 14 | 13 |
| Shors06 ^E^ | N1b | 14 | 13 | 16 | 23 | 10 | 14 | 13 |
| Shors07 ^E^ | N1b | 14 | 13 | 16 | 23 | 10 | 14 | 13 |
| Buryats42 ^E^ | N1b | 14 | 13 | 16 | 22 | 10 | 14 | 13 |
| Evenks04 ^E^ | N1b | 14 | 13 | 16 | 23 | 10 | 14 | 13 |
| Evenks05 ^E^ | N1b | 14 | 13 | 16 | 23 | 10 | 14 | 13 |
| Evenks06 ^E^ | N1b | 14 | 13 | 16 | 23 | 10 | 14 | 13 |
| Evenks07 ^E^ | N1b | 14 | 13 | 16 | 23 | 10 | 14 | 13 |
| Evenks08 ^E^ | N1b | 14 | 13 | 16 | 23 | 10 | 14 | 13 |
| Evenks09 ^E^ | N1b | 14 | 13 | 16 | 23 | 10 | 14 | 13 |
| Evenks10 ^E^ | N1b | 14 | 13 | 16 | 23 | 10 | 14 | 13 |
| Evenks11 ^E^ | N1b | 14 | 13 | 16 | 23 | 10 | 14 | 13 |
| Tofalars13 ^E^ | N1b | 14 | 13 | 16 | 23 | 10 | 14 | 13 |
| Tofalars14 ^E^ | N1b | 14 | 13 | 16 | 23 | 10 | 14 | 13 |
| Tofalars15 ^E^ | N1b | 14 | 13 | 16 | 23 | 10 | 14 | 13 |
| Khakassians17 ^E^ | N1b | 14 | 13 | 16 | 23 | 10 | 14 | 13 |
| Khakassians18 ^E^ | N1b | 14 | 13 | 16 | 23 | 10 | 14 | 13 |
| Khakassians19 ^E^ | N1b | 14 | 13 | 16 | 23 | 10 | 14 | 13 |
| Khakassians20 ^E^ | N1b | 14 | 13 | 16 | 23 | 10 | 14 | 13 |
| Khakassians21 ^E^ | N1b | 14 | 13 | 16 | 23 | 10 | 14 | 13 |
| Khakassians22 ^E^ | N1b | 14 | 13 | 16 | 23 | 10 | 14 | 13 |
| Khakassians23 ^E^ | N1b | 14 | 13 | 16 | 23 | 10 | 14 | 13 |
| Evenks12 ^E^ | N1b | 14 | 13 | 16 | 23 | 10 | 14 | 13 |
| Khakassians24 ^E^ | N1b | 14 | 13 | 16 | 23 | 10 | 14 | 13 |
| Khakassians25 ^E^ | N1b | 14 | 13 | 16 | 23 | 10 | 14 | 13 |
| Khakassians26 ^E^ | N1b | 14 | 13 | 15 | 23 | 10 | 15 | 13 |
| Khakassians27 ^E^ | N1b | 14 | 13 | 17 | 22 | 10 | 14 | 13 |
| Khakassians28 ^E^ | N1b | 14 | 13 | 17 | 24 | 10 | 14 | 13 |
| Khakassians29 ^E^ | N1b | 14 | 13 | 16 | 23 | 10 | 14 | 13 |
| Khakassians30 ^E^ | N1b | 14 | 13 | 17 | 23 | 10 | 14 | 13 |
| Kalmyks01 ^E^ | N1b | 14 | 13 | 15 | 23 | 10 | 14 | 13 |
| Mongolians04 ^E^ | N1b | 14 | 13 | 16 | 24 | 10 | 14 | 13 |
| Tuvinians16 ^E^ | N1b | 14 | 13 | 16 | 24 | 10 | 14 | 13 |
| Khakassians31 ^E^ | N1b | 14 | 13 | 16 | 23 | 11 | 14 | 13 |
| Tuvinians17 ^E^ | N1b | 15 | 13 | 16 | 23 | 11 | 14 | 13 |
| Tuvinians18 ^E^ | N1b | 15 | 13 | 16 | 23 | 11 | 14 | 13 |
| Tuvinians19 ^E^ | N1b | 15 | 13 | 16 | 23 | 11 | 14 | 13 |
| Tuvinians20 ^E^ | N1b | 15 | 13 | 16 | 23 | 11 | 14 | 13 |
| Tuvinians21 ^E^ | N1b | 15 | 13 | 16 | 23 | 11 | 14 | 13 |
| Tuvinians22 ^E^ | N1b | 15 | 13 | 16 | 23 | 11 | 14 | 13 |
| Tuvinians23 ^E^ | N1b | 15 | 13 | 16 | 23 | 11 | 14 | 13 |
| Tuvinians24 ^E^ | N1b | 15 | 13 | 16 | 23 | 11 | 14 | 13 |
| Tuvinians25 ^E^ | N1b | 15 | 13 | 16 | 23 | 11 | 14 | 13 |
| Tuvinians26 ^E^ | N1b | 15 | 13 | 16 | 23 | 11 | 14 | 13 |
| Tuvinians27 ^E^ | N1b | 15 | 13 | 16 | 23 | 11 | 14 | 13 |
| Tuvinians28 ^E^ | N1b | 15 | 13 | 16 | 23 | 11 | 14 | 13 |
| Tuvinians29 ^E^ | N1b | 15 | 13 | 16 | 23 | 11 | 14 | 13 |
| Tuvinians30 ^E^ | N1b | 15 | 13 | 16 | 23 | 11 | 14 | 13 |
| Tuvinians31 ^E^ | N1b | 15 | 13 | 16 | 23 | 11 | 14 | 13 |
| Kalmyks02 ^E^ | N1b | 15 | 13 | 16 | 23 | 11 | 14 | 13 |
| Tuvinians32 ^E^ | N1b | 15 | 13 | 16 | 23 | 11 | 14 | 14 |
| Tuvinians33 ^E^ | N1b | 15 | 13 | 16 | 23 | 10 | 14 | 13 |
| Tuvinians34 ^E^ | N1b | 15 | 13 | 16 | 23 | 11 | 14 | 13 |
| Tuvinians35 ^E^ | N1b | 15 | 13 | 16 | 23 | 11 | 14 | 13 |
| Tofalars16 ^E^ | N1b | 15 | 13 | 16 | 23 | 11 | 14 | 13 |
| Tofalars17 ^E^ | N1b | 15 | 13 | 16 | 23 | 11 | 14 | 13 |
| Tofalars18 ^E^ | N1b | 15 | 13 | 16 | 23 | 11 | 14 | 13 |
| Tofalars19 ^E^ | N1b | 15 | 13 | 16 | 23 | 11 | 14 | 13 |
| Tofalars20 ^E^ | N1b | 15 | 13 | 16 | 23 | 11 | 14 | 13 |
| Tofalars21 ^E^ | N1b | 15 | 13 | 16 | 23 | 11 | 14 | 13 |
| Tuvinians36 ^E^ | N1b | 15 | 13 | 16 | 22 | 11 | 14 | 13 |
| Tuvinians37 ^E^ | N1b | 15 | 12 | 16 | 23 | 11 | 14 | 13 |
| Tuvinians38 ^E^ | N1b | 15 | 13 | 17 | 23 | 11 | 14 | 13 |
| Tuvinians39 ^E^ | N1b | 15 | 13 | 17 | 23 | 11 | 14 | 13 |
| Tuvinians40 ^E^ | N1b | 15 | 13 | 16 | 23 | 11 | 15 | 13 |
| Russians47 ^E^ | N1b | 13 | 13 | 17 | 23 | 10 | 12 | 13 |
| Russian01 ^E^ | N1c | 14 | 13 | 16 | 23 | 11 | 14 | 13 |
| Russian02 ^E^ | N1c | 15 | 13 | 16 | 23 | 11 | 14 | 14 |
| Russian03 ^E^ | N1c | 15 | 13 | 16 | 23 | 11 | 14 | 14 |
| Russian04 ^E^ | N1c | 15 | 13 | 16 | 23 | 11 | 14 | 14 |
| Russian05 ^E^ | N1c | 15 | 13 | 16 | 23 | 11 | 14 | 14 |
| Russian06 ^E^ | N1c | 15 | 13 | 16 | 23 | 11 | 14 | 14 |
| Russian07 ^E^ | N1c | 14 | 14 | 17 | 24 | 11 | 14 | 14 |
| Russian08 ^E^ | N1c | 15 | 13 | 16 | 23 | 10 | 14 | 14 |
| Russian09 ^E^ | N1c | 15 | 13 | 16 | 23 | 10 | 14 | 14 |
| Russian10 ^E^ | N1c | 14 | 14 | 16 | 23 | 11 | 14 | 14 |
| Russian11 ^E^ | N1c | 14 | 14 | 16 | 23 | 11 | 14 | 14 |
| Russian12 ^E^ | N1c | 14 | 14 | 16 | 23 | 11 | 14 | 14 |
| Russian13 ^E^ | N1c | 14 | 14 | 16 | 23 | 11 | 14 | 14 |
| Russian14 ^E^ | N1c | 14 | 14 | 16 | 23 | 11 | 14 | 14 |
| Russian15 ^E^ | N1c | 14 | 14 | 16 | 23 | 11 | 14 | 14 |
| Tuvinians01 ^E^ | N1c | 14 | 14 | 16 | 23 | 11 | 14 | 14 |
| Tuvinians02 ^E^ | N1c | 14 | 14 | 16 | 23 | 11 | 14 | 14 |
| Tuvinians03 ^E^ | N1c | 14 | 14 | 16 | 23 | 11 | 14 | 14 |
| Tuvinians04 ^E^ | N1c | 14 | 14 | 16 | 23 | 11 | 14 | 14 |
| Tuvinians05 ^E^ | N1c | 14 | 14 | 16 | 23 | 11 | 14 | 14 |
| Tuvinians06 ^E^ | N1c | 14 | 14 | 16 | 23 | 11 | 14 | 14 |
| Sojots01 ^E^ | N1c | 14 | 14 | 16 | 23 | 11 | 14 | 14 |
| Sojots02 ^E^ | N1c | 14 | 14 | 16 | 23 | 11 | 14 | 14 |
| Tofalars01 ^E^ | N1c | 14 | 14 | 16 | 23 | 11 | 14 | 14 |
| Tofalars02 ^E^ | N1c | 14 | 14 | 16 | 23 | 11 | 14 | 14 |
| Tofalars03 ^E^ | N1c | 14 | 14 | 16 | 23 | 11 | 14 | 14 |
| Tofalars04 ^E^ | N1c | 14 | 14 | 16 | 23 | 11 | 14 | 14 |
| Tofalars05 ^E^ | N1c | 14 | 14 | 16 | 23 | 11 | 14 | 14 |
| Tofalars06 ^E^ | N1c | 14 | 14 | 16 | 23 | 11 | 14 | 14 |
| Tofalars07 ^E^ | N1c | 14 | 14 | 16 | 23 | 11 | 14 | 14 |
| Russian16 ^E^ | N1c | 15 | 14 | 16 | 22 | 11 | 14 | 14 |
| Russian17 ^E^ | N1c | 14 | 13 | 16 | 23 | 11 | 14 | 14 |
| Russian18 ^E^ | N1c | 14 | 14 | 16 | 25 | 11 | 14 | 14 |
| Russian19 ^E^ | N1c | 14 | 14 | 16 | 25 | 11 | 14 | 14 |
| Russian20 ^E^ | N1c | 14 | 14 | 16 | 24 | 11 | 14 | 14 |
| Evenks01 ^E^ | N1c | 14 | 14 | 16 | 24 | 11 | 14 | 14 |
| Sojots03 ^E^ | N1c | 14 | 14 | 16 | 24 | 11 | 14 | 14 |
| Russian21 ^E^ | N1c | 14 | 14 | 16 | 23 | 11 | 14 | 14 |
| Mongolians01 ^E^ | N1c | 14 | 14 | 16 | 23 | 11 | 14 | 14 |
| Russian22 ^E^ | N1c | 14 | 13 | 16 | 23 | 11 | 13 | 14 |
| Russian23 ^E^ | N1c | 14 | 14 | 18 | 23 | 11 | 16 | 14 |
| Yakuts01 ^E^ | N1c | 14 | 14 | 18 | 23 | 11 | 16 | 14 |
| Yakuts02 ^E^ | N1c | 14 | 14 | 18 | 23 | 11 | 16 | 14 |
| Russian24 ^E^ | N1c | 16 | 14 | 16 | 23 | 11 | 14 | 14 |
| Russian25 ^E^ | N1c | 14 | 12 | 16 | 23 | 11 | 14 | 14 |
| Russian26 ^E^ | N1c | 14 | 14 | 16 | 24 | 11 | 16 | 14 |
| Russian27 ^E^ | N1c | 14 | 13 | 16 | 23 | 10 | 14 | 14 |
| Russian28 ^E^ | N1c | 14 | 14 | 16 | 23 | 12 | 14 | 14 |
| Russian29 ^E^ | N1c | 15 | 14 | 16 | 23 | 11 | 15 | 14 |
| Russian30 ^E^ | N1c | 14 | 13 | 16 | 23 | 11 | 14 | 14 |
| Russian31 ^E^ | N1c | 14 | 12 | 16 | 22 | 11 | 13 | 14 |
| Russian32 ^E^ | N1c | 14 | 12 | 16 | 22 | 11 | 13 | 14 |
| Russian33 ^E^ | N1c | 14 | 14 | 16 | 23 | 11 | 14 | 14 |
| Russian34 ^E^ | N1c | 14 | 14 | 16 | 23 | 11 | 14 | 14 |
| Russian35 ^E^ | N1c | 15 | 14 | 16 | 23 | 11 | 14 | 14 |
| Russian36 ^E^ | N1c | 15 | 14 | 16 | 23 | 11 | 14 | 14 |
| Russian37 ^E^ | N1c | 15 | 13 | 16 | 23 | 11 | 14 | 14 |
| Khakassians01 ^E^ | N1c | 14 | 14 | 16 | 23 | 11 | 14 | 14 |
| Tuvinians07 ^E^ | N1c | 14 | 14 | 16 | 23 | 11 | 14 | 14 |
| Tuvinians08 ^E^ | N1c | 14 | 14 | 16 | 23 | 11 | 14 | 14 |
| Tuvinians09 ^E^ | N1c | 14 | 14 | 16 | 23 | 11 | 14 | 14 |
| Altainans01 ^E^ | N1c | 14 | 14 | 16 | 23 | 11 | 14 | 13 |
| Tuvinians10 ^E^ | N1c | 14 | 14 | 16 | 23 | 11 | 14 | 14 |
| Koryaks01 ^E^ | N1c | 14 | 14 | 14 | 23 | 11 | 14 | 14 |
| Koryaks02 ^E^ | N1c | 14 | 14 | 14 | 23 | 11 | 14 | 14 |
| Koryaks03 ^E^ | N1c | 14 | 14 | 16 | 23 | 11 | 14 | 14 |
| Koryaks04 ^E^ | N1c | 14 | 14 | 16 | 23 | 11 | 14 | 14 |
| Tofalars08 ^E^ | N1c | 14 | 14 | 16 | 23 | 11 | 15 | 14 |
| Yakuts03 ^E^ | N1c | 14 | 14 | 17 | 23 | 11 | 15 | 14 |
| Yakuts04 ^E^ | N1c | 14 | 14 | 17 | 23 | 11 | 15 | 14 |
| Yakuts05 ^E^ | N1c | 14 | 14 | 17 | 23 | 11 | 15 | 14 |
| Yakuts06 ^E^ | N1c | 14 | 14 | 17 | 23 | 10 | 16 | 14 |
| Evenks02 ^E^ | N1c | 14 | 14 | 16 | 23 | 11 | 16 | 14 |
| Yakuts07 ^E^ | N1c | 14 | 14 | 18 | 24 | 11 | 16 | 14 |
| Yakuts08 ^E^ | N1c | 14 | 14 | 18 | 23 | 11 | 17 | 14 |
| Evens01 ^E^ | N1c | 15 | 13 | 16 | 23 | 11 | 14 | 14 |
| Tuvinians11 ^E^ | N1c | 15 | 14 | 16 | 23 | 11 | 14 | 13 |
| Tuvinians12 ^E^ | N1c | 15 | 14 | 16 | 23 | 11 | 14 | 13 |
| Khakassians02 ^E^ | N1c | 15 | 14 | 16 | 23 | 11 | 14 | 13 |
| Khakassians03 ^E^ | N1c | 15 | 14 | 16 | 23 | 11 | 14 | 13 |
| Khakassians04 ^E^ | N1c | 15 | 14 | 16 | 23 | 11 | 14 | 13 |
| Khakassians05 ^E^ | N1c | 15 | 14 | 16 | 23 | 11 | 14 | 13 |
| Khakassians06 ^E^ | N1c | 15 | 14 | 16 | 23 | 11 | 14 | 13 |
| Khakassians07 ^E^ | N1c | 15 | 14 | 16 | 23 | 11 | 14 | 13 |
| Shors01 ^E^ | N1c | 15 | 14 | 16 | 23 | 11 | 14 | 13 |
| Khakassians08 ^E^ | N1c | 16 | 14 | 16 | 23 | 10 | 14 | 13 |
| Khakassians09 ^E^ | N1c | 16 | 13 | 16 | 23 | 11 | 14 | 13 |
| Khakassians10 ^E^ | N1c | 14 | 13 | 16 | 23 | 10 | 14 | 13 |
| Russian38 ^E^ | N1c | 14 | 13 | 16 | 23 | 10 | 13 | 13 |
| Russian39 ^E^ | N1c | 14 | 14 | 16 | 23 | 10 | 14 | 12 |
| Russian40 ^E^ | N1c | 15 | 14 | 18 | 23 | 10 | 14 | 14 |
| Russian41 ^E^ | N1c | 14 | 15 | 16 | 23 | 10 | 14 | 14 |
| Russian42 ^E^ | N1c | 14 | 14 | 16 | 23 | 10 | 14 | 14 |
| Russian43 ^E^ | N1c | 14 | 14 | 16 | 23 | 10 | 14 | 14 |
| Tuvinians13 ^E^ | N1c | 14 | 14 | 16 | 23 | 10 | 14 | 14 |
| Tuvinians14 ^E^ | N1c | 14 | 14 | 16 | 23 | 10 | 14 | 14 |
| Buryats01 ^E^ | N1c | 14 | 14 | 16 | 23 | 10 | 14 | 14 |
| Buryats02 ^E^ | N1c | 14 | 14 | 16 | 23 | 10 | 14 | 14 |
| Buryats03 ^E^ | N1c | 14 | 14 | 16 | 23 | 10 | 14 | 14 |
| Buryats04 ^E^ | N1c | 14 | 14 | 16 | 23 | 10 | 14 | 14 |
| Buryats05 ^E^ | N1c | 14 | 14 | 16 | 23 | 10 | 14 | 14 |
| Buryats06 ^E^ | N1c | 14 | 14 | 16 | 23 | 10 | 14 | 14 |
| Buryats07 ^E^ | N1c | 14 | 14 | 16 | 23 | 10 | 14 | 14 |
| Buryats08 ^E^ | N1c | 14 | 14 | 16 | 23 | 10 | 14 | 14 |
| Buryats09 ^E^ | N1c | 14 | 14 | 16 | 23 | 10 | 14 | 14 |
| Buryats10 ^E^ | N1c | 14 | 14 | 16 | 23 | 10 | 14 | 14 |
| Buryats11 ^E^ | N1c | 14 | 14 | 16 | 23 | 10 | 14 | 14 |
| Buryats12 ^E^ | N1c | 14 | 14 | 16 | 23 | 10 | 14 | 14 |
| Buryats13 ^E^ | N1c | 14 | 14 | 16 | 23 | 10 | 14 | 14 |
| Buryats14 ^E^ | N1c | 14 | 14 | 16 | 23 | 10 | 14 | 14 |
| Buryats15 ^E^ | N1c | 14 | 14 | 16 | 23 | 10 | 14 | 14 |
| Buryats16 ^E^ | N1c | 14 | 14 | 16 | 23 | 10 | 14 | 14 |
| Buryats17 ^E^ | N1c | 14 | 14 | 16 | 23 | 10 | 14 | 14 |
| Buryats18 ^E^ | N1c | 14 | 14 | 16 | 23 | 10 | 14 | 14 |
| Buryats19 ^E^ | N1c | 14 | 14 | 16 | 23 | 10 | 14 | 14 |
| Buryats20 ^E^ | N1c | 14 | 14 | 16 | 23 | 10 | 14 | 14 |
| Buryats21 ^E^ | N1c | 14 | 14 | 16 | 23 | 10 | 14 | 14 |
| Buryats22 ^E^ | N1c | 14 | 14 | 16 | 23 | 10 | 14 | 14 |
| Buryats23 ^E^ | N1c | 14 | 14 | 16 | 23 | 10 | 14 | 14 |
| Buryats24 ^E^ | N1c | 14 | 14 | 16 | 23 | 10 | 14 | 14 |
| Buryats25 ^E^ | N1c | 14 | 14 | 16 | 23 | 10 | 14 | 14 |
| Buryats26 ^E^ | N1c | 14 | 14 | 16 | 23 | 10 | 14 | 14 |
| Buryats27 ^E^ | N1c | 14 | 14 | 16 | 23 | 10 | 14 | 14 |
| Buryats28 ^E^ | N1c | 14 | 14 | 16 | 23 | 10 | 14 | 14 |
| Buryats29 ^E^ | N1c | 14 | 14 | 16 | 23 | 10 | 14 | 14 |
| Buryats30 ^E^ | N1c | 14 | 14 | 16 | 23 | 10 | 14 | 14 |
| Buryats31 ^E^ | N1c | 14 | 14 | 16 | 23 | 10 | 14 | 14 |
| Russian44 ^E^ | N1c | 14 | 14 | 16 | 24 | 10 | 14 | 14 |
| Russian45 ^E^ | N1c | 14 | 14 | 16 | 24 | 10 | 14 | 14 |
| Russian46 ^E^ | N1c | 14 | 14 | 16 | 24 | 10 | 14 | 13 |
| Buryats32 ^E^ | N1c | 14 | 14 | 16 | 23 | 10 | 13 | 14 |
| Buryats33 ^E^ | N1c | 14 | 14 | 16 | 23 | 10 | 14 | 13 |
| Buryats34 ^E^ | N1c | 14 | 14 | 16 | 23 | 10 | 14 | 13 |
| Buryats35 ^E^ | N1c | 14 | 14 | 15 | 23 | 10 | 14 | 14 |
| Buryats36 ^E^ | N1c | 14 | 14 | 15 | 23 | 10 | 14 | 14 |
| Buryats37 ^E^ | N1c | 14 | 14 | 17 | 23 | 10 | 14 | 14 |
| Buryats38 ^E^ | N1c | 14 | 14 | 17 | 23 | 10 | 14 | 14 |
| Altainans02 ^E^ | N1c | 14 | 13 | 18 | 23 | 10 | 14 | 14 |
| Altainans03 ^E^ | N1c | 14 | 13 | 18 | 23 | 10 | 14 | 14 |
| Altainans04 ^E^ | N1c | 14 | 13 | 18 | 23 | 10 | 14 | 14 |
| Altainans05 ^E^ | N1c | 14 | 13 | 18 | 23 | 10 | 14 | 14 |
| Altainans06 ^E^ | N1c | 14 | 13 | 18 | 23 | 10 | 14 | 14 |
| Altainans07 ^E^ | N1c | 14 | 13 | 18 | 23 | 10 | 14 | 14 |
| Altainans08 ^E^ | N1c | 14 | 13 | 18 | 23 | 10 | 14 | 14 |
| Altainans09 ^E^ | N1c | 14 | 13 | 18 | 23 | 10 | 14 | 14 |
| Altainans10 ^E^ | N1c | 14 | 13 | 18 | 23 | 10 | 14 | 14 |
| Altainans11 ^E^ | N1c | 14 | 13 | 18 | 23 | 10 | 14 | 14 |
| Buryats39 ^E^ | N1c | 14 | 14 | 16 | 23 | 10 | 14 | 15 |
| Altainans12 ^E^ | N1c | 14 | 14 | 16 | 23 | 10 | 14 | 13 |
| Buryats40 ^E^ | N1c | 14 | 13 | 16 | 23 | 10 | 14 | 13 |
| Buryats41 ^E^ | N1c | 15 | 14 | 16 | 23 | 10 | 14 | 14 |
| Tuvinians15 ^E^ | N1c | 15 | 14 | 16 | 23 | 10 | 13 | 14 |
| S Karelia01^F^ | N1c | 14 | 13 | 16 | 25 | 10 | 14 | 14 |
| S Karelia02 | N1c | 14 | 14 | 16 | 24 | 11 | 14 | 14 |
| S Karelia03 ^F^ | N1c | 14 | 14 | 16 | 24 | 11 | 14 | 14 |
| S Karelia04 ^F^ | N1c | 14 | 14 | 16 | 24 | 11 | 14 | 14 |
| S Karelia05 ^F^ | N1c | 14 | 14 | 16 | 24 | 11 | 14 | 14 |
| S Karelia06 ^F^ | N1c | 14 | 14 | 16 | 24 | 11 | 14 | 14 |
| S Karelia07 ^F^ | N1c | 14 | 14 | 16 | 24 | 11 | 14 | 14 |
| S Karelia08 ^F^ | N1c | 14 | 14 | 16 | 24 | 11 | 14 | 14 |
| S Karelia09 ^F^ | N1c | 14 | 14 | 16 | 24 | 11 | 14 | 14 |
| S Karelia10 ^F^ | N1c | 14 | 14 | 16 | 24 | 11 | 14 | 14 |
| S Karelia11 ^F^ | N1c | 14 | 14 | 16 | 24 | 11 | 14 | 14 |
| N Karelia01 ^F^ | N1c | 14 | 14 | 16 | 24 | 11 | 14 | 14 |
| N Karelia02 ^F^ | N1c | 14 | 14 | 16 | 24 | 11 | 14 | 14 |
| N Karelia03 ^F^ | N1c | 14 | 14 | 16 | 24 | 11 | 14 | 14 |
| N Savo01 ^F^ | N1c | 14 | 14 | 16 | 24 | 11 | 14 | 14 |
| N Savo02 ^F^ | N1c | 14 | 14 | 16 | 24 | 11 | 14 | 14 |
| N Savo03 ^F^ | N1c | 14 | 14 | 16 | 24 | 11 | 14 | 14 |
| N Savo04 ^F^ | N1c | 14 | 14 | 16 | 24 | 11 | 14 | 14 |
| N Savo05 ^F^ | N1c | 14 | 14 | 16 | 24 | 11 | 14 | 14 |
| N Savo06 ^F^ | N1c | 14 | 14 | 16 | 24 | 11 | 14 | 14 |
| N Savo07 ^F^ | N1c | 14 | 14 | 16 | 24 | 11 | 14 | 14 |
| N Savo08 ^F^ | N1c | 14 | 14 | 16 | 24 | 11 | 14 | 14 |
| N Savo09 ^F^ | N1c | 14 | 14 | 16 | 24 | 11 | 14 | 14 |
| N Savo10 ^F^ | N1c | 14 | 14 | 16 | 24 | 11 | 14 | 14 |
| N Savo11 ^F^ | N1c | 14 | 14 | 16 | 24 | 11 | 14 | 14 |
| N Ostrobothnia01 ^F^ | N1c | 14 | 14 | 16 | 24 | 11 | 14 | 14 |
| N Ostrobothnia02 ^F^ | N1c | 14 | 14 | 16 | 24 | 11 | 14 | 14 |
| N Ostrobothnia03 ^F^ | N1c | 14 | 14 | 16 | 24 | 11 | 14 | 14 |
| N Ostrobothnia04 ^F^ | N1c | 14 | 14 | 16 | 24 | 11 | 14 | 14 |
| N Ostrobothnia05 ^F^ | N1c | 14 | 14 | 16 | 24 | 11 | 14 | 14 |
| N Ostrobothnia06 ^F^ | N1c | 14 | 14 | 16 | 24 | 11 | 14 | 14 |
| N Ostrobothnia07 ^F^ | N1c | 14 | 14 | 16 | 24 | 11 | 14 | 14 |
| N Ostrobothnia08 ^F^ | N1c | 14 | 14 | 16 | 24 | 11 | 14 | 14 |
| N Ostrobothnia09 ^F^ | N1c | 14 | 14 | 16 | 24 | 11 | 14 | 14 |
| N Ostrobothnia10 ^F^ | N1c | 14 | 14 | 16 | 24 | 11 | 14 | 14 |
| N Ostrobothnia11 ^F^ | N1c | 14 | 14 | 16 | 24 | 11 | 14 | 14 |
| N Ostrobothnia12 ^F^ | N1c | 14 | 14 | 16 | 24 | 11 | 14 | 14 |
| N Ostrobothnia13 ^F^ | N1c | 14 | 14 | 16 | 24 | 11 | 14 | 14 |
| N Ostrobothnia14 ^F^ | N1c | 14 | 14 | 16 | 24 | 11 | 14 | 14 |
| N Ostrobothnia15 ^F^ | N1c | 14 | 14 | 16 | 24 | 11 | 14 | 14 |
| N Ostrobothnia16 ^F^ | N1c | 14 | 14 | 16 | 24 | 11 | 14 | 14 |
| N Ostrobothnia17 ^F^ | N1c | 14 | 14 | 16 | 24 | 11 | 14 | 14 |
| N Ostrobothnia18 ^F^ | N1c | 14 | 14 | 16 | 24 | 11 | 14 | 14 |
| N Ostrobothnia19 ^F^ | N1c | 14 | 14 | 16 | 24 | 11 | 14 | 14 |
| N Ostrobothnia20 ^F^ | N1c | 14 | 14 | 16 | 24 | 11 | 14 | 14 |
| N Ostrobothnia21 ^F^ | N1c | 14 | 14 | 16 | 24 | 11 | 14 | 14 |
| N Ostrobothnia22 ^F^ | N1c | 14 | 14 | 16 | 24 | 11 | 14 | 14 |
| N Ostrobothnia23 ^F^ | N1c | 14 | 14 | 16 | 24 | 11 | 14 | 14 |
| N Ostrobothnia24 ^F^ | N1c | 14 | 14 | 16 | 24 | 11 | 14 | 14 |
| N Ostrobothnia25 ^F^ | N1c | 14 | 14 | 16 | 24 | 11 | 14 | 14 |
| N Ostrobothnia26 ^F^ | N1c | 14 | 14 | 16 | 24 | 11 | 14 | 14 |
| N Ostrobothnia27 ^F^ | N1c | 14 | 14 | 16 | 24 | 11 | 14 | 14 |
| N Ostrobothnia28 ^F^ | N1c | 14 | 14 | 16 | 24 | 11 | 14 | 14 |
| N Ostrobothnia29 ^F^ | N1c | 14 | 14 | 16 | 24 | 11 | 14 | 14 |
| S Ostrobothnia01 ^F^ | N1c | 14 | 14 | 16 | 24 | 11 | 14 | 14 |
| S Ostrobothnia02 ^F^ | N1c | 14 | 14 | 16 | 24 | 11 | 14 | 14 |
| S Ostrobothnia03 ^F^ | N1c | 14 | 14 | 16 | 24 | 11 | 14 | 14 |
| SS Ostrobothnia01 ^F^ | N1c | 14 | 14 | 16 | 24 | 11 | 14 | 14 |
| SS Ostrobothnia02 ^F^ | N1c | 14 | 14 | 16 | 24 | 11 | 14 | 14 |
| Hame01 ^F^ | N1c | 14 | 14 | 16 | 24 | 11 | 14 | 14 |
| Hame02 ^F^ | N1c | 14 | 14 | 16 | 24 | 11 | 14 | 14 |
| Hame03 ^F^ | N1c | 14 | 14 | 16 | 24 | 11 | 14 | 14 |
| Hame04 ^F^ | N1c | 14 | 14 | 16 | 24 | 11 | 14 | 14 |
| Hame05 ^F^ | N1c | 14 | 14 | 16 | 24 | 11 | 14 | 14 |
| SW Finland01 ^F^ | N1c | 14 | 14 | 16 | 24 | 11 | 14 | 14 |
| SW Finland02 ^F^ | N1c | 14 | 14 | 16 | 24 | 11 | 14 | 14 |
| S Karelia12 ^F^ | N1c | 15 | 14 | 16 | 24 | 12 | 14 | 14 |
| S Karelia13 ^F^ | N1c | 14 | 14 | 16 | 25 | 12 | 14 | 15 |
| S Karelia14 ^F^ | N1c | 15 | 13 | 16 | 23 | 12 | 14 | 13 |
| S Karelia15 ^F^ | N1c | 14 | 14 | 16 | 24 | 12 | 14 | 14 |
| S Karelia16 ^F^ | N1c | 14 | 14 | 16 | 24 | 12 | 14 | 14 |
| S Karelia17 ^F^ | N1c | 14 | 14 | 16 | 24 | 12 | 14 | 14 |
| S Karelia18 ^F^ | N1c | 14 | 14 | 16 | 24 | 12 | 14 | 14 |
| S Karelia19 ^F^ | N1c | 14 | 14 | 16 | 24 | 12 | 14 | 14 |
| S Karelia20 ^F^ | N1c | 14 | 14 | 16 | 24 | 12 | 14 | 14 |
| S Karelia21 ^F^ | N1c | 14 | 14 | 16 | 24 | 12 | 14 | 14 |
| N Savo12 ^F^ | N1c | 14 | 14 | 16 | 24 | 12 | 14 | 14 |
| N Savo13 ^F^ | N1c | 14 | 14 | 16 | 24 | 12 | 14 | 14 |
| N Savo14 ^F^ | N1c | 14 | 14 | 16 | 24 | 12 | 14 | 14 |
| N Savo15 ^F^ | N1c | 14 | 14 | 16 | 24 | 12 | 14 | 14 |
| N Ostrobothnia30 ^F^ | N1c | 14 | 14 | 16 | 24 | 12 | 14 | 14 |
| S Ostrobothnia04 ^F^ | N1c | 14 | 14 | 16 | 24 | 12 | 14 | 14 |
| SS Ostrobothnia03 ^F^ | N1c | 14 | 14 | 16 | 24 | 12 | 14 | 14 |
| S Karelia22 ^F^ | N1c | 14 | 14 | 16 | 24 | 11 | 14 | 12 |
| S Karelia23 ^F^ | N1c | 14 | 15 | 16 | 25 | 12 | 14 | 14 |
| S Karelia24 ^F^ | N1c | 14 | 14 | 17 | 23 | 12 | 14 | 15 |
| S Karelia25 ^F^ | N1c | 14 | 14 | 16 | 24 | 11 | 15 | 14 |
| N Ostrobothnia31 ^F^ | N1c | 14 | 14 | 16 | 24 | 11 | 15 | 14 |
| Hame06 ^F^ | N1c | 14 | 14 | 16 | 24 | 11 | 15 | 14 |
| S Karelia26 ^F^ | N1c | 14 | 13 | 16 | 23 | 12 | 14 | 13 |
| S Karelia27 ^F^ | N1c | 14 | 14 | 16 | 24 | 12 | 14 | 14 |
| S Karelia28 ^F^ | N1c | 14 | 14 | 16 | 23 | 11 | 14 | 14 |
| N Karelia04 ^F^ | N1c | 14 | 14 | 16 | 23 | 11 | 14 | 14 |
| N Savo16 ^F^ | N1c | 14 | 14 | 16 | 23 | 11 | 14 | 14 |
| N Ostrobothnia32 ^F^ | N1c | 14 | 14 | 16 | 23 | 11 | 14 | 14 |
| N Ostrobothnia33 ^F^ | N1c | 14 | 14 | 16 | 23 | 11 | 14 | 14 |
| SS Ostrobothnia04 ^F^ | N1c | 14 | 14 | 16 | 23 | 11 | 14 | 14 |
| SS Ostrobothnia05 ^F^ | N1c | 14 | 14 | 16 | 23 | 11 | 14 | 14 |
| Hame07 ^F^ | N1c | 14 | 14 | 16 | 23 | 11 | 14 | 14 |
| Hame08 ^F^ | N1c | 14 | 14 | 16 | 23 | 11 | 14 | 14 |
| Hame09 ^F^ | N1c | 14 | 14 | 16 | 23 | 11 | 14 | 14 |
| SW Finland03 ^F^ | N1c | 14 | 14 | 16 | 23 | 11 | 14 | 14 |
| SW Finland04 ^F^ | N1c | 14 | 14 | 16 | 23 | 11 | 14 | 14 |
| S Karelia29 ^F^ | N1c | 15 | 14 | 15 | 24 | 10 | 14 | 14 |
| S Karelia30 ^F^ | N1c | 14 | 14 | 16 | 24 | 11 | 14 | 13 |
| S Karelia31 ^F^ | N1c | 14 | 14 | 17 | 24 | 11 | 14 | 13 |
| S Karelia32 ^F^ | N1c | 14 | 14 | 17 | 24 | 12 | 14 | 14 |
| S Karelia33 ^F^ | N1c | 14 | 13 | 18 | 24 | 12 | 14 | 14 |
| S Karelia34 ^F^ | N1c | 14 | 13 | 16 | 23 | 12 | 14 | 14 |
| N Karelia05 ^F^ | N1c | 15 | 14 | 16 | 24 | 11 | 14 | 14 |
| N Ostrobothnia34 ^F^ | N1c | 15 | 14 | 16 | 24 | 11 | 14 | 14 |
| S Ostrobothnia05 ^F^ | N1c | 15 | 14 | 16 | 24 | 11 | 14 | 14 |
| N Savo17 ^F^ | N1c | 14 | 13 | 16 | 24 | 11 | 14 | 15 |
| S Ostrobothnia06 ^F^ | N1c | 14 | 13 | 16 | 24 | 11 | 14 | 15 |
| N Karelia06 ^F^ | N1c | 14 | 14 | 16 | 24 | 10 | 14 | 14 |
| N Savo18 ^F^ | N1c | 14 | 14 | 16 | 24 | 10 | 14 | 14 |
| N Savo19 ^F^ | N1c | 14 | 14 | 16 | 24 | 10 | 14 | 14 |
| N Savo20 ^F^ | N1c | 14 | 14 | 16 | 24 | 10 | 14 | 14 |
| N Savo21 ^F^ | N1c | 14 | 14 | 16 | 24 | 10 | 14 | 14 |
| S Ostrobothnia07 ^F^ | N1c | 14 | 14 | 16 | 24 | 10 | 14 | 14 |
| S Ostrobothnia08 ^F^ | N1c | 14 | 14 | 16 | 24 | 10 | 14 | 14 |
| Satakunta01 ^F^ | N1c | 14 | 14 | 16 | 24 | 10 | 14 | 14 |
| Hame10 ^F^ | N1c | 14 | 14 | 16 | 24 | 10 | 14 | 14 |
| Hame11 ^F^ | N1c | 14 | 14 | 16 | 24 | 10 | 14 | 14 |
| SW Finland05 | N1c | 14 | 14 | 16 | 24 | 10 | 14 | 14 |
| S Ostrobothnia09 ^F^ | N1c | 14 | 13 | 17 | 24 | 10 | 14 | 14 |
| S Ostrobothnia10 ^F^ | N1c | 14 | 12 | 16 | 24 | 11 | 14 | 14 |
| N Ostrobothnia35 ^F^ | N1c | 14 | 14 | 16 | 24 | 11 | 14 | 14 |
| N Ostrobothnia36 ^F^ | N1c | 14 | 14 | 16 | 24 | 11 | 14 | 14 |
| S Ostrobothnia11 ^F^ | N1c | 14 | 14 | 16 | 24 | 11 | 14 | 14 |
| S Ostrobothnia12 ^F^ | N1c | 14 | 14 | 16 | 24 | 11 | 14 | 14 |
| S Ostrobothnia13 ^F^ | N1c | 14 | 14 | 16 | 24 | 12 | 14 | 14 |
| SW Finland06 ^F^ | N1c | 14 | 14 | 16 | 24 | 12 | 14 | 14 |
| N Savo22 ^F^ | N1c | 14 | 14 | 17 | 23 | 11 | 14 | 14 |
| S Ostrobothnia14 ^F^ | N1c | 14 | 14 | 17 | 23 | 11 | 14 | 14 |
| N Karelia07 ^F^ | N1c | 14 | 13 | 16 | 23 | 11 | 14 | 14 |
| N Karelia08 ^F^ | N1c | 14 | 13 | 16 | 23 | 11 | 14 | 14 |
| N Ostrobothnia37 ^F^ | N1c | 14 | 13 | 16 | 23 | 11 | 14 | 14 |
| N Ostrobothnia38 ^F^ | N1c | 14 | 13 | 16 | 23 | 11 | 14 | 14 |
| N Ostrobothnia39 ^F^ | N1c | 14 | 13 | 16 | 23 | 11 | 14 | 14 |
| S Ostrobothnia15 ^F^ | N1c | 14 | 13 | 16 | 23 | 11 | 14 | 14 |
| SS Ostrobothnia06 ^F^ | N1c | 14 | 13 | 16 | 23 | 11 | 14 | 14 |
| Hame12 ^F^ | N1c | 14 | 13 | 16 | 23 | 11 | 14 | 14 |
| Hame13 ^F^ | N1c | 14 | 13 | 16 | 23 | 11 | 14 | 14 |
| SW Finland07 ^F^ | N1c | 14 | 13 | 16 | 23 | 11 | 14 | 14 |
| SW Finland08 ^F^ | N1c | 14 | 13 | 16 | 23 | 11 | 14 | 14 |
| SW Finland09 ^F^ | N1c | 14 | 13 | 16 | 23 | 11 | 14 | 14 |
| N Karelia09 ^F^ | N1c | 14 | 13 | 16 | 24 | 11 | 14 | 14 |
| N Karelia10 ^F^ | N1c | 14 | 13 | 16 | 24 | 11 | 14 | 14 |
| N Karelia11 ^F^ | N1c | 14 | 13 | 16 | 24 | 11 | 14 | 14 |
| N Savo23 ^F^ | N1c | 14 | 13 | 16 | 24 | 11 | 14 | 14 |
| N Savo24 ^F^ | N1c | 14 | 13 | 16 | 24 | 11 | 14 | 14 |
| Hame14 ^F^ | N1c | 14 | 13 | 16 | 24 | 11 | 14 | 14 |
| Hame15 ^F^ | N1c | 14 | 13 | 16 | 24 | 11 | 14 | 14 |
| Hame16 ^F^ | N1c | 16 | 11 | 16 | 23 | 11 | 14 | 14 |
| Hame17 ^F^ | N1c | 14 | 13 | 16 | 25 | 11 | 14 | 14 |
| Hame18 ^F^ | N1c | 15 | 14 | 16 | 23 | 11 | 14 | 13 |
| N Ostrobothnia40 ^F^ | N1c | 14 | 13 | 16 | 23 | 11 | 14 | 13 |
| Satakunta02 ^F^ | N1c | 14 | 13 | 16 | 23 | 11 | 14 | 13 |
| Satakunta03 ^F^ | N1c | 14 | 13 | 16 | 23 | 11 | 14 | 13 |
| Hame19 ^F^ | N1c | 14 | 13 | 16 | 23 | 11 | 14 | 13 |
| N Savo25 ^F^ | N1c | 14 | 14 | 17 | 24 | 11 | 14 | 14 |
| N Savo26 ^F^ | N1c | 14 | 14 | 17 | 24 | 11 | 14 | 14 |
| N Ostrobothnia41 ^F^ | N1c | 14 | 14 | 17 | 24 | 11 | 14 | 14 |
| N Ostrobothnia42 ^F^ | N1c | 14 | 14 | 17 | 24 | 11 | 14 | 14 |
| N Ostrobothnia43 | N1c | 14 | 14 | 17 | 24 | 11 | 14 | 14 |
| Hame20 ^F^ | N1c | 14 | 14 | 17 | 24 | 11 | 14 | 14 |
| Hame21 ^F^ | N1c | 15 | 14 | 16 | 24 | 11 | 14 | 14 |
| Hame22 ^F^ | N1c | 14 | 14 | 17 | 24 | 11 | 14 | 14 |
| SS Ostrobothnia07 ^F^ | N1c | 14 | 13 | 16 | 23 | 10 | 14 | 14 |
| SS Ostrobothnia08 ^F^ | N1c | 14 | 13 | 16 | 23 | 10 | 14 | 14 |
| Satakunta04 ^F^ | N1c | 14 | 13 | 16 | 23 | 10 | 14 | 14 |
| Satakunta05 ^F^ | N1c | 14 | 13 | 16 | 23 | 10 | 14 | 14 |
| Satakunta06 ^F^ | N1c | 14 | 13 | 16 | 23 | 10 | 14 | 14 |
| Hame23 ^F^ | N1c | 14 | 13 | 16 | 23 | 10 | 14 | 14 |
| SW Finland10 ^F^ | N1c | 14 | 13 | 16 | 23 | 10 | 14 | 14 |
| SW Finland11 ^F^ | N1c | 14 | 13 | 16 | 23 | 10 | 14 | 14 |
| SW Finland12 ^F^ | N1c | 14 | 13 | 16 | 23 | 10 | 14 | 14 |
| SW Finland13 ^F^ | N1c | 14 | 13 | 16 | 23 | 10 | 14 | 14 |
| Hame24 ^F^ | N1c | 14 | 13 | 16 | 23 | 11 | 14 | 14 |
| Hame25 ^F^ | N1c | 15 | 13 | 16 | 22 | 11 | 14 | 14 |
| Hame26 ^F^ | N1c | 14 | 13 | 17 | 23 | 11 | 14 | 14 |
| N Ostrobothnia44 ^F^ | N1c | 14 | 15 | 16 | 24 | 11 | 14 | 14 |
| Hame27 ^F^ | N1c | 14 | 15 | 16 | 24 | 11 | 14 | 14 |
| N Karelia12 ^F^ | N1c | 15 | 14 | 16 | 24 | 11 | 14 | 15 |
| N Karelia13 ^F^ | N1c | 14 | 13 | 16 | 25 | 11 | 14 | 14 |
| SS Ostrobothnia09 ^F^ | N1c | 14 | 13 | 16 | 25 | 11 | 14 | 14 |
| N Karelia14 ^F^ | N1c | 14 | 14 | 16 | 24 | 10 | 14 | 15 |
| N Karelia15 ^F^ | N1c | 14 | 13 | 17 | 24 | 11 | 14 | 13 |
| N Savo27 ^F^ | N1c | 14 | 14 | 16 | 24 | 11 | 14 | 15 |
| N Ostrobothnia45 ^F^ | N1c | 14 | 14 | 16 | 24 | 11 | 14 | 15 |
| N Ostrobothnia46 ^F^ | N1c | 14 | 14 | 16 | 24 | 11 | 14 | 15 |
| N Savo28 ^F^ | N1c | 14 | 14 | 16 | 24 | 11 | 14 | 13 |
| N Ostrobothnia47 ^F^ | N1c | 14 | 14 | 16 | 24 | 11 | 14 | 13 |
| N Ostrobothnia48 ^F^ | N1c | 14 | 14 | 16 | 24 | 11 | 14 | 13 |
| N Ostrobothnia49 ^F^ | N1c | 14 | 15 | 16 | 24 | 11 | 14 | 13 |
| N Ostrobothnia50 ^F^ | N1c | 14 | 14 | 16 | 24 | 12 | 14 | 14 |
| N Ostrobothnia51 ^F^ | N1c | 14 | 13 | 16 | 23 | 10 | 14 | 14 |
| N Savo29 ^F^ | N1c | 14 | 14 | 16 | 24 | 11 | 14 | 14 |
| N Savo30 ^F^ | N1c | 14 | 14 | 16 | 24 | 11 | 14 | 14 |
| N Savo31 ^F^ | N1c | 14 | 14 | 16 | 24 | 11 | 14 | 14 |
| N Savo32 ^F^ | N1c | 14 | 14 | 16 | 24 | 11 | 14 | 14 |
| N Savo33 ^F^ | N1c | 14 | 14 | 16 | 24 | 11 | 14 | 14 |
| N Savo34 ^F^ | N1c | 14 | 14 | 16 | 24 | 11 | 14 | 14 |
| N Savo35 ^F^ | N1c | 14 | 14 | 16 | 24 | 11 | 14 | 14 |
| N Savo36 ^F^ | N1c | 14 | 14 | 16 | 24 | 11 | 14 | 14 |
| N Savo37 ^F^ | N1c | 14 | 14 | 16 | 24 | 11 | 14 | 14 |
| N Savo38 ^F^ | N1c | 14 | 14 | 16 | 24 | 11 | 14 | 14 |
| N Savo39 ^F^ | N1c | 14 | 14 | 16 | 24 | 11 | 14 | 14 |
| N Ostrobothnia52 ^F^ | N1c | 14 | 14 | 16 | 24 | 11 | 14 | 14 |
| N Ostrobothnia53 ^F^ | N1c | 14 | 14 | 16 | 24 | 11 | 14 | 14 |
| N Ostrobothnia54 ^F^ | N1c | 14 | 14 | 16 | 24 | 11 | 14 | 14 |
| N Ostrobothnia55 ^F^ | N1c | 13 | 14 | 16 | 24 | 11 | 14 | 14 |
| N Ostrobothnia56 ^F^ | N1c | 14 | 14 | 16 | 23 | 11 | 14 | 15 |
| N Ostrobothnia57 ^F^ | N1c | 14 | 13 | 16 | 24 | 11 | 14 | 15 |
| N Savo40 ^F^ | N1c | 14 | 14 | 16 | 24 | 11 | 14 | 14 |
| N Ostrobothnia58 ^F^ | N1c | 14 | 14 | 16 | 24 | 11 | 14 | 14 |
| N Ostrobothnia59 ^F^ | N1c | 14 | 13 | 17 | 24 | 11 | 14 | 14 |
| N Ostrobothnia60 ^F^ | N1c | 14 | 13 | 16 | 23 | 10 | 14 | 15 |
| N Savo41 ^F^ | N1c | 14 | 14 | 16 | 23 | 10 | 14 | 14 |
| N Ostrobothnia61 ^F^ | N1c | 14 | 14 | 16 | 23 | 10 | 14 | 14 |
| N Ostrobothnia62 ^F^ | N1c | 14 | 13 | 16 | 23 | 10 | 14 | 14 |
| N Savo42 ^F^ | N1c | 14 | 13 | 16 | 24 | 11 | 14 | 15 |
| N Ostrobothnia63 ^F^ | N1c | 14 | 13 | 16 | 24 | 11 | 14 | 15 |
| N Ostrobothnia64 ^F^ | N1c | 14 | 13 | 16 | 24 | 11 | 14 | 15 |
| N Ostrobothnia65 ^F^ | N1c | 14 | 15 | 16 | 24 | 10 | 14 | 14 |
| N Ostrobothnia66 ^F^ | N1c | 14 | 14 | 16 | 25 | 11 | 14 | 14 |
| N Ostrobothnia67 ^F^ | N1c | 14 | 13 | 16 | 24 | 11 | 14 | 13 |
| N Ostrobothnia68 ^F^ | N1c | 13 | 14 | 16 | 23 | 11 | 14 | 14 |
| N Ostrobothnia69 ^F^ | N1c | 14 | 14 | 16 | 25 | 11 | 14 | 13 |
| N Ostrobothnia70 ^F^ | N1c | 14 | 14 | 16 | 25 | 11 | 15 | 14 |
| N Ostrobothnia71 ^F^ | N1c | 15 | 15 | 16 | 24 | 11 | 15 | 13 |
| N Ostrobothnia72 ^F^ | N1c | 14 | 13 | 16 | 23 | 10 | 14 | 14 |
| N Ostrobothnia73 ^F^ | N1c | 14 | 14 | 16 | 24 | 11 | 11 | 14 |
| N Ostrobothnia74 ^F^ | N1c | 14 | 14 | 16 | 24 | 11 | 11 | 14 |
| N Ostrobothnia75 ^F^ | N1c | 14 | 14 | 16 | 24 | 11 | 11 | 14 |
| N Ostrobothnia76 ^F^ | N1c | 14 | 15 | 16 | 24 | 11 | 14 | 14 |
| N Ostrobothnia77 ^F^ | N1c | 14 | 14 | 15 | 24 | 11 | 13 | 14 |
| N Ostrobothnia78 ^F^ | N1c | 14 | 13 | 16 | 24 | 11 | 13 | 15 |
| N Ostrobothnia79 ^F^ | N1c | 15 | 14 | 16 | 24 | 11 | 13 | 14 |
| N Ostrobothnia80 ^F^ | N1c | 14 | 14 | 17 | 24 | 11 | 13 | 14 |
| N Ostrobothnia81 ^F^ | N1c | 14 | 14 | 16 | 24 | 11 | 11 | 14 |
| N Ostrobothnia82 ^F^ | N1c | 14 | 14 | 16 | 23 | 10 | 11 | 14 |
| N Ostrobothnia83 ^F^ | N1c | 14 | 14 | 16 | 24 | 11 | 13 | 14 |
| N Ostrobothnia84 ^F^ | N1c | 15 | 13 | 16 | 24 | 11 | 14 | 15 |
| N Savo43 ^F^ | N1c | 14 | 14 | 16 | 22 | 11 | 14 | 14 |
| N Savo44 ^F^ | N1c | 15 | 14 | 15 | 25 | 12 | 14 | 14 |
| N Savo45 ^F^ | N1c | 14 | 14 | 16 | 24 | 12 | 14 | 14 |
| N Savo46 ^F^ | N1c | 14 | 14 | 16 | 24 | 11 | 14 | 14 |
| N Savo47 ^F^ | N1c | 14 | 14 | 16 | 24 | 11 | 14 | 14 |
| N Savo48 ^F^ | N1c | 14 | 14 | 16 | 24 | 11 | 14 | 14 |
| N Savo49 ^F^ | N1c | 14 | 14 | 16 | 24 | 12 | 14 | 14 |
| N Savo50 ^F^ | N1c | 14 | 14 | 16 | 24 | 12 | 14 | 14 |
| N Savo51 ^F^ | N1c | 14 | 13 | 16 | 24 | 11 | 14 | 13 |
| N Savo52 ^F^ | N1c | 14 | 13 | 16 | 23 | 12 | 14 | 14 |
| N Savo53 ^F^ | N1c | 14 | 14 | 16 | 22 | 12 | 14 | 14 |
| N Savo54 ^F^ | N1c | 14 | 14 | 16 | 25 | 11 | 14 | 14 |
| N Savo55 ^F^ | N1c | 14 | 14 | 16 | 25 | 11 | 14 | 14 |
| N Savo56 ^F^ | N1c | 14 | 13 | 16 | 24 | 12 | 14 | 15 |
| N Savo57 ^F^ | N1c | 14 | 14 | 16 | 24 | 12 | 14 | 14 |
| N Savo58 ^F^ | N1c | 14 | 14 | 16 | 24 | 12 | 14 | 14 |
| N Savo59 ^F^ | N1c | 15 | 14 | 16 | 24 | 10 | 14 | 14 |
| N Savo60 ^F^ | N1c | 15 | 13 | 16 | 23 | 11 | 14 | 14 |
| N Savo61 ^F^ | N1c | 14 | 15 | 16 | 24 | 11 | 14 | 14 |
| N Savo62 ^F^ | N1c | 14 | 14 | 16 | 24 | 11 | 15 | 14 |
| N Savo63 ^F^ | N1c | 14 | 14 | 16 | 24 | 10 | 14 | 14 |
| N Savo64 ^F^ | N1c | 14 | 14 | 16 | 24 | 10 | 14 | 14 |
| N Savo65 ^F^ | N1c | 15 | 14 | 17 | 25 | 11 | 14 | 14 |
| N Savo66 ^F^ | N1c | 14 | 14 | 16 | 23 | 10 | 14 | 14 |
| N Savo67 ^F^ | N1c | 15 | 14 | 17 | 24 | 10 | 14 | 14 |
| N Savo68 ^F^ | N1c | 16 | 14 | 17 | 24 | 10 | 14 | 14 |
| N Savo69 ^F^ | N1c | 13 | 14 | 17 | 23 | 11 | 14 | 14 |
| N Savo70 ^F^ | N1c | 14 | 14 | 16 | 24 | 11 | 14 | 14 |
| N Savo71 ^F^ | N1c | 14 | 14 | 15 | 24 | 11 | 14 | 14 |
| N Savo72 ^F^ | N1c | 14 | 14 | 16 | 25 | 11 | 14 | 14 |
| N Savo73 ^F^ | N1c | 15 | 15 | 16 | 24 | 11 | 14 | 14 |
| N Savo74 ^F^ | N1c | 15 | 13 | 16 | 23 | 11 | 14 | 13 |
| N Savo75 ^F^ | N1c | 13 | 14 | 16 | 24 | 10 | 14 | 14 |
| N Savo76 ^F^ | N1c | 14 | 14 | 16 | 24 | 11 | 14 | 14 |
| N Savo77 ^F^ | N1c | 14 | 14 | 16 | 24 | 10 | 14 | 14 |
| N Savo78 ^F^ | N1c | 14 | 14 | 16 | 24 | 10 | 14 | 14 |
| Satakunta07 ^F^ | N1c | 14 | 14 | 16 | 24 | 10 | 14 | 14 |
| N Savo79 ^F^ | N1c | 14 | 14 | 15 | 24 | 11 | 14 | 14 |
| N Savo80 ^F^ | N1c | 14 | 14 | 16 | 23 | 10 | 14 | 14 |
| SW Finland14 ^F^ | N1c | 14 | 14 | 16 | 23 | 10 | 14 | 14 |
| N Savo81 ^F^ | N1c | 14 | 13 | 17 | 23 | 11 | 14 | 13 |
| N Savo82 ^F^ | N1c | 13 | 14 | 16 | 24 | 11 | 14 | 14 |
| N Savo83 ^F^ | N1c | 14 | 14 | 16 | 23 | 11 | 14 | 14 |
| N Savo84 ^F^ | N1c | 14 | 14 | 17 | 24 | 11 | 14 | 14 |
| SS Ostrobothnia10 ^F^ | N1c | 13 | 13 | 16 | 23 | 10 | 14 | 14 |
| Satakunta08 ^F^ | N1c | 15 | 13 | 16 | 24 | 10 | 14 | 14 |
| Satakunta09 ^F^ | N1c | 15 | 14 | 16 | 24 | 10 | 14 | 14 |
| Satakunta10 ^F^ | N1c | 15 | 13 | 16 | 23 | 10 | 14 | 14 |
| Satakunta11 ^F^ | N1c | 15 | 13 | 16 | 23 | 10 | 14 | 14 |
| SW Finland15 ^F^ | N1c | 15 | 13 | 16 | 23 | 10 | 14 | 14 |
| Satakunta12 ^F^ | N1c | 15 | 13 | 16 | 24 | 10 | 14 | 14 |
| Satakunta13 ^F^ | N1c | 14 | 13 | 15 | 23 | 11 | 14 | 14 |
| SW Finland16 ^F^ | N1c | 14 | 13 | 16 | 23 | 11 | 15 | 14 |
| SW Finland17 ^F^ | N1c | 14 | 13 | 16 | 23 | 11 | 14 | 14 |
| SW Finland18 ^F^ | N1c | 14 | 13 | 16 | 24 | 10 | 14 | 14 |
| SW Finland19 ^F^ | N1c | 13 | 14 | 16 | 24 | 9 | 11 | 13 |
| SW Finland20 ^F^ | N1c | 15 | 14 | 16 | 23 | 10 | 14 | 14 |
| SW Finland21 ^F^ | N1c | 14 | 14 | 16 | 23 | 11 | 14 | 14 |
| SW Finland22 ^F^ | N1c | 14 | 14 | 16 | 23 | 11 | 14 | 14 |
| SW Finland23 ^F^ | N1c | 14 | 13 | 16 | 23 | 10 | 14 | 13 |
| SW Finland24 ^F^ | N1c | 15 | 13 | 16 | 24 | 10 | 14 | 13 |
| SW Finland25 ^F^ | N1c | 14 | 13 | 16 | 23 | 11 | 14 | 14 |
| SW Finland26 ^F^ | N1c | 14 | 13 | 16 | 23 | 11 | 14 | 14 |
| SW Finland27 ^F^ | N1c | 14 | 13 | 17 | 23 | 10 | 14 | 14 |
| SW Finland28 ^F^ | N1c | 15 | 13 | 16 | 23 | 10 | 14 | 15 |
| SW Finland29 ^F^ | N1c | 14 | 14 | 16 | 23 | 10 | 14 | 14 |
| Karelian01^G^ | N1b | 13 | 13 | 18 | 23 | 10 | 12 | 12 |
| W Finnish01 ^G^ | N1b | 13 | 13 | 18 | 23 | 10 | 12 | 12 |
| W Finnish02 ^G^ | N1b | 15 | 13 | 18 | 23 | 10 | 12 | 12 |
| E Finnish01 ^G^ | N1c | 13 | 14 | 16 | 24 | 11 | 14 | 14 |
| W Finnish03 ^G^ | N1c | 13 | 13 | 16 | 23 | 10 | 14 | 14 |
| Karelian02 ^G^ | N1c | 13 | 14 | 16 | 23 | 10 | 11 | 14 |
| E Finnish02 ^G^ | N1c | 13 | 14 | 16 | 24 | 10 | 14 | 14 |
| E Finnish03 ^G^ | N1c | 13 | 14 | 16 | 24 | 11 | 14 | 14 |
| E Finnish04 ^G^ | N1c | 13 | 14 | 17 | 23 | 11 | 14 | 14 |
| Lithuanian01 ^G^ | N1c | 13 | 13 | 17 | 23 | 10 | 14 | 14 |
| W Finnish04 ^G^ | N1c | 13 | 14 | 16 | 23 | 11 | 14 | 14 |
| W Finnish05 ^G^ | N1c | 13 | 14 | 16 | 24 | 9 | 11 | 13 |
| E Finnish05 ^G^ | N1c | 14 | 14 | 16 | 24 | 11 | 14 | 14 |
| E Finnish06 ^G^ | N1c | 14 | 14 | 16 | 24 | 11 | 14 | 14 |
| W Finnish06 ^G^ | N1c | 14 | 13 | 16 | 23 | 11 | 14 | 14 |
| Swedish01 ^G^ | N1c | 14 | 13 | 16 | 23 | 11 | 14 | 14 |
| W Finnish07 ^G^ | N1c | 14 | 13 | 16 | 25 | 11 | 14 | 14 |
| Estonian01 ^G^ | N1c | 14 | 14 | 16 | 23 | 11 | 12 | 14 |
| E Finnish07 ^G^ | N1c | 14 | 14 | 16 | 24 | 12 | 14 | 14 |
| E Finnish08 ^G^ | N1c | 14 | 14 | 16 | 24 | 11 | 14 | 14 |
| W Finnish08 ^G^ | N1c | 14 | 13 | 16 | 23 | 11 | 14 | 14 |
| W Finnish09 ^G^ | N1c | 14 | 14 | 16 | 23 | 11 | 14 | 14 |
| E Finnish09 ^G^ | N1c | 14 | 13 | 16 | 24 | 11 | 14 | 15 |
| E Finnish10 ^G^ | N1c | 14 | 13 | 16 | 23 | 10 | 14 | 14 |
| Estonian02 ^G^ | N1c | 14 | 13 | 16 | 23 | 11 | 14 | 14 |
| Karelian03 ^G^ | N1c | 14 | 13 | 16 | 23 | 11 | 14 | 14 |
| Karelian04 ^G^ | N1c | 14 | 13 | 16 | 23 | 11 | 14 | 14 |
| W Finnish10 ^G^ | N1c | 14 | 13 | 16 | 23 | 11 | 14 | 14 |
| E Finnish11 ^G^ | N1c | 14 | 13 | 16 | 23 | 10 | 14 | 14 |
| Estonian03 ^G^ | N1c | 14 | 11 | 16 | 23 | 11 | 14 | 13 |
| Lithuanian02 ^G^ | N1c | 14 | 11 | 16 | 23 | 11 | 14 | 13 |
| W Finnish11 ^G^ | N1c | 14 | 12 | 16 | 24 | 11 | 14 | 14 |
| W Finnish12 ^G^ | N1c | 14 | 13 | 15 | 23 | 11 | 14 | 14 |
| Swedish02 ^G^ | N1c | 14 | 13 | 15 | 24 | 11 | 14 | 13 |
| Estonian04 ^G^ | N1c | 14 | 13 | 16 | 23 | 10 | 14 | 14 |
| Estonian05 ^G^ | N1c | 14 | 13 | 16 | 23 | 10 | 14 | 14 |
| Estonian06 ^G^ | N1c | 14 | 13 | 16 | 23 | 10 | 14 | 14 |
| Karelian05 ^G^ | N1c | 14 | 13 | 16 | 23 | 10 | 14 | 14 |
| W Finnish13 ^G^ | N1c | 14 | 13 | 16 | 23 | 10 | 14 | 14 |
| W Finnish14 ^G^ | N1c | 14 | 13 | 16 | 23 | 10 | 14 | 14 |
| W Finnish15 ^G^ | N1c | 14 | 13 | 16 | 23 | 10 | 14 | 14 |
| W Finnish16 ^G^ | N1c | 14 | 13 | 16 | 23 | 10 | 14 | 14 |
| W Finnish17 ^G^ | N1c | 14 | 13 | 16 | 23 | 10 | 14 | 14 |
| W Finnish18 ^G^ | N1c | 14 | 13 | 16 | 23 | 10 | 14 | 14 |
| W Finnish19 ^G^ | N1c | 14 | 13 | 16 | 23 | 10 | 14 | 14 |
| W Finnish20 ^G^ | N1c | 14 | 13 | 16 | 23 | 10 | 14 | 14 |
| W Finnish21 ^G^ | N1c | 14 | 13 | 16 | 23 | 10 | 14 | 14 |
| W Finnish22 ^G^ | N1c | 14 | 13 | 16 | 23 | 10 | 14 | 14 |
| Swedish03 ^G^ | N1c | 14 | 13 | 16 | 23 | 10 | 14 | 14 |
| Swedish04 ^G^ | N1c | 14 | 13 | 16 | 23 | 10 | 14 | 14 |
| Swedish05 ^G^ | N1c | 14 | 13 | 16 | 23 | 10 | 14 | 14 |
| Swedish06 ^G^ | N1c | 14 | 13 | 16 | 23 | 10 | 14 | 14 |
| Swedish07 ^G^ | N1c | 14 | 13 | 16 | 23 | 10 | 14 | 14 |
| E Finnish12 ^G^ | N1c | 14 | 13 | 16 | 23 | 10 | 14 | 15 |
| Estonian07 ^G^ | N1c | 14 | 13 | 16 | 23 | 11 | 14 | 13 |
| E Finnish13 ^G^ | N1c | 14 | 13 | 16 | 23 | 11 | 14 | 13 |
| W Finnish23 ^G^ | N1c | 14 | 13 | 16 | 23 | 11 | 14 | 13 |
| W Finnish24 ^G^ | N1c | 14 | 13 | 16 | 23 | 11 | 14 | 13 |
| W Finnish25 ^G^ | N1c | 14 | 13 | 16 | 23 | 11 | 14 | 13 |
| Estonian08 ^G^ | N1c | 14 | 13 | 16 | 23 | 11 | 14 | 14 |
| Karelian06 ^G^ | N1c | 14 | 13 | 16 | 23 | 11 | 14 | 14 |
| Karelian07 ^G^ | N1c | 14 | 13 | 16 | 23 | 11 | 14 | 14 |
| Karelian08 ^G^ | N1c | 14 | 13 | 16 | 23 | 11 | 14 | 14 |
| E Finnish14 | N1c | 14 | 13 | 16 | 23 | 11 | 14 | 14 |
| E Finnish15 ^G^ | N1c | 14 | 13 | 16 | 23 | 11 | 14 | 14 |
| E Finnish16 ^G^ | N1c | 14 | 13 | 16 | 23 | 11 | 14 | 14 |
| E Finnish17 ^G^ | N1c | 14 | 13 | 16 | 23 | 11 | 14 | 14 |
| E Finnish18 ^G^ | N1c | 14 | 13 | 16 | 23 | 11 | 14 | 14 |
| W Finnish26 ^G^ | N1c | 14 | 13 | 16 | 23 | 11 | 14 | 14 |
| W Finnish27 ^G^ | N1c | 14 | 13 | 16 | 23 | 11 | 14 | 14 |
| W Finnish28 ^G^ | N1c | 14 | 13 | 16 | 23 | 11 | 14 | 14 |
| W Finnish29 ^G^ | N1c | 14 | 13 | 16 | 23 | 11 | 14 | 14 |
| W Finnish30 ^G^ | N1c | 14 | 13 | 16 | 23 | 11 | 14 | 14 |
| W Finnish31 ^G^ | N1c | 14 | 13 | 16 | 23 | 11 | 14 | 14 |
| W Finnish32 ^G^ | N1c | 14 | 13 | 16 | 23 | 11 | 14 | 14 |
| Estonian09 ^G^ | N1c | 14 | 13 | 16 | 23 | 11 | 15 | 14 |
| W Finnish33 ^G^ | N1c | 14 | 13 | 16 | 23 | 11 | 15 | 14 |
| E Finnish19 ^G^ | N1c | 14 | 13 | 16 | 23 | 12 | 14 | 13 |
| E Finnish20 ^G^ | N1c | 14 | 13 | 16 | 23 | 12 | 14 | 14 |
| W Finnish34 ^G^ | N1c | 14 | 13 | 16 | 24 | 10 | 14 | 14 |
| E Finnish21 ^G^ | N1c | 14 | 13 | 16 | 24 | 11 | 13 | 15 |
| E Finnish22 ^G^ | N1c | 14 | 13 | 16 | 24 | 11 | 14 | 13 |
| Karelian09 ^G^ | N1c | 14 | 13 | 16 | 24 | 11 | 14 | 14 |
| Karelian10 ^G^ | N1c | 14 | 13 | 16 | 24 | 11 | 14 | 14 |
| E Finnish23 ^G^ | N1c | 14 | 13 | 16 | 24 | 11 | 14 | 14 |
| E Finnish24 ^G^ | N1c | 14 | 13 | 16 | 24 | 11 | 14 | 14 |
| E Finnish25 ^G^ | N1c | 14 | 13 | 16 | 24 | 11 | 14 | 14 |
| E Finnish26 ^G^ | N1c | 14 | 13 | 16 | 24 | 11 | 14 | 14 |
| E Finnish27 ^G^ | N1c | 14 | 13 | 16 | 24 | 11 | 14 | 14 |
| W Finnish35 ^G^ | N1c | 14 | 13 | 16 | 24 | 11 | 14 | 14 |
| W Finnish36 ^G^ | N1c | 14 | 13 | 16 | 24 | 11 | 14 | 14 |
| Swedish08 ^G^ | N1c | 14 | 13 | 16 | 24 | 11 | 14 | 14 |
| Estonian10 ^G^ | N1c | 14 | 13 | 16 | 24 | 11 | 14 | 15 |
| E Finnish28 ^G^ | N1c | 14 | 13 | 16 | 24 | 11 | 14 | 15 |
| W Finnish37 ^G^ | N1c | 14 | 13 | 16 | 24 | 11 | 14 | 15 |
| Swedish09 ^G^ | N1c | 14 | 13 | 16 | 24 | 11 | 14 | 15 |
| Estonian11 ^G^ | N1c | 14 | 13 | 16 | 24 | 13 | 14 | 14 |
| E Finnish29 ^G^ | N1c | 14 | 13 | 16 | 25 | 11 | 14 | 14 |
| W Finnish38 ^G^ | N1c | 14 | 13 | 16 | 25 | 11 | 14 | 14 |
| W Finnish39 ^G^ | N1c | 14 | 13 | 17 | 23 | 10 | 14 | 14 |
| E Finnish30 ^G^ | N1c | 14 | 13 | 17 | 23 | 11 | 14 | 13 |
| Karelian11 ^G^ | N1c | 14 | 13 | 17 | 23 | 11 | 14 | 14 |
| W Finnish40 ^G^ | N1c | 14 | 13 | 17 | 23 | 11 | 14 | 14 |
| W Finnish41 ^G^ | N1c | 14 | 13 | 17 | 24 | 10 | 14 | 14 |
| Karelian12 ^G^ | N1c | 14 | 13 | 17 | 24 | 11 | 14 | 14 |
| E Finnish31 ^G^ | N1c | 14 | 13 | 17 | 24 | 11 | 14 | 14 |
| E Finnish32 ^G^ | N1c | 14 | 13 | 18 | 24 | 12 | 14 | 14 |
| Karelian13 ^G^ | N1c | 14 | 14 | 15 | 23 | 10 | 14 | 14 |
| Karelian14 ^G^ | N1c | 14 | 14 | 15 | 23 | 11 | 14 | 14 |
| Karelian15 ^G^ | N1c | 14 | 14 | 15 | 23 | 11 | 14 | 14 |
| Karelian16 ^G^ | N1c | 14 | 14 | 15 | 23 | 12 | 14 | 14 |
| E Finnish33 ^G^ | N1c | 14 | 14 | 15 | 24 | 11 | 13 | 14 |
| E Finnish34 ^G^ | N1c | 14 | 14 | 15 | 24 | 11 | 14 | 14 |
| E Finnish35 ^G^ | N1c | 14 | 14 | 16 | 23 | 10 | 11 | 14 |
| Karelian17 ^G^ | N1c | 14 | 14 | 16 | 23 | 10 | 14 | 14 |
| Karelian18 ^G^ | N1c | 14 | 14 | 16 | 23 | 10 | 14 | 14 |
| Karelian19 ^G^ | N1c | 14 | 14 | 16 | 23 | 10 | 14 | 14 |
| E Finnish36 ^G^ | N1c | 14 | 14 | 16 | 23 | 10 | 14 | 14 |
| Estonian12 ^G^ | N1c | 14 | 14 | 16 | 23 | 11 | 14 | 13 |
| Estonian13 ^G^ | N1c | 14 | 14 | 16 | 23 | 11 | 14 | 14 |
| Estonian14 ^G^ | N1c | 14 | 14 | 16 | 23 | 11 | 14 | 14 |
| Lithuanian03 ^G^ | N1c | 14 | 14 | 16 | 23 | 11 | 14 | 14 |
| Karelian20 ^G^ | N1c | 14 | 14 | 16 | 23 | 11 | 14 | 14 |
| Karelian21 ^G^ | N1c | 14 | 14 | 16 | 23 | 11 | 14 | 14 |
| Karelian22 ^G^ | N1c | 14 | 14 | 16 | 23 | 11 | 14 | 14 |
| Karelian23 ^G^ | N1c | 14 | 14 | 16 | 23 | 11 | 14 | 14 |
| Karelian24 ^G^ | N1c | 14 | 14 | 16 | 23 | 11 | 14 | 14 |
| Karelian25 ^G^ | N1c | 14 | 14 | 16 | 23 | 11 | 14 | 14 |
| Karelian26 ^G^ | N1c | 14 | 14 | 16 | 23 | 11 | 14 | 14 |
| E Finnish37 ^G^ | N1c | 14 | 14 | 16 | 23 | 11 | 14 | 14 |
| E Finnish38 ^G^ | N1c | 14 | 14 | 16 | 23 | 11 | 14 | 14 |
| E Finnish39 ^G^ | N1c | 14 | 14 | 16 | 23 | 11 | 14 | 14 |
| E Finnish40 ^G^ | N1c | 14 | 14 | 16 | 23 | 11 | 14 | 14 |
| E Finnish41 ^G^ | N1c | 14 | 14 | 16 | 23 | 11 | 14 | 14 |
| W Finnish42 ^G^ | N1c | 14 | 14 | 16 | 23 | 11 | 14 | 14 |
| W Finnish43 ^G^ | N1c | 14 | 14 | 16 | 23 | 11 | 14 | 14 |
| W Finnish44 ^G^ | N1c | 14 | 14 | 16 | 23 | 11 | 14 | 14 |
| W Finnish45 ^G^ | N1c | 14 | 14 | 16 | 23 | 11 | 14 | 14 |
| W Finnish46 ^G^ | N1c | 14 | 14 | 16 | 23 | 11 | 14 | 14 |
| W Finnish47 ^G^ | N1c | 14 | 14 | 16 | 23 | 11 | 14 | 14 |
| W Finnish48 ^G^ | N1c | 14 | 14 | 16 | 23 | 11 | 14 | 14 |
| Karelian27 ^G^ | N1c | 14 | 14 | 16 | 23 | 11 | 15 | 13 |
| Estonian15 ^G^ | N1c | 14 | 14 | 16 | 23 | 11 | 15 | 14 |
| Karelian28 ^G^ | N1c | 14 | 14 | 16 | 23 | 12 | 14 | 14 |
| Estonian16 ^G^ | N1c | 14 | 14 | 16 | 24 | 10 | 14 | 13 |
| Estonian17 ^G^ | N1c | 14 | 14 | 16 | 24 | 10 | 14 | 14 |
| Karelian28 ^G^ | N1c | 14 | 14 | 16 | 24 | 10 | 14 | 14 |
| Karelian29 ^G^ | N1c | 14 | 14 | 16 | 24 | 10 | 14 | 14 |
| Karelian30 ^G^ | N1c | 14 | 14 | 16 | 24 | 10 | 14 | 14 |
| E Finnish42 ^G^ | N1c | 14 | 14 | 16 | 24 | 10 | 14 | 14 |
| E Finnish43 ^G^ | N1c | 14 | 14 | 16 | 24 | 10 | 14 | 14 |
| E Finnish44 ^G^ | N1c | 14 | 14 | 16 | 24 | 10 | 14 | 14 |
| E Finnish45 ^G^ | N1c | 14 | 14 | 16 | 24 | 10 | 14 | 14 |
| E Finnish46 ^G^ | N1c | 14 | 14 | 16 | 24 | 10 | 14 | 14 |
| W Finnish49 ^G^ | N1c | 14 | 14 | 16 | 24 | 10 | 14 | 14 |
| W Finnish50 ^G^ | N1c | 14 | 14 | 16 | 24 | 10 | 14 | 14 |
| W Finnish51 ^G^ | N1c | 14 | 14 | 16 | 24 | 10 | 14 | 14 |
| W Finnish52 ^G^ | N1c | 14 | 14 | 16 | 24 | 10 | 14 | 14 |
| W Finnish53 ^G^ | N1c | 14 | 14 | 16 | 24 | 10 | 14 | 14 |
| W Finnish54 ^G^ | N1c | 14 | 14 | 16 | 24 | 10 | 14 | 14 |
| E Finnish47 ^G^ | N1c | 14 | 14 | 16 | 24 | 10 | 14 | 15 |
| E Finnish48 ^G^ | N1c | 14 | 14 | 16 | 24 | 11 | 11 | 14 |
| E Finnish49 ^G^ | N1c | 14 | 14 | 16 | 24 | 11 | 11 | 14 |
| E Finnish50 ^G^ | N1c | 14 | 14 | 16 | 24 | 11 | 11 | 14 |
| E Finnish51 ^G^ | N1c | 14 | 14 | 16 | 24 | 11 | 13 | 14 |
| E Finnish52 ^G^ | N1c | 14 | 14 | 16 | 24 | 11 | 14 | 12 |
| Swedish10 ^G^ | N1c | 14 | 14 | 16 | 24 | 11 | 14 | 12 |
| E Finnish53 ^G^ | N1c | 14 | 14 | 16 | 24 | 11 | 14 | 13 |
| E Finnish54 ^G^ | N1c | 14 | 14 | 16 | 24 | 11 | 14 | 13 |
| E Finnish55 ^G^ | N1c | 14 | 14 | 16 | 24 | 11 | 14 | 13 |
| Estonian18 ^G^ | N1c | 14 | 14 | 16 | 24 | 11 | 14 | 14 |
| Estonian19 ^G^ | N1c | 14 | 14 | 16 | 24 | 11 | 14 | 14 |
| Karelian31 ^G^ | N1c | 14 | 14 | 16 | 24 | 11 | 14 | 14 |
| Karelian32 ^G^ | N1c | 14 | 14 | 16 | 24 | 11 | 14 | 14 |
| Karelian33 ^G^ | N1c | 14 | 14 | 16 | 24 | 11 | 14 | 14 |
| Karelian34 ^G^ | N1c | 14 | 14 | 16 | 24 | 11 | 14 | 14 |
| Karelian35 ^G^ | N1c | 14 | 14 | 16 | 24 | 11 | 14 | 14 |
| Karelian36 ^G^ | N1c | 14 | 14 | 16 | 24 | 11 | 14 | 14 |
| Karelian37 ^G^ | N1c | 14 | 14 | 16 | 24 | 11 | 14 | 14 |
| Karelian38 ^G^ | N1c | 14 | 14 | 16 | 24 | 11 | 14 | 14 |
| Karelian39 ^G^ | N1c | 14 | 14 | 16 | 24 | 11 | 14 | 14 |
| Karelian40 ^G^ | N1c | 14 | 14 | 16 | 24 | 11 | 14 | 14 |
| Karelian41 ^G^ | N1c | 14 | 14 | 16 | 24 | 11 | 14 | 14 |
| Karelian42 ^G^ | N1c | 14 | 14 | 16 | 24 | 11 | 14 | 14 |
| Karelian43 ^G^ | N1c | 14 | 14 | 16 | 24 | 11 | 14 | 14 |
| Karelian44 ^G^ | N1c | 14 | 14 | 16 | 24 | 11 | 14 | 14 |
| Karelian45 ^G^ | N1c | 14 | 14 | 16 | 24 | 11 | 14 | 14 |
| Karelian46 ^G^ | N1c | 14 | 14 | 16 | 24 | 11 | 14 | 14 |
| Karelian47 ^G^ | N1c | 14 | 14 | 16 | 24 | 11 | 14 | 14 |
| Karelian48 ^G^ | N1c | 14 | 14 | 16 | 24 | 11 | 14 | 14 |
| Karelian49 ^G^ | N1c | 14 | 14 | 16 | 24 | 11 | 14 | 14 |
| Karelian50 ^G^ | N1c | 14 | 14 | 16 | 24 | 11 | 14 | 14 |
| Karelian51 ^G^ | N1c | 14 | 14 | 16 | 24 | 11 | 14 | 14 |
| E Finnish56 ^G^ | N1c | 14 | 14 | 16 | 24 | 11 | 14 | 14 |
| E Finnish57 ^G^ | N1c | 14 | 14 | 16 | 24 | 11 | 14 | 14 |
| E Finnish58 ^G^ | N1c | 14 | 14 | 16 | 24 | 11 | 14 | 14 |
| E Finnish59 ^G^ | N1c | 14 | 14 | 16 | 24 | 11 | 14 | 14 |
| E Finnish60 ^G^ | N1c | 14 | 14 | 16 | 24 | 11 | 14 | 14 |
| E Finnish61 ^G^ | N1c | 14 | 14 | 16 | 24 | 11 | 14 | 14 |
| E Finnish62 ^G^ | N1c | 14 | 14 | 16 | 24 | 11 | 14 | 14 |
| E Finnish63 ^G^ | N1c | 14 | 14 | 16 | 24 | 11 | 14 | 14 |
| E Finnish64 ^G^ | N1c | 14 | 14 | 16 | 24 | 11 | 14 | 14 |
| E Finnish65 ^G^ | N1c | 14 | 14 | 16 | 24 | 11 | 14 | 14 |
| E Finnish66 ^G^ | N1c | 14 | 14 | 16 | 24 | 11 | 14 | 14 |
| E Finnish67 ^G^ | N1c | 14 | 14 | 16 | 24 | 11 | 14 | 14 |
| E Finnish68 ^G^ | N1c | 14 | 14 | 16 | 24 | 11 | 14 | 14 |
| E Finnish69 ^G^ | N1c | 14 | 14 | 16 | 24 | 11 | 14 | 14 |
| E Finnish70 ^G^ | N1c | 14 | 14 | 16 | 24 | 11 | 14 | 14 |
| E Finnish71 ^G^ | N1c | 14 | 14 | 16 | 24 | 11 | 14 | 14 |
| E Finnish72 ^G^ | N1c | 14 | 14 | 16 | 24 | 11 | 14 | 14 |
| E Finnish73 ^G^ | N1c | 14 | 14 | 16 | 24 | 11 | 14 | 14 |
| E Finnish74 ^G^ | N1c | 14 | 14 | 16 | 24 | 11 | 14 | 14 |
| E Finnish75 ^G^ | N1c | 14 | 14 | 16 | 24 | 11 | 14 | 14 |
| E Finnish76 ^G^ | N1c | 14 | 14 | 16 | 24 | 11 | 14 | 14 |
| E Finnish77 ^G^ | N1c | 14 | 14 | 16 | 24 | 11 | 14 | 14 |
| E Finnish78 ^G^ | N1c | 14 | 14 | 16 | 24 | 11 | 14 | 14 |
| E Finnish79 ^G^ | N1c | 14 | 14 | 16 | 24 | 11 | 14 | 14 |
| E Finnish80 ^G^ | N1c | 14 | 14 | 16 | 24 | 11 | 14 | 14 |
| E Finnish81 ^G^ | N1c | 14 | 14 | 16 | 24 | 11 | 14 | 14 |
| E Finnish82 ^G^ | N1c | 14 | 14 | 16 | 24 | 11 | 14 | 14 |
| E Finnish83 ^G^ | N1c | 14 | 14 | 16 | 24 | 11 | 14 | 14 |
| E Finnish84 ^G^ | N1c | 14 | 14 | 16 | 24 | 11 | 14 | 14 |
| E Finnish85 ^G^ | N1c | 14 | 14 | 16 | 24 | 11 | 14 | 14 |
| E Finnish86 ^G^ | N1c | 14 | 14 | 16 | 24 | 11 | 14 | 14 |
| E Finnish87 ^G^ | N1c | 14 | 14 | 16 | 24 | 11 | 14 | 14 |
| E Finnish88 ^G^ | N1c | 14 | 14 | 16 | 24 | 11 | 14 | 14 |
| E Finnish89 ^G^ | N1c | 14 | 14 | 16 | 24 | 11 | 14 | 14 |
| E Finnish90 ^G^ | N1c | 14 | 14 | 16 | 24 | 11 | 14 | 14 |
| E Finnish91 ^G^ | N1c | 14 | 14 | 16 | 24 | 11 | 14 | 14 |
| E Finnish92 ^G^ | N1c | 14 | 14 | 16 | 24 | 11 | 14 | 14 |
| E Finnish93 ^G^ | N1c | 14 | 14 | 16 | 24 | 11 | 14 | 14 |
| E Finnish94 ^G^ | N1c | 14 | 14 | 16 | 24 | 11 | 14 | 14 |
| E Finnish95 ^G^ | N1c | 14 | 14 | 16 | 24 | 11 | 14 | 14 |
| E Finnish96 ^G^ | N1c | 14 | 14 | 16 | 24 | 11 | 14 | 14 |
| E Finnish97 ^G^ | N1c | 14 | 14 | 16 | 24 | 11 | 14 | 14 |
| E Finnish98 ^G^ | N1c | 14 | 14 | 16 | 24 | 11 | 14 | 14 |
| E Finnish99 ^G^ | N1c | 14 | 14 | 16 | 24 | 11 | 14 | 14 |
| E Finnish100 ^G^ | N1c | 14 | 14 | 16 | 24 | 11 | 14 | 14 |
| E Finnish101 ^G^ | N1c | 14 | 14 | 16 | 24 | 11 | 14 | 14 |
| E Finnish102 ^G^ | N1c | 14 | 14 | 16 | 24 | 11 | 14 | 14 |
| E Finnish103 ^G^ | N1c | 14 | 14 | 16 | 24 | 11 | 14 | 14 |
| E Finnish104 ^G^ | N1c | 14 | 14 | 16 | 24 | 11 | 14 | 14 |
| E Finnish105 ^G^ | N1c | 14 | 14 | 16 | 24 | 11 | 14 | 14 |
| E Finnish106 ^G^ | N1c | 14 | 14 | 16 | 24 | 11 | 14 | 14 |
| E Finnish107 ^G^ | N1c | 14 | 14 | 16 | 24 | 11 | 14 | 14 |
| E Finnish108 ^G^ | N1c | 14 | 14 | 16 | 24 | 11 | 14 | 14 |
| W Finnish55 ^G^ | N1c | 14 | 14 | 16 | 24 | 11 | 14 | 14 |
| W Finnish56 ^G^ | N1c | 14 | 14 | 16 | 24 | 11 | 14 | 14 |
| W Finnish57 ^G^ | N1c | 14 | 14 | 16 | 24 | 11 | 14 | 14 |
| W Finnish58 ^G^ | N1c | 14 | 14 | 16 | 24 | 11 | 14 | 14 |
| W Finnish59 ^G^ | N1c | 14 | 14 | 16 | 24 | 11 | 14 | 14 |
| W Finnish60 ^G^ | N1c | 14 | 14 | 16 | 24 | 11 | 14 | 14 |
| W Finnish61 ^G^ | N1c | 14 | 14 | 16 | 24 | 11 | 14 | 14 |
| W Finnish62 ^G^ | N1c | 14 | 14 | 16 | 24 | 11 | 14 | 14 |
| W Finnish63 ^G^ | N1c | 14 | 14 | 16 | 24 | 11 | 14 | 14 |
| W Finnish64 ^G^ | N1c | 14 | 14 | 16 | 24 | 11 | 14 | 14 |
| W Finnish65 ^G^ | N1c | 14 | 14 | 16 | 24 | 11 | 14 | 14 |
| W Finnish66 ^G^ | N1c | 14 | 14 | 16 | 24 | 11 | 14 | 14 |
| Swedish11 ^G^ | N1c | 14 | 14 | 16 | 24 | 11 | 14 | 14 |
| Swedish12 ^G^ | N1c | 14 | 14 | 16 | 24 | 11 | 14 | 14 |
| Swedish13 ^G^ | N1c | 14 | 14 | 16 | 24 | 11 | 14 | 14 |
| Swedish14 ^G^ | N1c | 14 | 14 | 16 | 24 | 11 | 14 | 14 |
| Swedish15 ^G^ | N1c | 14 | 14 | 16 | 24 | 11 | 14 | 14 |
| Estonian20 ^G^ | N1c | 14 | 14 | 16 | 24 | 11 | 14 | 15 |
| E Finnish109 ^G^ | N1c | 14 | 14 | 16 | 24 | 11 | 14 | 15 |
| E Finnish110 ^G^ | N1c | 14 | 14 | 16 | 24 | 11 | 14 | 15 |
| E Finnish111 ^G^ | N1c | 14 | 14 | 16 | 24 | 11 | 14 | 15 |
| E Finnish112 ^G^ | N1c | 14 | 14 | 16 | 24 | 11 | 15 | 14 |
| E Finnish113 ^G^ | N1c | 14 | 14 | 16 | 24 | 11 | 15 | 14 |
| W Finnish67 ^G^ | N1c | 14 | 14 | 16 | 24 | 11 | 15 | 14 |
| Estonian21 ^G^ | N1c | 14 | 14 | 16 | 24 | 12 | 14 | 14 |
| E Finnish114 ^G^ | N1c | 14 | 14 | 16 | 24 | 12 | 14 | 14 |
| E Finnish115 ^G^ | N1c | 14 | 14 | 16 | 24 | 12 | 14 | 14 |
| E Finnish116 ^G^ | N1c | 14 | 14 | 16 | 24 | 12 | 14 | 14 |
| E Finnish117 ^G^ | N1c | 14 | 14 | 16 | 24 | 12 | 14 | 14 |
| E Finnish118 ^G^ | N1c | 14 | 14 | 16 | 24 | 12 | 14 | 14 |
| E Finnish119 ^G^ | N1c | 14 | 14 | 16 | 24 | 12 | 14 | 14 |
| E Finnish120 ^G^ | N1c | 14 | 14 | 16 | 24 | 12 | 14 | 14 |
| E Finnish121 ^G^ | N1c | 14 | 14 | 16 | 24 | 12 | 14 | 14 |
| E Finnish122 ^G^ | N1c | 14 | 14 | 16 | 24 | 12 | 14 | 14 |
| E Finnish123 ^G^ | N1c | 14 | 14 | 16 | 24 | 12 | 14 | 14 |
| E Finnish124 ^G^ | N1c | 14 | 14 | 16 | 24 | 12 | 14 | 14 |
| E Finnish125 ^G^ | N1c | 14 | 14 | 16 | 24 | 12 | 14 | 14 |
| W Finnish68 ^G^ | N1c | 14 | 14 | 16 | 24 | 12 | 14 | 14 |
| W Finnish69 ^G^ | N1c | 14 | 14 | 16 | 24 | 12 | 14 | 14 |
| Swedish16 ^G^ | N1c | 14 | 14 | 16 | 25 | 10 | 14 | 14 |
| Swedish17 ^G^ | N1c | 14 | 14 | 16 | 25 | 10 | 14 | 14 |
| E Finnish12 6 ^G^ | N1c | 14 | 14 | 16 | 25 | 11 | 14 | 13 |
| Karelian52 ^G^ | N1c | 14 | 14 | 16 | 25 | 11 | 14 | 14 |
| E Finnish127 ^G^ | N1c | 14 | 14 | 16 | 25 | 11 | 14 | 14 |
| E Finnish128 ^G^ | N1c | 14 | 14 | 16 | 25 | 11 | 15 | 14 |
| E Finnish129 ^G^ | N1c | 14 | 14 | 16 | 25 | 12 | 14 | 15 |
| Latvian01 ^G^ | N1c | 14 | 14 | 17 | 23 | 10 | 14 | 14 |
| Estonian22 ^G^ | N1c | 14 | 14 | 17 | 23 | 11 | 13 | 13 |
| Karelian53 ^G^ | N1c | 14 | 14 | 17 | 23 | 11 | 14 | 14 |
| E Finnish130 ^G^ | N1c | 14 | 14 | 17 | 23 | 11 | 14 | 14 |
| W Finnish70 ^G^ | N1c | 14 | 14 | 17 | 23 | 11 | 14 | 14 |
| E Finnish131 ^G^ | N1c | 14 | 14 | 17 | 23 | 12 | 14 | 15 |
| Karelian54 ^G^ | N1c | 14 | 14 | 17 | 24 | 10 | 14 | 14 |
| Karelian55 ^G^ | N1c | 14 | 14 | 17 | 24 | 10 | 14 | 14 |
| Estonian23 ^G^ | N1c | 14 | 14 | 17 | 24 | 10 | 15 | 15 |
| E Finnish132 ^G^ | N1c | 14 | 14 | 17 | 24 | 11 | 13 | 14 |
| Estonian24 ^G^ | N1c | 14 | 14 | 17 | 24 | 11 | 14 | 13 |
| E Finnish133 ^G^ | N1c | 14 | 14 | 17 | 24 | 11 | 14 | 13 |
| Karelian56 ^G^ | N1c | 14 | 14 | 17 | 24 | 11 | 14 | 14 |
| E Finnish134 ^G^ | N1c | 14 | 14 | 17 | 24 | 11 | 14 | 14 |
| E Finnish135 ^G^ | N1c | 14 | 14 | 17 | 24 | 11 | 14 | 14 |
| E Finnish136 ^G^ | N1c | 14 | 14 | 17 | 24 | 11 | 14 | 14 |
| E Finnish137 ^G^ | N1c | 14 | 14 | 17 | 24 | 11 | 14 | 14 |
| E Finnish138 ^G^ | N1c | 14 | 14 | 17 | 24 | 11 | 14 | 14 |
| W Finnish71 ^G^ | N1c | 14 | 14 | 17 | 24 | 11 | 14 | 14 |
| E Finnish139 ^G^ | N1c | 14 | 14 | 17 | 24 | 12 | 14 | 14 |
| Latvian02 ^G^ | N1c | 14 | 14 | 18 | 23 | 11 | 13 | 14 |
| Karelian57 ^G^ | N1c | 14 | 14 | 18 | 23 | 11 | 14 | 14 |
| Estonian25 ^G^ | N1c | 14 | 15 | 16 | 23 | 11 | 14 | 14 |
| E Finnish140 ^G^ | N1c | 14 | 15 | 16 | 24 | 10 | 14 | 14 |
| Karelian58 ^G^ | N1c | 14 | 15 | 16 | 24 | 11 | 14 | 14 |
| E Finnish141 ^G^ | N1c | 14 | 15 | 16 | 24 | 11 | 14 | 14 |
| W Finnish72 ^G^ | N1c | 14 | 15 | 16 | 25 | 12 | 14 | 14 |
| Estonian26 ^G^ | N1c | 14 | 15 | 17 | 24 | 11 | 14 | 14 |
| E Finnish142 ^G^ | N1c | 14 | 13 | 16 | 24 | 11 | 14 | 15 |
| E Finnish143 ^G^ | N1c | 14 | 13 | 16 | 24 | 11 | 14 | 15 |
| Estonian27 ^G^ | N1c | 14 | 13 | 16 | 24 | 11 | 15 | 13 |
| E Finnish144 ^G^ | N1c | 14 | 13 | 16 | 25 | 10 | 14 | 14 |
| E Finnish145 ^G^ | N1c | 14 | 14 | 15 | 24 | 11 | 14 | 14 |
| E Finnish146 ^G^ | N1c | 14 | 14 | 16 | 23 | 10 | 14 | 14 |
| W Finnish72 ^G^ | N1c | 14 | 14 | 16 | 23 | 10 | 14 | 14 |
| E Finnish147 ^G^ | N1c | 14 | 14 | 16 | 24 | 10 | 14 | 14 |
| E Finnish148 ^G^ | N1c | 14 | 14 | 16 | 24 | 10 | 14 | 14 |
| W Finnish73 ^G^ | N1c | 14 | 14 | 16 | 24 | 10 | 14 | 14 |
| Karelian59 ^G^ | N1c | 14 | 14 | 16 | 24 | 11 | 14 | 14 |
| E Finnish149 ^G^ | N1c | 14 | 14 | 16 | 24 | 11 | 14 | 14 |
| E Finnish150 ^G^ | N1c | 14 | 14 | 16 | 24 | 11 | 14 | 14 |
| E Finnish151 ^G^ | N1c | 14 | 14 | 16 | 24 | 11 | 14 | 14 |
| E Finnish152 ^G^ | N1c | 14 | 14 | 16 | 24 | 12 | 14 | 14 |
| E Finnish153 ^G^ | N1c | 14 | 14 | 16 | 24 | 12 | 14 | 14 |
| E Finnish154 ^G^ | N1c | 14 | 14 | 16 | 25 | 11 | 14 | 14 |
| E Finnish155 ^G^ | N1c | 14 | 13 | 16 | 23 | 12 | 14 | 14 |
| E Finnish156 ^G^ | N1c | 14 | 13 | 16 | 24 | 11 | 14 | 13 |
| E Finnish157 ^G^ | N1c | 14 | 13 | 16 | 24 | 11 | 14 | 15 |
| E Finnish158 ^G^ | N1c | 14 | 14 | 16 | 22 | 12 | 14 | 14 |
| E Finnish159 ^G^ | N1c | 14 | 14 | 16 | 23 | 10 | 14 | 14 |
| E Finnish160 ^G^ | N1c | 14 | 14 | 16 | 23 | 10 | 14 | 14 |
| E Finnish161 ^G^ | N1c | 14 | 14 | 16 | 24 | 10 | 14 | 14 |
| E Finnish162 ^G^ | N1c | 14 | 14 | 16 | 24 | 10 | 14 | 14 |
| E Finnish163 ^G^ | N1c | 14 | 14 | 16 | 24 | 11 | 14 | 14 |
| E Finnish164 ^G^ | N1c | 14 | 14 | 16 | 24 | 11 | 14 | 14 |
| E Finnish165 ^G^ | N1c | 14 | 14 | 16 | 24 | 11 | 14 | 14 |
| E Finnish166 ^G^ | N1c | 14 | 14 | 16 | 24 | 11 | 14 | 14 |
| E Finnish167 ^G^ | N1c | 14 | 14 | 16 | 24 | 11 | 14 | 14 |
| E Finnish168 ^G^ | N1c | 14 | 14 | 16 | 24 | 11 | 14 | 14 |
| E Finnish169 ^G^ | N1c | 14 | 14 | 16 | 24 | 11 | 14 | 14 |
| E Finnish170 ^G^ | N1c | 14 | 14 | 16 | 24 | 11 | 14 | 14 |
| E Finnish171 ^G^ | N1c | 14 | 14 | 16 | 24 | 11 | 14 | 14 |
| E Finnish172 ^G^ | N1c | 14 | 14 | 16 | 24 | 11 | 14 | 14 |
| E Finnish173 ^G^ | N1c | 14 | 14 | 16 | 24 | 11 | 14 | 14 |
| E Finnish174 ^G^ | N1c | 14 | 14 | 16 | 24 | 11 | 14 | 14 |
| E Finnish175 ^G^ | N1c | 14 | 14 | 16 | 24 | 11 | 14 | 14 |
| E Finnish176 ^G^ | N1c | 14 | 14 | 16 | 24 | 11 | 14 | 14 |
| E Finnish177 ^G^ | N1c | 14 | 14 | 16 | 24 | 11 | 15 | 14 |
| E Finnish178 ^G^ | N1c | 14 | 14 | 16 | 24 | 12 | 14 | 14 |
| E Finnish179 ^G^ | N1c | 14 | 14 | 16 | 24 | 12 | 14 | 14 |
| E Finnish180 ^G^ | N1c | 14 | 14 | 16 | 25 | 11 | 14 | 14 |
| E Finnish181 ^G^ | N1c | 14 | 14 | 16 | 25 | 11 | 14 | 14 |
| E Finnish182 ^G^ | N1c | 14 | 15 | 16 | 24 | 11 | 14 | 14 |
| E Finnish183 ^G^ | N1c | 14 | 13 | 16 | 24 | 12 | 14 | 15 |
| E Finnish184 ^G^ | N1c | 14 | 14 | 16 | 24 | 12 | 14 | 14 |
| Estonian28 ^G^ | N1c | 14 | 13 | 15 | 23 | 11 | 14 | 15 |
| W Finnish74 ^G^ | N1c | 14 | 13 | 16 | 23 | 10 | 14 | 13 |
| Swedish18 ^G^ | N1c | 14 | 13 | 16 | 23 | 10 | 14 | 14 |
| Latvian03 ^G^ | N1c | 14 | 13 | 16 | 23 | 11 | 14 | 13 |
| Latvian04 ^G^ | N1c | 14 | 13 | 16 | 23 | 11 | 14 | 14 |
| Karelian60 ^G^ | N1c | 14 | 13 | 16 | 23 | 11 | 14 | 14 |
| W Finnish75 ^G^ | N1c | 14 | 13 | 16 | 23 | 11 | 14 | 14 |
| E Finnish185 ^G^ | N1c | 14 | 13 | 17 | 24 | 11 | 14 | 13 |
| Karelian61 ^G^ | N1c | 14 | 14 | 15 | 24 | 11 | 14 | 14 |
| E Finnish186 ^G^ | N1c | 14 | 14 | 16 | 22 | 11 | 14 | 14 |
| Karelian62 ^G^ | N1c | 14 | 14 | 16 | 23 | 10 | 14 | 13 |
| Karelian63 ^G^ | N1c | 14 | 14 | 16 | 23 | 11 | 14 | 14 |
| E Finnish187 ^G^ | N1c | 14 | 14 | 16 | 23 | 11 | 14 | 14 |
| E Finnish188 ^G^ | N1c | 14 | 14 | 16 | 24 | 11 | 11 | 14 |
| E Finnish189 ^G^ | N1c | 14 | 14 | 16 | 24 | 11 | 14 | 13 |
| E Finnish190 ^G^ | N1c | 14 | 14 | 16 | 24 | 11 | 14 | 14 |
| E Finnish191 ^G^ | N1c | 14 | 14 | 16 | 24 | 11 | 14 | 14 |
| W Finnish76 ^G^ | N1c | 14 | 14 | 16 | 24 | 11 | 14 | 14 |
| W Finnish77 ^G^ | N1c | 14 | 14 | 16 | 24 | 11 | 14 | 14 |
| Swedish19 ^G^ | N1c | 14 | 14 | 16 | 24 | 11 | 14 | 15 |
| W Finnish78 ^G^ | N1c | 14 | 14 | 16 | 24 | 12 | 14 | 14 |
| W Finnish79 ^G^ | N1c | 14 | 14 | 16 | 24 | 12 | 14 | 14 |
| Karelian64 ^G^ | N1c | 14 | 14 | 17 | 23 | 10 | 14 | 14 |
| Estonian29 ^G^ | N1c | 14 | 14 | 17 | 24 | 10 | 14 | 13 |
| E Finnish192 ^G^ | N1c | 14 | 15 | 16 | 24 | 11 | 14 | 13 |
| E Finnish193 ^G^ | N1c | 14 | 15 | 16 | 24 | 11 | 14 | 14 |
| W Finnish80 ^G^ | N1c | 14 | 15 | 16 | 24 | 11 | 14 | 14 |
| E Finnish194 ^G^ | N1c | 14 | 14 | 16 | 23 | 11 | 14 | 15 |
| E Finnish195 ^G^ | N1c | 14 | 13 | 16 | 23 | 10 | 14 | 14 |
| Karelian65 ^G^ | N1c | 14 | 13 | 16 | 24 | 11 | 14 | 14 |
| W Finnish81 ^G^ | N1c | 14 | 14 | 16 | 23 | 11 | 14 | 14 |
| E Finnish196 ^G^ | N1c | 14 | 14 | 16 | 24 | 12 | 14 | 14 |
| Swedish20 ^G^ | N1c | 14 | 14 | 16 | 25 | 10 | 14 | 14 |
| W Finnish82 ^G^ | N1c | 14 | 14 | 17 | 24 | 11 | 14 | 14 |
| W Finnish83 ^G^ | N1c | 14 | 14 | 17 | 24 | 11 | 14 | 14 |
| E Finnish197 ^G^ | N1c | 14 | 14 | 16 | 24 | 11 | 14 | 14 |
| E Finnish198 ^G^ | N1c | 14 | 14 | 17 | 24 | 11 | 14 | 14 |
| W Finnish84 ^G^ | N1c | 14 | 14 | 16 | 23 | 10 | 14 | 14 |
| Swedish21 ^G^ | N1c | 14 | 14 | 16 | 24 | 10 | 14 | 14 |
| Latvian05 ^G^ | N1c | 15 | 13 | 16 | 22 | 10 | 14 | 14 |
| Latvian06 ^G^ | N1c | 15 | 13 | 16 | 22 | 10 | 14 | 14 |
| Estonian30 ^G^ | N1c | 15 | 13 | 16 | 23 | 10 | 14 | 14 |
| Lithuanian04 ^G^ | N1c | 15 | 14 | 16 | 23 | 10 | 14 | 14 |
| Lithuanian05 ^G^ | N1c | 15 | 14 | 16 | 23 | 10 | 14 | 14 |
| Lithuanian06 ^G^ | N1c | 15 | 14 | 16 | 23 | 11 | 14 | 14 |
| Lithuanian07 ^G^ | N1c | 15 | 14 | 16 | 23 | 11 | 14 | 14 |
| Latvian07 ^G^ | N1c | 15 | 13 | 16 | 23 | 11 | 14 | 14 |
| Estonian31 ^G^ | N1c | 15 | 14 | 16 | 23 | 11 | 15 | 13 |
| Lithuanian08 ^G^ | N1c | 15 | 14 | 16 | 23 | 11 | 15 | 14 |
| Lithuanian09 ^G^ | N1c | 15 | 14 | 16 | 23 | 10 | 14 | 14 |
| Lithuanian10 ^G^ | N1c | 15 | 14 | 16 | 23 | 10 | 14 | 14 |
| Lithuanian11 ^G^ | N1c | 15 | 14 | 16 | 23 | 10 | 15 | 14 |
| Lithuanian12 ^G^ | N1c | 15 | 14 | 16 | 23 | 11 | 14 | 15 |
| Lithuanian13 ^G^ | N1c | 15 | 12 | 16 | 23 | 11 | 14 | 14 |
| W Finnish85 ^G^ | N1c | 15 | 13 | 16 | 22 | 11 | 14 | 14 |
| Estonian32 ^G^ | N1c | 15 | 13 | 16 | 23 | 10 | 14 | 14 |
| Lithuanian14 ^G^ | N1c | 15 | 13 | 16 | 23 | 10 | 14 | 14 |
| W Finnish86 ^G^ | N1c | 15 | 13 | 16 | 23 | 10 | 14 | 14 |
| W Finnish87 ^G^ | N1c | 15 | 13 | 16 | 23 | 10 | 14 | 14 |
| W Finnish88 ^G^ | N1c | 15 | 13 | 16 | 23 | 10 | 14 | 14 |
| W Finnish89 ^G^ | N1c | 15 | 13 | 16 | 23 | 10 | 14 | 15 |
| Latvian08 ^G^ | N1c | 15 | 13 | 16 | 23 | 11 | 14 | 14 |
| Latvian09 ^G^ | N1c | 15 | 13 | 16 | 23 | 11 | 14 | 14 |
| Latvian10 ^G^ | N1c | 15 | 13 | 16 | 23 | 11 | 14 | 14 |
| Latvian11 ^G^ | N1c | 15 | 13 | 16 | 23 | 11 | 14 | 14 |
| Lithuanian15 ^G^ | N1c | 15 | 13 | 16 | 23 | 11 | 14 | 14 |
| Lithuanian16 ^G^ | N1c | 15 | 13 | 16 | 23 | 11 | 14 | 14 |
| Lithuanian17 ^G^ | N1c | 15 | 13 | 16 | 23 | 11 | 14 | 14 |
| Lithuanian18 ^G^ | N1c | 15 | 13 | 16 | 23 | 11 | 14 | 14 |
| Lithuanian19 ^G^ | N1c | 15 | 13 | 16 | 23 | 11 | 14 | 14 |
| Lithuanian20 ^G^ | N1c | 15 | 13 | 16 | 23 | 11 | 15 | 14 |
| E Finnish199 ^G^ | N1c | 15 | 13 | 16 | 23 | 12 | 14 | 13 |
| W Finnish90 ^G^ | N1c | 15 | 13 | 16 | 24 | 10 | 14 | 13 |
| W Finnish91 ^G^ | N1c | 15 | 13 | 16 | 24 | 10 | 14 | 14 |
| E Finnish200 ^G^ | N1c | 15 | 13 | 16 | 24 | 11 | 14 | 15 |
| Karelian66 ^G^ | N1c | 15 | 13 | 17 | 23 | 11 | 14 | 14 |
| Swedish22 ^G^ | N1c | 15 | 13 | 17 | 23 | 11 | 14 | 14 |
| Lithuanian21 ^G^ | N1c | 15 | 14 | 15 | 23 | 11 | 13 | 14 |
| Lithuanian22 ^G^ | N1c | 15 | 14 | 15 | 23 | 11 | 13 | 14 |
| Lithuanian23 ^G^ | N1c | 15 | 14 | 15 | 23 | 11 | 14 | 14 |
| E Finnish201 ^G^ | N1c | 15 | 14 | 15 | 24 | 10 | 14 | 14 |
| Latvian12 ^G^ | N1c | 15 | 14 | 16 | 22 | 11 | 14 | 14 |
| Lithuanian24 ^G^ | N1c | 15 | 14 | 16 | 23 | 10 | 14 | 14 |
| Lithuanian25 ^G^ | N1c | 15 | 14 | 16 | 23 | 10 | 14 | 14 |
| Estonian33 ^G^ | N1c | 15 | 14 | 16 | 23 | 10 | 14 | 14 |
| Latvian13 ^G^ | N1c | 15 | 14 | 16 | 23 | 10 | 14 | 14 |
| Latvian14 ^G^ | N1c | 15 | 14 | 16 | 23 | 10 | 14 | 14 |
| Latvian15 ^G^ | N1c | 15 | 14 | 16 | 23 | 10 | 14 | 14 |
| Lithuanian26 ^G^ | N1c | 15 | 14 | 16 | 23 | 10 | 14 | 14 |
| Lithuanian27 ^G^ | N1c | 15 | 14 | 16 | 23 | 10 | 14 | 14 |
| Lithuanian28 ^G^ | N1c | 15 | 14 | 16 | 23 | 10 | 14 | 14 |
| Lithuanian29 ^G^ | N1c | 15 | 14 | 16 | 23 | 10 | 14 | 14 |
| Lithuanian30 ^G^ | N1c | 15 | 14 | 16 | 23 | 10 | 14 | 14 |
| Lithuanian31 ^G^ | N1c | 15 | 14 | 16 | 23 | 10 | 14 | 14 |
| W Finnish92 ^G^ | N1c | 15 | 14 | 16 | 23 | 10 | 14 | 14 |
| Latvian16 ^G^ | N1c | 15 | 14 | 16 | 23 | 10 | 15 | 14 |
| Latvian17 ^G^ | N1c | 15 | 14 | 16 | 23 | 11 | 14 | 14 |
| Lithuanian32 ^G^ | N1c | 15 | 14 | 16 | 23 | 11 | 14 | 14 |
| Lithuanian33 ^G^ | N1c | 15 | 14 | 16 | 23 | 11 | 14 | 14 |
| Lithuanian34 ^G^ | N1c | 15 | 14 | 16 | 23 | 11 | 14 | 14 |
| Lithuanian35 ^G^ | N1c | 15 | 14 | 16 | 23 | 11 | 14 | 14 |
| Lithuanian36 ^G^ | N1c | 15 | 14 | 16 | 23 | 11 | 14 | 14 |
| Lithuanian37 ^G^ | N1c | 15 | 14 | 16 | 23 | 11 | 14 | 14 |
| Lithuanian38 ^G^ | N1c | 15 | 14 | 16 | 23 | 11 | 14 | 14 |
| Lithuanian39 ^G^ | N1c | 15 | 14 | 16 | 23 | 11 | 14 | 14 |
| Lithuanian40 ^G^ | N1c | 15 | 14 | 16 | 23 | 11 | 14 | 14 |
| Lithuanian41 ^G^ | N1c | 15 | 14 | 16 | 23 | 11 | 14 | 14 |
| Lithuanian42 ^G^ | N1c | 15 | 14 | 16 | 23 | 11 | 14 | 14 |
| Lithuanian43 ^G^ | N1c | 15 | 14 | 16 | 23 | 11 | 14 | 14 |
| Lithuanian44 ^G^ | N1c | 15 | 14 | 16 | 23 | 11 | 14 | 14 |
| Lithuanian45 ^G^ | N1c | 15 | 14 | 16 | 23 | 11 | 14 | 14 |
| Lithuanian46 ^G^ | N1c | 15 | 14 | 16 | 23 | 11 | 14 | 14 |
| Lithuanian47 ^G^ | N1c | 15 | 14 | 16 | 23 | 11 | 14 | 14 |
| Karelian67 ^G^ | N1c | 15 | 14 | 16 | 23 | 11 | 14 | 14 |
| Lithuanian48 ^G^ | N1c | 15 | 14 | 16 | 23 | 11 | 15 | 13 |
| Lithuanian49 ^G^ | N1c | 15 | 14 | 16 | 23 | 11 | 15 | 14 |
| Lithuanian50 ^G^ | N1c | 15 | 14 | 16 | 24 | 10 | 14 | 14 |
| Swedish23 ^G^ | N1c | 15 | 14 | 16 | 24 | 10 | 14 | 14 |
| Lithuanian5 1 ^G^ | N1c | 15 | 14 | 16 | 24 | 11 | 14 | 14 |
| Lithuanian52 ^G^ | N1c | 15 | 14 | 16 | 24 | 11 | 14 | 14 |
| Lithuanian53 ^G^ | N1c | 15 | 14 | 16 | 24 | 11 | 14 | 14 |
| E Finnish202 ^G^ | N1c | 15 | 14 | 16 | 24 | 11 | 14 | 14 |
| E Finnish203 ^G^ | N1c | 15 | 14 | 16 | 24 | 11 | 14 | 14 |
| W Finnish93 ^G^ | N1c | 15 | 14 | 16 | 24 | 11 | 14 | 14 |
| Lithuanian54 ^G^ | N1c | 15 | 14 | 16 | 24 | 11 | 14 | 15 |
| E Finnish204 ^G^ | N1c | 15 | 14 | 16 | 24 | 11 | 14 | 15 |
| Swedish23 ^G^ | N1c | 15 | 14 | 16 | 24 | 11 | 15 | 14 |
| E Finnish20 5 ^G^ | N1c | 15 | 14 | 16 | 24 | 12 | 14 | 14 |
| Lithuanian55 ^G^ | N1c | 15 | 14 | 17 | 23 | 10 | 14 | 14 |
| Lithuanian56 ^G^ | N1c | 15 | 14 | 17 | 23 | 11 | 14 | 14 |
| Lithuanian57 ^G^ | N1c | 15 | 14 | 17 | 23 | 11 | 14 | 14 |
| Lithuanian58 ^G^ | N1c | 15 | 14 | 17 | 23 | 11 | 14 | 14 |
| Lithuanian59 ^G^ | N1c | 15 | 14 | 17 | 23 | 11 | 14 | 14 |
| Lithuanian60 ^G^ | N1c | 15 | 14 | 17 | 23 | 12 | 15 | 14 |
| E Finnish206 ^G^ | N1c | 15 | 14 | 17 | 24 | 10 | 14 | 14 |
| Lithuanian61 | N1c | 15 | 14 | 17 | 24 | 11 | 14 | 14 |
| E Finnish207 ^G^ | N1c | 15 | 14 | 17 | 25 | 11 | 14 | 14 |
| Latvian18 ^G^ | N1c | 15 | 14 | 18 | 23 | 10 | 15 | 15 |
| E Finnish208 ^G^ | N1c | 15 | 13 | 16 | 23 | 11 | 14 | 13 |
| W Finnish94 ^G^ | N1c | 15 | 13 | 16 | 24 | 10 | 14 | 14 |
| E Finnish209 ^G^ | N1c | 15 | 14 | 16 | 24 | 11 | 13 | 14 |
| E Finnish210 ^G^ | N1c | 15 | 15 | 16 | 24 | 11 | 14 | 14 |
| E Finnish211 ^G^ | N1c | 15 | 13 | 16 | 23 | 11 | 14 | 14 |
| E Finnish212 ^G^ | N1c | 15 | 14 | 16 | 24 | 10 | 14 | 14 |
| E Finnish213 ^G^ | N1c | 15 | 14 | 15 | 25 | 12 | 14 | 14 |
| W Finnish95 ^G^ | N1c | 15 | 14 | 16 | 24 | 11 | 14 | 14 |
| Lithuanian62 ^G^ | N1c | 15 | 13 | 16 | 24 | 11 | 14 | 14 |
| Lithuanian63 ^G^ | N1c | 15 | 14 | 16 | 23 | 11 | 15 | 14 |
| Latvian19 ^G^ | N1c | 15 | 13 | 16 | 23 | 10 | 12 | 14 |
| Estonian34 ^G^ | N1c | 15 | 13 | 16 | 23 | 10 | 14 | 14 |
| Latvian20 ^G^ | N1c | 15 | 13 | 16 | 23 | 10 | 14 | 14 |
| Latvian21 ^G^ | N1c | 15 | 13 | 16 | 23 | 10 | 14 | 14 |
| Latvian22 ^G^ | N1c | 15 | 13 | 16 | 23 | 10 | 14 | 14 |
| Estonian35 ^G^ | N1c | 15 | 13 | 16 | 23 | 11 | 14 | 14 |
| Latvian23 ^G^ | N1c | 15 | 13 | 16 | 23 | 11 | 14 | 14 |
| Latvian24 ^G^ | N1c | 15 | 13 | 16 | 23 | 11 | 14 | 14 |
| Latvian25 ^G^ | N1c | 15 | 13 | 16 | 23 | 11 | 14 | 14 |
| Latvian26 ^G^ | N1c | 15 | 13 | 16 | 23 | 11 | 14 | 14 |
| Latvian27 ^G^ | N1c | 15 | 13 | 16 | 23 | 11 | 14 | 14 |
| Latvian28 ^G^ | N1c | 15 | 13 | 16 | 23 | 11 | 14 | 14 |
| Lithuanian64 ^G^ | N1c | 15 | 13 | 16 | 23 | 11 | 14 | 14 |
| Latvian29 ^G^ | N1c | 15 | 13 | 16 | 24 | 10 | 14 | 14 |
| Latvian30 ^G^ | N1c | 15 | 13 | 16 | 24 | 10 | 14 | 14 |
| Latvian31 ^G^ | N1c | 15 | 13 | 17 | 23 | 11 | 14 | 14 |
| Latvian32 ^G^ | N1c | 15 | 13 | 17 | 23 | 11 | 14 | 14 |
| Karelian68 ^G^ | N1c | 15 | 13 | 17 | 23 | 11 | 14 | 14 |
| Latvian33 ^G^ | N1c | 15 | 13 | 17 | 24 | 11 | 14 | 14 |
| Latvian34 ^G^ | N1c | 15 | 13 | 18 | 23 | 11 | 14 | 14 |
| Latvian35 ^G^ | N1c | 15 | 14 | 15 | 23 | 11 | 14 | 14 |
| Estonian36 ^G^ | N1c | 15 | 14 | 16 | 23 | 11 | 14 | 14 |
| Latvian36 ^G^ | N1c | 15 | 14 | 16 | 23 | 11 | 14 | 14 |
| Latvian37 ^G^ | N1c | 15 | 14 | 16 | 23 | 11 | 14 | 14 |
| Latvian38 ^G^ | N1c | 15 | 14 | 16 | 23 | 11 | 14 | 14 |
| Latvian39 ^G^ | N1c | 15 | 14 | 16 | 23 | 11 | 14 | 14 |
| Latvian40 ^G^ | N1c | 15 | 14 | 16 | 23 | 11 | 14 | 14 |
| Lithuanian65 ^G^ | N1c | 15 | 14 | 16 | 23 | 11 | 14 | 14 |
| Lithuanian66 ^G^ | N1c | 15 | 14 | 16 | 23 | 11 | 15 | 14 |
| Lithuanian67 ^G^ | N1c | 15 | 14 | 17 | 23 | 11 | 13 | 14 |
| Latvian41 ^G^ | N1c | 15 | 14 | 17 | 23 | 12 | 14 | 14 |
| E Finnish214 ^G^ | N1c | 15 | 15 | 16 | 24 | 11 | 15 | 13 |
| Estonian37 ^G^ | N1c | 15 | 13 | 16 | 23 | 11 | 14 | 14 |
| Estonian38 ^G^ | N1c | 15 | 13 | 16 | 23 | 11 | 14 | 13 |
| Estonian39 ^G^ | N1c | 15 | 13 | 16 | 23 | 11 | 14 | 14 |
| Karelian69 ^G^ | N1c | 15 | 13 | 16 | 23 | 11 | 14 | 14 |
| Latvian42 ^G^ | N1c | 15 | 14 | 16 | 23 | 10 | 14 | 14 |
| Lithuanian6 8 ^G^ | N1c | 15 | 14 | 16 | 23 | 11 | 15 | 13 |
| Latvian43 ^G^ | N1c | 15 | 13 | 16 | 23 | 10 | 14 | 14 |
| Latvian44 ^G^ | N1c | 15 | 13 | 16 | 23 | 10 | 14 | 14 |
| Lithuanian69 ^G^ | N1c | 15 | 13 | 16 | 23 | 11 | 15 | 15 |
| Lithuanian70 ^G^ | N1c | 15 | 13 | 17 | 23 | 10 | 15 | 14 |
| Lithuanian71 ^G^ | N1c | 15 | 14 | 17 | 23 | 11 | 14 | 14 |
| W Finnish96 ^G^ | N1c | 15 | 14 | 16 | 23 | 11 | 14 | 13 |
| Lithuanian72 ^G^ | N1c | 16 | 14 | 16 | 23 | 11 | 14 | 14 |
| Karelian70 ^G^ | N1c | 16 | 14 | 16 | 24 | 11 | 14 | 14 |
| Latvian45 ^G^ | N1c | 16 | 14 | 17 | 23 | 11 | 14 | 14 |
| E Finnish215 ^G^ | N1c | 16 | 14 | 17 | 24 | 10 | 14 | 14 |
| W Finnish97 ^G^ | N1c | 16 | 11 | 16 | 23 | 11 | 14 | 14 |
| Latvian46 ^G^ | N1c | 16 | 12 | 15 | 23 | 11 | 14 | 14 |
| Latvian47 ^G^ | N1c | 16 | 13 | 15 | 23 | 10 | 14 | 14 |
| Estonian40 ^G^ | N1c | 16 | 14 | 16 | 23 | 11 | 13 | 14 |
| Lithuanian73 ^G^ | N1c | 16 | 14 | 18 | 24 | 11 | 14 | 15 |

Note: A is from Hammer *et al*, 2006, B is from Cai *et al*, 2011, C is from Rootsi *et al*, 2007, and D is from Balanovsk *et al*, 2008; E is from Derenko *et al*, 2007, F is from Lappalainen *et al*, 2006, G is from Lappalainen *et al*, 2008.
