# Supplementary material for: Genetic Evidence of an East Asian Origin and Paleolithic Northward Migration of Y-chromosome Haplogroup N
Source: PLoS One. 2013 Jun 20;8(6):e66102. doi: 10.1371/journal.pone.0066102 (PMC3688714; doi:10.1371/journal.pone.0066102)
Supplement: Table S3 — The populations information used to constructe contour maps. (DOCX) [file pone.0066102.s003.docx]

Table S3. The populations information used to constructe contour maps.

| **Population** | **Sample size** | **N*-M231** | **N1*-LLY22g** | **N1a-M128** | **N1b-p43** | **N1c-M46** |
| --- | --- | --- | --- | --- | --- | --- |
| Western Europe | 186 | 0 | 0 | 0 | 0 | 0 |
| Southern Europe | 383 | 0 | 0 | 0 | 0 | 0 |
| Denmark | 194 | 0.00 | 0.00 | 0.00 | 0.00 | 0.50 |
| Iceland | 181 | 0.00 | 0.00 | 0.00 | 0.00 | 0.60 |
| Norway (pooled from 3 populations) | 484 | 0.00 | 0.00 | 0.00 | 0.00 | 5.17 |
| Sweden (pooled from 4 populations) | 441 | 0.00 | 0.00 | 0.00 | 0.00 | 7.94 |
| Finland (pooled from 11 populations) | 1199 | 0.00 | 0.00 | 0.00 | 0.00 | 60.22 |
| Estonia (pooled from 3 populations) | 327 | 0.00 | 0.00 | 0.00 | 0.00 | 31.81 |
| Latvia (pooled from 3 populations) | 199 | 0.00 | 0.00 | 0.00 | 0.00 | 41.71 |
| Lithuania (pooled from 3 populations) | 360 | 0.00 | 0.00 | 0.00 | 0.00 | 40.00 |
| Karelians (pooled from 9 populations) | 311 | 0.00 | 0.00 | 0.00 | 6.11 | 41.80 |
| Russians | 264 | 0.00 | 0.00 | 0.00 | 0.40 | 13.30 |
| Hungaria | 113 | 0.00 | 0.00 | 0.00 | 0.00 | 0.90 |
| Slovak | 263 | 0.00 | 0.00 | 0.00 | 0.00 | 3.00 |
| Poland | 93 | 0.00 | 0.00 | 0.00 | 0.00 | 4.30 |
| Ukraine | 326 | 0.00 | 0.00 | 0.00 | 0.00 | 46.01 |
| Khants | 57 | 0.00 | 0.00 | 0.00 | 52.00 | 26.00 |
| Dolgans | 67 | 0.00 | 0.00 | 0.00 | 11.90 | 22.40 |
| Tuvinians | 311 | 0.00 | 1.60 | 0.00 | 21.90 | 16.00 |
| Khakas | 181 | 0.00 | 2.80 | 0.60 | 17.10 | 28.20 |
| Yakut (pooled from 14 districts) | 215 | 0.00 | 0.00 | 0.00 | 4.10 | 81.90 |
| Buryat | 81 | 0.00 | 0.00 | 0.00 | 2.50 | 28.40 |
| Koreans | 75 | 0.00 | 1.30 | 1.30 | 0.00 | 0.00 |
| Uzbeks | 78 | 0.00 | 0.00 | 0.00 | 0.00 | 8.30 |
| Kazakhs (pooled from 3 locations) | 185 | 0.00 | 0.00 | 8.10 | 1.10 | 3.20 |
| Altai (North-west China) | 377 | 0.53 | 2.12 | 0.00 | 0.27 | 0.53 |
| Altai (North-east China) | 262 | 0.00 | 4.20 | 1.15 | 1.91 | 1.91 |
| Northern Han | 814 | 0.25 | 4.42 | 0.49 | 0.00 | 1.72 |
| Southern Han | 915 | 1.20 | 3.39 | 0.55 | 0.11 | 0.77 |
| Tibetans | 2354 | 0.04 | 5.52 | 0.08 | 0.04 | 0.42 |
| Tibeto-Burman | 325 | 0.62 | 7.38 | 3.08 | 0.00 | 0.92 |
| Hmong-Mien | 308 | 0.32 | 0.65 | 0.32 | 0.00 | 0.65 |
| Daic | 463 | 1.51 | 1.94 | 0.00 | 0.00 | 0.22 |
| Austro-Asiatic (south-west China) | 100 | 0.00 | 5.00 | 0.00 | 0.00 | 0.00 |
| Austro-Asiatic (Cambodian) | 365 | 0.00 | 0.27 | 0.00 | 0.00 | 0.00 |
| Iran | 82 | 0.00 | 0.00 | 0.00 | 0.00 | 0.00 |
| Pakistan | 176 | 0.00 | 0.00 | 0.00 | 0.00 | 0.00 |
| India | 1114 | 0.00 | 0.00 | 0.00 | 0.00 | 0.00 |
| Japanese | 237 | 0.00 | 0.00 | 0.00 | 0.00 | 0.00 |
| Vietnam | 80 | 0.00 | 0.00 | 0.00 | 0.00 | 0.00 |
| Laos | 778 | 0.00 | 0.00 | 0.00 | 0.00 | 0.00 |
| Philippines | 87 | 0.00 | 0.00 | 0.00 | 0.00 | 0.00 |
| Indonesia | 295 | 0.00 | 0.00 | 0.00 | 0.00 | 0.00 |
| Malaysia | 50 | 0.00 | 0.00 | 0.00 | 0.00 | 0.00 |
| Oceania | 753 | 0.00 | 0.00 | 0.00 | 0.00 | 0.00 |

Note: Data is from Hammer *et al*, 2006, Cai *et al*, 2011, Rootsi *et al*, 2007, Balanovsk *et al*, 2008, Derenko *et al*, 2007, Lappalainen *et al*, 2006, Lappalainen *et al*, 2008 and present study.
